# Cellular Allometry of Mitochondrial Functionality Establishes the Optimal Cell Size

**DOI:** 10.1016/j.devcel.2016.09.004

**Published:** 2016-11-07

**Authors:** Teemu P. Miettinen, Mikael Björklund

**Affiliations:** 1 Division of Cell and Developmental Biology, School of Life Sciences, University of Dundee, Dundee DD1 5EH, UK; 2 MRC Laboratory for Molecular Cell Biology, University College London, Gower Street, London WC1E 6BT, UK; 3 Koch Institute for Integrative Cancer Research, Massachusetts Institute of Technology, Cambridge, MA 02139, USA

**Keywords:** cell size, growth, mitochondria, allometry, mevalonate pathway, mitochondrial dynamics, organelle scaling, Drp1, fitness, metabolism

## Abstract

Eukaryotic cells attempt to maintain an optimal size, resulting in size homeostasis. While cellular content scales isometrically with cell size, allometric laws indicate that metabolism per mass unit should decline with increasing size. Here we use elutriation and single-cell flow cytometry to analyze mitochondrial scaling with cell size. While mitochondrial content increases linearly, mitochondrial membrane potential and oxidative phosphorylation are highest at intermediate cell sizes. Thus, mitochondrial content and functional scaling are uncoupled. The nonlinearity of mitochondrial functionality is cell size, not cell cycle, dependent, and it results in an optimal cell size whereby cellular fitness and proliferative capacity are maximized. While optimal cell size is controlled by growth factor signaling, its establishment and maintenance requires mitochondrial dynamics, which can be controlled by the mevalonate pathway. Thus, optimization of cellular fitness and functionality through mitochondria can explain the requirement for size control, as well as provide means for its maintenance.

## Highlights

• Mitochondrial functionality is highest in intermediate-sized cells in a population
• Mitochondrial membrane potential changes with cell size, not cell cycle
• Evidence for an optimal cell size, whereby functionality and fitness are maximized
• Mitochondrial dynamics and mevalonate pathway required for the optimal cell size

## Graphical Abstract

**Figure:**
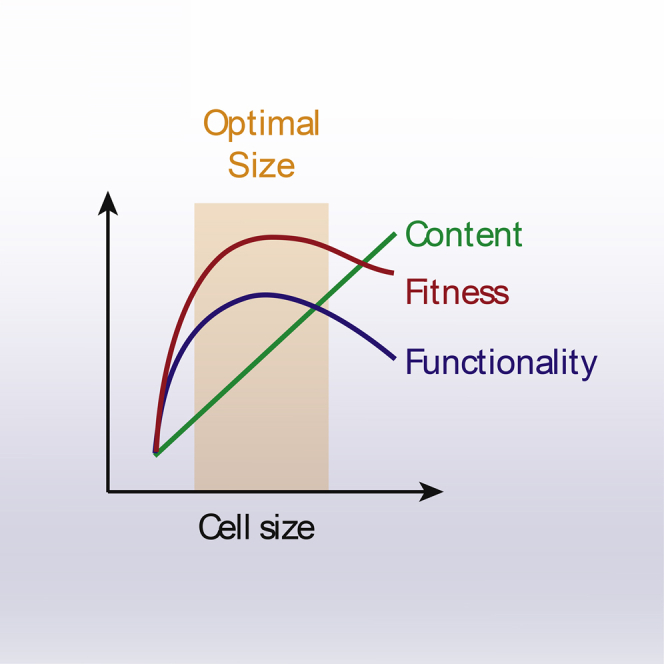

## Teaser

Organelle content scales linearly with cell size. Miettinen and Björklund investigate how this relates to organelle function and show that mitochondrial functionality and cellular fitness are highest at intermediate cell sizes, suggesting the existence of an optimal cell size. The mevalonate pathway contributes to cell size scaling of mitochondrial function.

## Introduction

**Figure 1:**
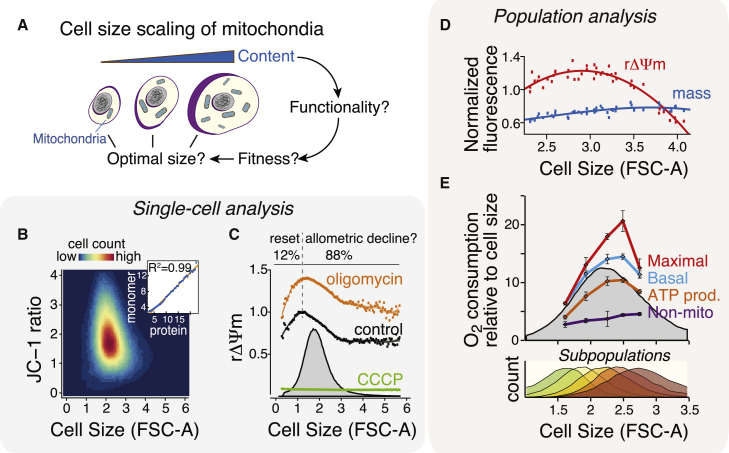
Cell Size Scaling of Mitochondrial Membrane Potential and Respiration Are Nonlinear (A) Cell size scaling of mitochondrial content is known to be linear, but how this translates to mitochondrial functionality is unknown. (B) Flow cytometry data of Kc167 cells stained with ratiometric mitochondrial membrane potential (ΔΨm) responsive JC-1 dye and plotted as a function of cell size (forward scatter, FSC-A). Inset shows the correlation between total cellular protein (DDAO-SE) and mitochondrial content (JC-1 monomer). (C) Computational cell size relation analysis for rΔΨm, the cell size normalized mitochondrial membrane potential. Untreated control cells as well as oligomycin- and CCCP-treated cells were stained with JC-1 and measured with flow cytometry. The cells were computationally fractionated by size and median ΔΨm was calculated for each size fraction (bin). Gray plot shows the size distribution of the cell population. Individual data points display the median rΔΨm for each cell size with line depicting the local polynomial regression curve (typical rΔΨm trajectory with cell size). The percentages of cells smaller (“reset phase”) and larger (“allometric decline”) in size than those with maximal rΔΨm are shown above the plot. n > 3.5 × 10^5^ cells for each sample. (D) Population-based analysis of mitochondrial content and rΔΨm with cell size. Kc167 cells were separated by centrifugal elutriation to size-based subpopulations. Mitochondrial content and ΔΨm were measured using MitoTracker green and red, respectively, and normalized to cell size (FSC-A). Three replicates for each subpopulation were measured and data were fitted using a trendline. (E) Oxygen consumption parameters for elutriated subpopulations (lower panel) were measured using the Seahorse Extracellular Flux analyzer and normalized to mean cell size of each subpopulation. Oxygen consumption data represent mean ± SD (n = 3–4). See also Figures S1 and S2.

For every cell type there is a typical cell size, possibly reflecting the size for optimal cellular functions (Ginzberg et al., 2015). Smaller size should be more efficient in nutrient uptake and metabolism due to greater surface-to-volume ratio. However, the very smallest cell size may not be ideal for cellular fitness and functionality. What determines the optimal cell size is not known. Cellular protein as well as organelle content typically scales linearly with cell size (Reber and Goehring, 2015, Schmoller and Skotheim, 2015), and it is commonly assumed that functionality increases linearly with total protein and organelle content. For example, the well-established linear mitochondrial content scaling (Kitami et al., 2012, Posakony et al., 1977, Rafelski et al., 2012) is considered a metabolic adaptation to increased cell size, as mitochondria are key organelles for growth and proliferation. However, it is difficult to reconcile how linear size scaling of mitochondrial functionality, which reflects the metabolic activity of the cell, could help in establishing an optimal cell size (Figure 1A). Instead, the presence of cell size-dependent changes in functionality and/or feedback to metabolic and growth processes have been considered critical for cell size homeostasis (Ginzberg et al., 2015, Schmoller and Skotheim, 2015).

Allometry is the study of biological scaling relative to organismal size with a key interest in the relationship between organismal metabolic rate and body mass. Allometric scaling laws were established through measurements of oxygen consumption (reflecting mitochondrial activity) and body mass. These experiments established that metabolic rate increases more slowly than body size. Thus larger organisms have less active oxidative metabolism per unit volume (Glazier, 2005, Kleiber, 1932). While allometry has been extensively studied at the organismal level, cellular allometry of metabolism and mitochondrial activity, i.e., how these functionalities scale with cell size, has not been carefully examined within the same cell population. Such analyses could be important for understanding the regulation of cell growth and cell size from evolutionary, biomedical, and biophysical perspectives.

Previous studies have shown that cellular growth rates are maximized in intermediate-sized phytoplankton species (Maranon, 2015) and that growth rates decline in the largest mammalian cells within a single population (Son et al., 2012, Tzur et al., 2009). These results suggest that cell growth may display nonlinear cellular allometry, whereby metabolic activity is reduced at both ends of the cell size range (Figure S1A). This is in stark contrast to the linear, isometric scaling of cellular protein and organelle content. Here we use both single-cell and population-based analyses to study the cell size scaling of mitochondrial functionality in animal cells. We show that cells within a single population exhibit an optimal cell size, whereby mitochondrial functionality and fitness are maximized.

## Results

### Mitochondrial Functionality Scales Nonlinearly with Cell Size

We recently reported that the relative expression of many mitochondrial genes declines in cells larger than normal size in cultured *Drosophila* cells and in mouse hepatocytes in vivo, although the mitochondrial content per volume unit did not change (Miettinen et al., 2014). Further analysis of proteome data from a leukemia cell line separated by cell size (Ly et al., 2014) showed that cell size scaling of proteins associated with different organelles, including mitochondria, scales isometrically (Figure S1B). As mitochondrial gene expression responds sensitively to functional demands, these data lead us to hypothesize that mitochondrial functional scaling could differ from the mitochondrial content scaling with cell size.

We wanted to analyze cell size scaling of mitochondrial functionality in a steady state, unperturbed cell population, where cell size differences reflect growth observed during the cell cycle. To achieve this aim, we used flow cytometry-based single-cell measurements together with JC-1 dye, which as a ratiometric reporter provides cell size normalized (relative) mitochondrial membrane potential (rΔΨm) measurements (see Experimental Procedures). In a steady state ΔΨm reflects the balance between the rate of electron transport and the rate of ATP utilization, thus providing a convenient measure for mitochondrial functionality. We also validated that the forward scatter (FSC-A) values provided by flow cytometry are accurate measurements of cell size (Figures S1C and S1B). The flow cytometry data consisting of cell size measurements (FSC-A) and rΔΨm are computationally fractionated into size-based subpopulations (bins) for which median rΔΨm is calculated and used to fit a local polynomial regression curve (loess) that visualizes the typical rΔΨm trajectory with cell size (Figure S2; see also Experimental Procedures). In our approach, each cell size bin corresponds to approximately 100 nm in cell diameter based on calibration data. To overcome the high cell-to-cell variability in ΔΨm (Figures 1B and S2), we used 10^5^–10^6^ cells for typical analysis.

Consistent with previous reports (Kitami et al., 2012, Posakony et al., 1977, Rafelski et al., 2012), mitochondrial mass (as indicated by the green JC-1 dye monomer fluorescence) increased linearly with *Drosophila* Kc167 cell size (Pearson correlation R^2^ = 0.99; Figures 1B [inset], S2F). However, rΔΨm displayed a sharp increase in the smallest cells followed by a slower decline toward larger cells (Figure 1C). Similar cell size scaling of rΔΨm was observed in primary (e.g., human umbilical vein endothelial cells [HUVECs]) and immortalized (e.g., Jurkat) cell types from various species and also when using tetramethylrhodamine ethyl ester (TMRE), another ΔΨm-responsive dye (Figures S2L, and S1E). The nonlinear scaling pattern of rΔΨm persisted when mitochondria were further polarized by blocking ATP synthase, and was lost when mitochondria were uncoupled (Figure 1C). Plasma membrane potential did not display similar scaling with cell size (Figure S1G).

We next examined the cell size scaling of mitochondrial functions by first separating Kc167 cells into size-based subpopulations using centrifugal elutriation. Mitochondrial mass, as measured by MitoTracker green dye, remained constant in different-sized cells after normalization to cell size. In contrast, the ΔΨm-dependent fluorescence of MitoTracker red dye displayed a substantial decrease in both the smallest and largest cells (Figure 1D). Consistent with the MitoTracker red data, direct analysis of oxygen consumption indicated that various parameters of mitochondrial respiration displayed a nonlinear cell size scaling, whereby mitochondrial respiration is highest in intermediate-sized cells (Figure 1E). These single-cell and population-level data indicate that cell size scaling of mitochondrial content and functionality are distinct from each other, as mitochondrial functionality is maximized in intermediate-sized cells.

### Scaling of Mitochondrial Membrane Potential Is Cell Size, Not Cell Cycle, Dependent

**Figure 2:**
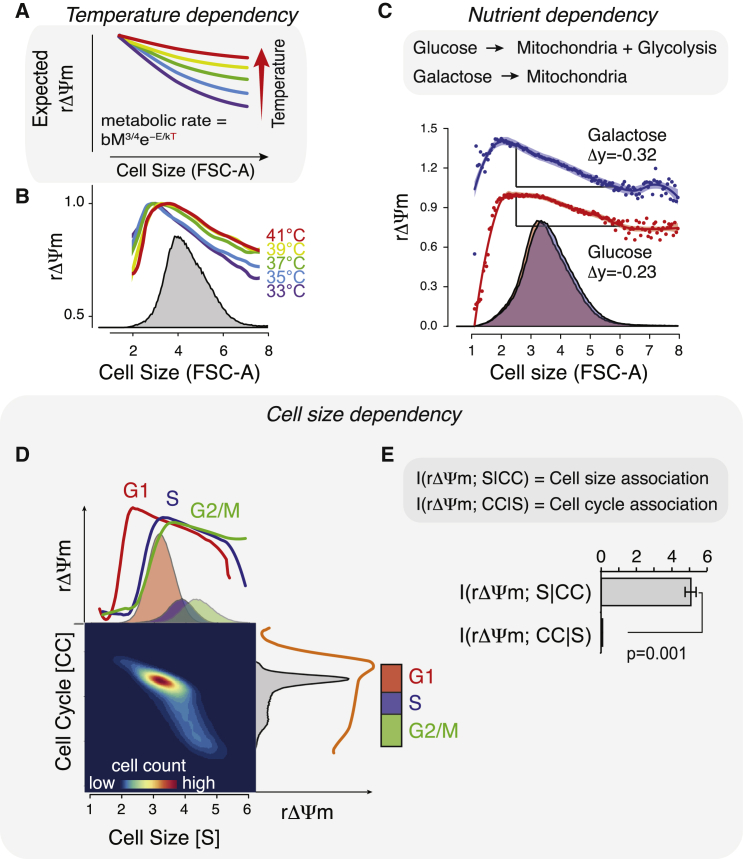
Cell Size Scaling of rΔΨm Is Affected by Temperature and Cellular Metabolism, but Not Cell Cycle (A) Expected effect of temperature on cell size scaling of rΔΨm. Metabolic theory predicts that metabolic rate changes not only with size but also with temperature (Gillooly et al., 2001), as indicated by the equation. b is a general scaling constant, M is mass, T is temperature, k is Boltzmann's constant, and E is the activation energy of metabolism. Thus, increased temperature is expected to cause a shallower decline in rΔΨm. (B) Jurkat cells were incubated in complete medium at different temperatures for 2 hr to adjust the metabolic rate followed by JC-1 staining-based analysis. Note that rΔΨm scaling does not change further at the highest temperatures as cells approach denaturating temperatures. n > 1 × 10^5^ for each temperature point. (C) Effect of mitochondrial nutrient source on cell size scaling of rΔΨm. Glucose-grown cells utilize both glycolysis and mitochondria for energy generation. Galactose-grown cells are reliant on mitochondrial metabolism. Jurkat cells were cultured on glucose-containing medium or adapted to grow on galactose followed by rΔΨm measurement. Δy values show the magnitude of rΔΨm decline in the linear range of the curve. (D) ΔΨm associates with cell size but not cell cycle. Jurkat cells were stained with JC-1 and NuclearRed ID DNA dye to visualize rΔΨm scaling with cell size in each cell-cycle phase (top left) and with DNA content (bottom right). (E) Quantification of conditional information analysis of ΔΨm association with cell size and cell cycle showing the strength of ΔΨm association with cell size (S) and cell cycle (CC) from Jurkat cells measured as shown in (D). Data shown represent mean ± SD (n = 3) with >7.5 × 10^5^ cells per replicate. See also Figure S3.

We reasoned that if the rΔΨm scaling is indeed cell size dependent and related to the allometric decline in metabolic rate, we should also observe temperature dependency as predicted by the Arrhenius equation (Gillooly et al., 2001) (Figure 2A). We measured the cell size scaling of rΔΨm at temperatures ranging from 33°C to 41°C in Jurkat cells and observed a stronger decline in rΔΨm scaling with lower temperatures, a result consistent with the theory (Figure 2B). In addition, *Drosophila* cells, which are cultured at 23.5°C, display a stronger decline in rΔΨm toward larger cells than Jurkat cells, which are cultured at 37°C (compare Figures 1C and 2B).

We next examined the nutrient dependency of rΔΨm scaling. We cultured Jurkat cells in galactose-containing medium to increase their dependency on mitochondrial activity. In comparison with Jurkat cells grown in glucose-containing medium, galactose-grown cells displayed higher rΔΨm in smaller cells and a slightly stronger decline in rΔΨm in larger cells (Figure 2C). These data suggests that the observed mitochondrial functional scaling is due to the mitochondria in smallest and largest cells being less active than their mid-sized counterparts, although mitochondria in all sizes of cells remain functional (see also Figure 1C). Thus, the rΔΨm decrease in larger cells could be explained by reduced metabolic activity caused by the increase in cell size, possibly through changes in surface-to-volume ratio. The low rΔΨm in the very smallest cells may be a “newborn effect,” whereby the small daughter cells are yet to reset their inherited low metabolic activity after mitosis.

As cells grow larger within the cell cycle, we next examined whether the ΔΨm changes are size specific or whether they could be better explained by the cell cycle. Analysis of human Jurkat cells and HUVECs stained for rΔΨm and DNA content indicated that rΔΨm declines also with the cell cycle, as expected. However, analysis of rΔΨm as a function of cell size separately in G_1_, S, and G_2_/M phases of the cell cycle revealed that rΔΨm scaling with cell size behaves similarly regardless of the cell-cycle phase (Figures 2D, S3B, and S3E). Comparison of the size-dependent change in rΔΨm within a single cell-cycle phase with the cell-cycle-dependent change in cells of the same size indicated that the rΔΨm change is 5–10 times greater with cell size than between the cell-cycle phases in Jurkat cells and HUVECs (Figures S3D and S3F). We also used Shannon's information theory (conditional mutual information) (Shannon, 1948) to quantify the association of ΔΨm with cell size = S, when cell cycle is fixed, i.e., I(ΔΨm, S|CC) and with cell cycle = CC, when cell size is fixed, i.e., I(ΔΨm, CC|S). rΔΨm displayed ∼40 times stronger association with cell size than the cell cycle in Jurkat cells (p = 0.001, t test) (Figure 2E). In conclusion, rΔΨm is dependent on cell size, not the cell cycle, as could be predicted from allometric laws (Glazier, 2005, Kleiber, 1932, Kozlowski et al., 2003).

### Cell Size Scaling of Mitochondrial Functions Sets the Optimal Cell Size

**Figure 3:**
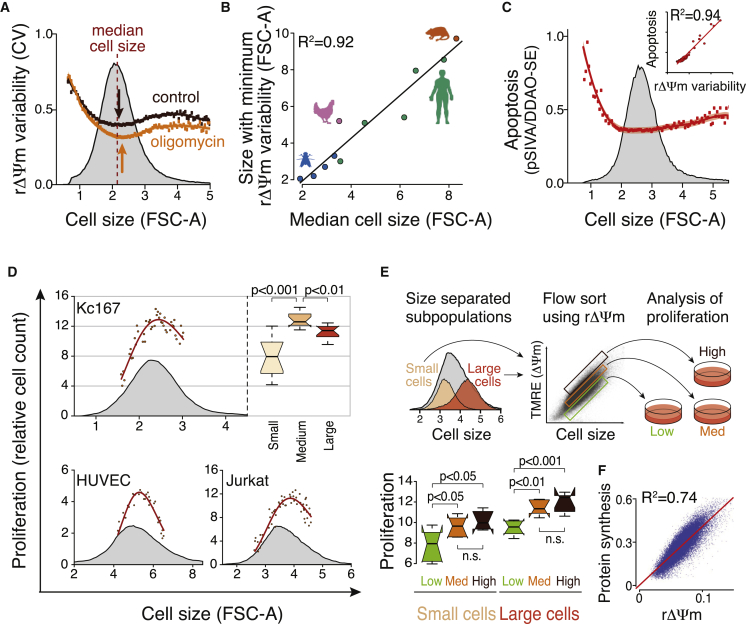
Cell Size Scaling of Mitochondrial Functions Establishes the Optimal Cell Size with Maximal Cellular Fitness (A) Minimum of ΔΨm variability associates with median cell size. Same data as in Figure 1B were analyzed for cell-to-cell variability in ΔΨm. CV, coefficient of variability. (B) Correlation between cell size with minimum ΔΨm variability and median cell size in cells of human (green), rat (orange), chicken (pink), and *Drosophila* (blue). (C) Levels of apoptotic cells as a function of cell size as analyzed by an annexin-based biosensor (pSIVA) and normalized for cell size using cellular protein content using amino-reactive DDAO-SE dye (Miettinen and Bjorklund, 2015). Inset: Pearson correlation of apoptosis with ΔΨm variability in each size of cell. n > 2.5 × 10^5^ cells. (D) Cell size scaling of overall cellular fitness, as measured by proliferative capacity. Kc167 cells, HUVECs, and Jurkat cells were separated to size-based subpopulations and equal numbers of cells were cultured for 72 hr (Kc167 and HUVECs) or 100 hr (Jurkat) before the final cell numbers were counted. Top right displays statistical analysis (ANOVA with Tukey's post hoc test) of Kc167 data (n = 12). (E) Effect of rΔΨm on cell proliferation. Jurkat cells were first separated by size and then by flow sorting to populations with different rΔΨm. Cells were cultured for 100 hr (n = 6) and analyzed as in (D). (F) Correlation between ΔΨm (MitoTracker red) and protein synthesis rate (OPP incorporation) in individual Jurkat cells (n = 3.1 × 10^4^ cells). Both fluorescence signals were normalized to cell size (FSC-A). See also Figure S4.

We next asked what are the consequences resulting from the uncoupling of mitochondrial content and function. Apoptosis and heterogeneity are determinants of mitochondrial and cellular fitness. We therefore examined whether metabolic heterogeneity (measured as coefficient of variability of ΔΨm) is minimized at any particular cell size and whether this is related to cellular fitness. Minimum rΔΨm variability was found to be associated with the median cell size in the Kc167 population using both JC-1 and TMRE dyes (Figures 3A and S1E). Similarly, there was a strong correlation between minimum variability and median cell size across primary and immortalized cell types from multiple organisms (Figure 3B). Measurement of apoptosis using phosphatidylserine exposure indicated that the number of dying cells increases especially in the smallest but also in the largest Kc167 cells (Figure 3C). These cells were early apoptotic cells, as our experiments exclude the cell population with compromised plasma membrane integrity (see Experimental Procedures). Furthermore, the cell size dependency pattern of apoptosis strongly correlated with the ΔΨm variability (R^2^ = 0.94, Figure 3C inset), supporting the concept that a low ΔΨm variability is beneficial for cellular fitness. These data, together with the cell size scaling of oxygen consumption, suggest that mitochondrial functions and fitness are optimized in intermediate-sized cells within a single population.

Cellular fitness is commonly evaluated by measuring cell proliferation, which for cells in nutrient-rich culture conditions can also be considered as a measure of the growth rate (production of cellular biomass). We separated Kc167 cells, HUVECs, and Jurkat cells into subpopulations with different cell sizes and thereafter allowed them to proliferate in complete culture medium. As with mitochondrial respiration (see Figure 1E), the average-sized cell populations appeared optimal, resulting in the highest number of cells (Figure 3D). Similar results were obtained when cells of different sizes were not physically separated but proliferation was analyzed using fluorescent dye dilution upon cell division (Miettinen and Bjorklund, 2015) (Figures S4A–S4C). To analyze the role of mitochondria more directly, we sorted Jurkat cells first by size using elutriation and then by rΔΨm using fluorescence-activated cell sorting, to obtain populations with low, medium, and high rΔΨm (Figure 3E). Within the same-sized cells, medium and high rΔΨm cells were more proliferative than low rΔΨm cells, indicating that mitochondrial activity promotes cellular fitness (Figure 3E). To further test the connection between rΔΨm and cellular fitness and growth rate, we measured Jurkat cell ΔΨm and protein synthesis rates with MitoTracker red and O-propargyl-puromycin (OPP), respectively. The rΔΨm explained up to 75% of the variability in protein synthesis rates (Figure 3F, R^2^ = 0.74), suggesting that mitochondrial activity is a key factor behind variability in cellular growth rate. Altogether, these experiments indicate that intermediate-sized cells within a population have the best cellular fitness, which is supported by optimal mitochondrial activity and minimal mitochondrial variability.

### Cells Aim to Maintain Their Optimal Cell Size

**Figure 4:**
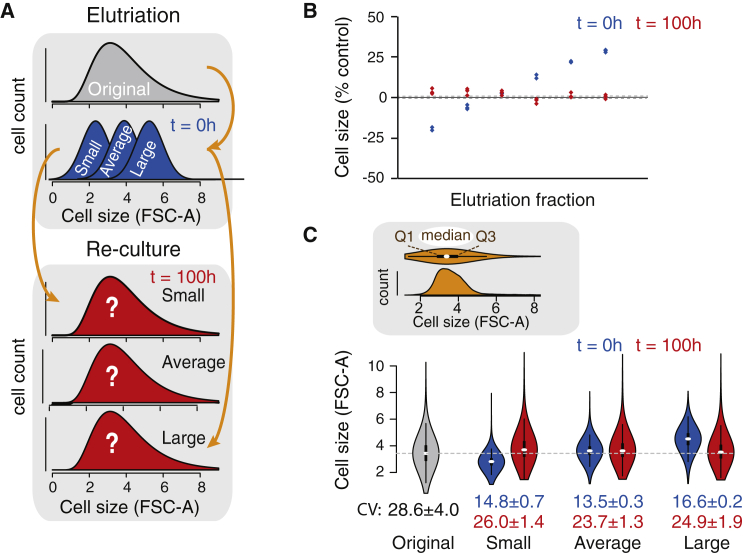
Cells Aim to Maintain Optimal Cell Size (A) Schematic of the experimental setup. Cells are separated into size-based subpopulations by elutriation and returned to culture. After 100 hr, mean cell size and size distribution are measured using flow cytometry to see whether the cell populations maintain their original median size and discover the resulting cell size distribution. (B) Mean cell size in each Jurkat cell subpopulation immediately after elutriation (0 hr, blue) and after 100 hr in culture (red). The data shown represent percent change versus unsorted original population. The size changes after 100 hr are statistically nonsignificant (two-tailed t test) (n = 3 in each subpopulation). (C) Size distributions of the original Jurkat cell population as well as smallest, average-sized, and largest subpopulations after 0 hr (blue) and 100 hr (red). The cell size distributions are illustrated with violin plots, and the inset above displays a comparison between a typical density plot and a violin plot representation of the data. White oval represents the median size and the thicker black line shows the first and third quartile (Q1 and Q3, where 25% and 75% of the data are below these points, respectively). CV is shown below the size distribution plots. Data shown represent mean ± SD (n = 3) from 1–5 × 10^4^ cells per sample. The gray dotted line indicates the median cell size in the original population. See also Figure S4.

We next analyzed whether cells separated by size return to their original size distribution, as this would be indicative of an optimal cell size (Figure 4A). Jurkat cells were elutriated into six fractions, and the mean sizes and size distributions of these subpopulations were measured at 0 hr and 100 hr. After 100 hr in culture, the median cell sizes of the subpopulations were indistinguishable from the median size of the original population (Figure 4B). As the resumption of normal cell size took a significant amount of time, the data suggest that potential cell size checkpoint(s) is/are not able to immediately reset cell size back to the optimal size. The narrower size distributions obtained by elutriation also broadened to resemble the original size distribution (Figure 4C). This widening of the size distribution also took place in the intermediate-sized subpopulation. While this might suggest that some cells are moving away from the optimal cell size, this widening of the size distribution mostly reflects the re-establishment of the normal cell-cycle distribution. This is because the selection of intermediate-sized cells by elutriation results in a cell population that is not only average in cell size but also highly enriched in cells in the middle of the cell cycle. Altogether, these data suggest that cells aim to maintain an optimal cell size at the population level. However, cells also maintain a size variation around the optimal cell size, as it is necessary to allow progression through the cell cycle. Similar results were observed in Kc167 cells (Figures S4D and S4E).

### Insulin Signaling Controls the Optimal Cell Size, but Not the Allometry, in Mitochondrial Functionality

**Figure 5:**
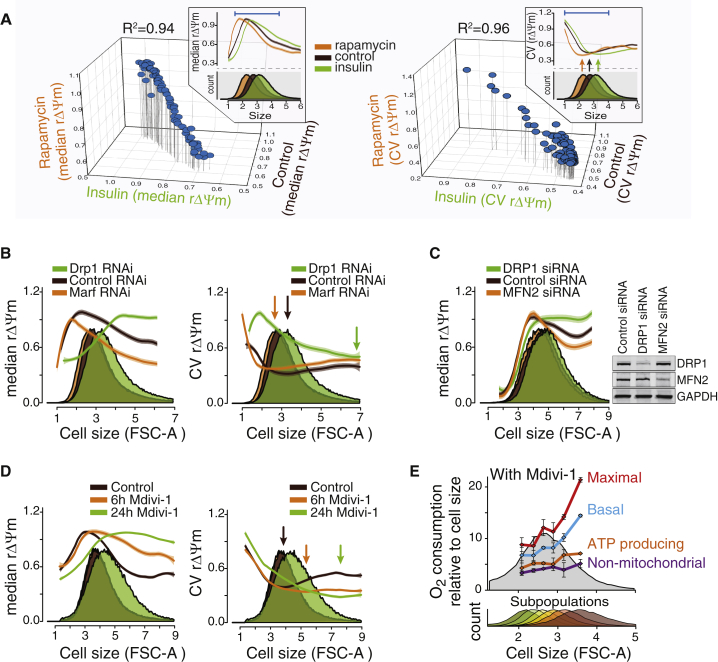
Mitochondrial Dynamics Regulate Cell Size Scaling of Mitochondrial Functions (A) Insulin signaling controls optimal cell size without changing the scaling of rΔΨm. Kc167 cells were treated with insulin and rapamycin for 14 hr and rΔΨm was analyzed with JC-1 staining. The 3D plots display median (left) and variability (right) of rΔΨm for each cell size bin after normalizing for the overall cell size change. Insets display size distribution and scaling of rΔΨm and its variability. n > 2 × 10^5^ cells. (B) Cell size scaling of rΔΨm (left) and its cell-to-cell variability (right) in Kc167 cells after 5 days of double-stranded RNA-mediated knockdown of Drp1 and Marf (mitofusin). Arrows indicate the cell size with the minimum variability. n > 1.7 × 10^5^ cells. (C) Cell size scaling of rΔΨm in Jurkat cells after 65 hr of small interfering RNA-mediated knockdown of DRP1 and MNF2 (mitofusin 2). Inset displays western blot of the knockdown efficiency. n > 1.2 × 10^5^ cells. (D) Kc167 cell rΔΨm (left) and variability (right) changes after 6 and 24 hr of treatment with the Drp1 inhibitor Mdivi-1. Arrows indicate the cell size with the minimum variability. n > 2.5 × 10^5^ cells. (E) The changes in the rΔΨm caused by Mdivi-1 translate into increased oxygen consumption in the largest cells. Kc167 cells treated with Mdivi-1 were separated by centrifugal elutriation to size-based subpopulations (bottom panel) from which oxygen consumption parameters were analyzed. Oxygen consumption data represent mean ± SD (n = 3–4). See also Figure S5.

Growth factor signaling is a key determinant in controlling average cell size at the population level in multicellular organisms, the insulin/mTOR (mammalian target of rapamycin) pathway being particularly important for cell size (Lloyd, 2013). To analyze how insulin signaling contributes to the optimal cell size and mitochondrial functional scaling, we treated Kc167 cells with insulin and rapamycin. While rapamycin decreased and insulin increased the cell size, scaling of mitochondrial functionality followed the new cell size distribution. The scaling remained nearly identical, as quantified by analyzing the correlations between cell size normalized rΔΨm medians and variabilities (R^2^ = 0.94 and 0.96, respectively, Figure 5A). Similar results were seen in Jurkat cells (Figure S5). Thus, insulin/mTOR signaling controls the average cell size but does not affect the scaling of mitochondrial functionality or the association of minimum rΔΨm variability with median cell size. Insulin signaling, and possibly growth factors in general, therefore controls the optimal cell size, but not necessarily its establishment and maintenance through mitochondria. It is possible that, as mTOR promotes mitochondrial biogenesis, mTOR activity has a much larger impact on the mitochondrial content than function.

### Mitochondrial Fusion and Fission Control the Cell Size Scaling of Mitochondrial Functions

We next wanted to identify which intrinsic processes are required for the observed allometric scaling of mitochondrial functionality. Changes in mitochondrial fusion and fission are tightly linked to ΔΨm (Chen et al., 2005, Legros et al., 2002) as well as mitochondrial homogeneity (Chan and Marshall, 2012). Furthermore, mitochondrial dynamics are predicted to affect cellular functions in a nonlinear manner (Hoitzing et al., 2015). We therefore asked whether mitochondrial fusion and fission affect the cell size scaling of mitochondrial functionality. Genetic inhibition of the mitochondrial dynamics by RNAi of the fission-regulating guanosine triphosphatase *Drp1* and the fusion-regulating mitofusins perturbed the cell size scaling of rΔΨm in Kc167 and Jurkat cells. Drp1 knockdown increased rΔΨm in larger cells, whereas mitofusin knockdown caused opposite effects (Figures 5B and 5C). As expected, a small-molecule inhibitor of mitochondrial division, Mdivi-1, induced effects similar to those of *Drp1* RNAi (Figure 5D). Inhibition of mitochondrial division also increased cell size. Furthermore, inhibition of mitochondrial fusion and fission also shifted the cell size with minimal ΔΨm variability (Figure 5B, right). These data suggest that mitochondrial dynamics or normal mitochondrial morphology is required for the typical cell size scaling of mitochondrial functions and the maintenance of optimal cell size. To further validate this, we analyzed Kc167 cell size scaling of mitochondrial respiration in the presence of Mdivi-1. In contrast to the untreated control cells (Figure 1E), Mdivi-1 treatment resulted in increased respiration in the largest cells (Figures 5E and S5F), as suggested by the changes in rΔΨm.

### The Mevalonate Pathway Is Required for Cell Size Scaling of Mitochondrial Functions

**Figure 6:**
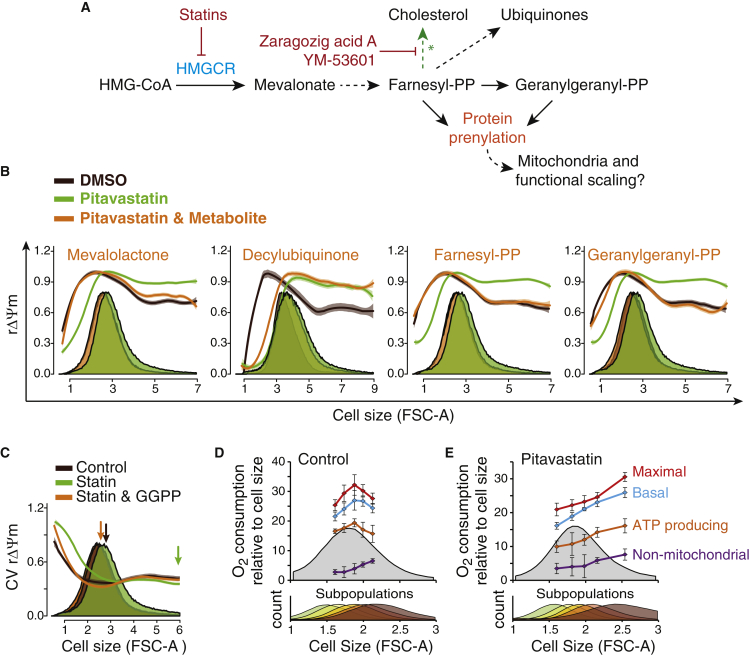
The Mevalonate Pathway-Mediated Protein Prenylation Is Required for Normal Cell Size Scaling of Mitochondrial Functions (A) Schematic of the mevalonate pathway. Chemical inhibitors used are shown in red. ^∗^Note that *Drosophila* cells are incapable of de novo cholesterol synthesis. (B) Mevalonate pathway regulates cell size scaling of rΔΨm through protein prenylation. Cell size scaling of rΔΨm in Kc167 cells treated with pitavastatin and indicated metabolites for 72 hr. n > 2 × 10^5^ cells. (C) Cell size scaling of rΔΨm variability after 72 hr of treatment with statin. The statin effects were fully rescued by GGPP. Arrows indicate the cell size with the minimum variability. n > 2 × 10^5^ cells. (D) Cell size scaling of respiration in control Kc167 cells. The experiment carried out as in Figure 1E. Data represent mean ± SD (n = 4). (E) Same as (D), but cells were treated with statin for 72 hr before the oxygen consumption assay. Data represent mean ± SD (n = 2–4). See also Figure S6.

The mevalonate pathway is important for cellular and organelle homeostasis by providing plasma membrane (cholesterol) and electron transport chain components (ubiquinones/coenzyme Q) as well as isoprenyls, which are required for post-translational modifications of proteins (Figure 6A). We previously identified the mevalonate pathway as a cell size regulator (Miettinen and Bjorklund, 2015, Miettinen et al., 2014), and this pathway has been shown to affect mitochondria (Andreux et al., 2014, Liu et al., 2014). We therefore examined how the mevalonate pathway affects the cell size scaling of mitochondrial functionality. In Kc167 cells inhibition of the mevalonate pathway by pitavastatin resulted in increased rΔΨm in larger cells (Figure 6B), consistent with the statin-induced ΔΨm increase in other cell types (Andreux et al., 2014). These effects were rescued by mevalonate, the metabolite immediately downstream of the statin target HMG-CoA reductase (Figure 6B). Further downstream of the mevalonate pathway the statin-induced scaling effects were rescued by farnesyl pyrophosphate and geranylgeranyl pyrophosphate (GGPP), isoprenyls required for protein prenylation (Figure 6B). Although ubiquinones are critical for mitochondrial electron transport chain function, the cell-permeable ubiquinone analog decylubiquinone did not rescue the statin phenotype (Figure 6B). Also, the statin-induced changes in rΔΨm cannot be due to cholesterol, as the Kc167 cells are incapable of de novo cholesterol synthesis. Consistently, inhibition of cholesterol synthesis downstream of farnesyl pyrophosphate did not change the cell size scaling of mitochondrial functionality (Figure S6A). Together, these results indicate that the mevalonate pathway and, more specifically, protein prenylation is required for the typical cell size scaling of rΔΨm observed in untreated cells.

We next investigated whether the mevalonate pathway could also affect the optimal cell size. Statin treatment dissociated the rΔΨm variability from the Kc167 median population cell size and this effect was rescued by GGPP supplementation (Figure 6C), suggesting that the mevalonate pathway is required for maintaining the optimal cell size. We validated this by measuring the cell size-dependent oxygen consumption for statin-treated Kc167 cells. In contrast to the control cells (Figure 6D), where the relative oxygen consumption was reduced in larger cells (as also seen in Figure 1E), statin treatment caused oxygen consumption to increase even in the very largest cells (Figure 6E).

We also examined if the altered cell size scaling of rΔΨm could be due to inhibition of late autophagy and consequential mitochondrial accumulation (Miettinen and Bjorklund, 2015). Inhibitors of autophagy did not change the cell size scaling of rΔΨm (Figure S6B), and pitavastatin and Mdivi-1 treatments increased mitochondrial content proportionally to cell size (Figure S6C). Thus, altered autophagy and mitophagy cannot explain the statin-induced phenotype.

We next performed time-course analyses of Mdivi-1 and statin-induced changes in ΔΨm variability and population cell size to examine the relationship between these aspects in more detail. Mdivi-1 linearly increased cell size and changes in rΔΨm (Figure S6D). In contrast, rΔΨm, its variability, and cell size responded to pitavastatin after a 24-hr delay, further suggesting that depletion of one or more prenylated proteins is necessary for these effects (Figure S6E). After this initial lag phase, both the cell size with minimum rΔΨm variability and the median population cell size slightly decreased, followed by a final increase. Overall, the median population cell size and rΔΨm changed with very similar time delays, indicating tight coupling of these processes. Finally, the statin-induced mitochondrial effects were not cell type or statin specific as they were also observed with another statin and in both Kc167 and Jurkat cells (Figure S6F). In conclusion, the mevalonate pathway is required for the cell size scaling of and the optimal cell size for mitochondrial functionality.

### The Mevalonate Pathway Regulates Mitochondrial Size and Morphology

**Figure 7:**
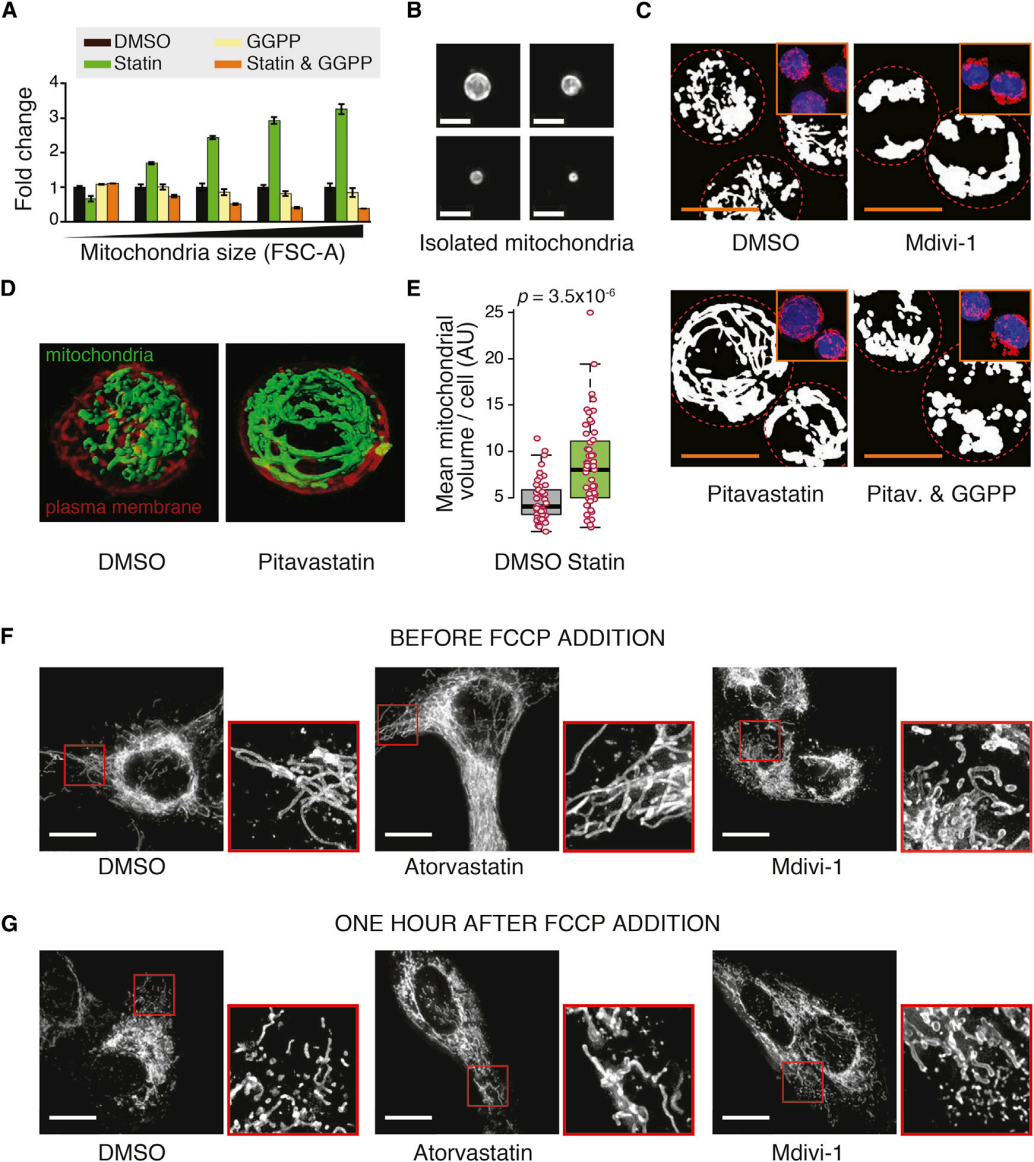
The Mevalonate Pathway Regulates Mitochondrial Volume and Morphology (A) Statins increase the size of isolated mitochondria. Flow cytometry-based size analysis of isolated mitochondria from Jurkat cells. Cells were treated with indicated chemicals for 72 hr. Data represent mean ± SD (n = 3). (B) Representative maximum-intensity projections of MitoTracker-green-stained, isolated mitochondria from Jurkat cells. Scale bars, 2.5 μm. (C) Representative binary images of Jurkat cell mitochondria. Insets show maximum-intensity projections of fixed Jurkat cells stained with DAPI (nucleus, blue) and MitoTracker red (mitochondria, red) after treatment with indicated chemicals. Statin treatment was for 72 hr and Mdivi-1 treatment for 12 hr. Scale bars, 10 μm. (D) Representative 3D projections of live Jurkat cell mitochondria after 72 hr of treatment with statin. Cells were stained with MitoTracker green and CellMask Deep Red plasma membrane stain. (E) Statins increase mitochondrial volume in live cells. Quantification of average mitochondrial volume within each Jurkat cell from samples shown in (D) (n = 48 [control] and 51 [pitavastatin], p value is from a t test). (F) Representative maximum-intensity projections, and their zoom-ins, of U2OS cells expressing mitochondria-targeted mCherry-GFP fusion after 72 hr of statin treatment or 24 hr of Mdivi-1 treatment. Scale bars, 10 μm. (G) Same as (F), except that cells were treated with 2 μM FCCP for 1 hr to induce mitochondrial fragmentation. Scale bars, 10 μm. See also Figure S7.

As the statin-induced mitochondrial functional scaling effects resembled those caused by Drp1 inhibition, we studied how the mevalonate pathway affects mitochondrial dynamics. We first isolated mitochondria from cells treated with statins and analyzed mitochondrial sizes using flow cytometry (Dabkowski et al., 2009, Song et al., 2015). Statin treatment of Jurkat and Kc167 cells increased the proportion of larger mitochondria, and this was prevented by addition of mevalonate or GGPP (Figures 7A and S7A). Microscopic observations validated that this analysis of isolated mitochondria could identify different-sized mitochondria, although the majority of them were round (Figure 7B). Thus, mitochondrial isolation allows size measurements but does not preserve mitochondrial morphology. We then examined the effects of mevalonate pathway inhibition on mitochondrial morphology using MitoTracker-red-stained and paraformaldehyde-fixed Jurkat and Kc167 cells. We found that the mevalonate pathway inhibition increases the number of enlarged tubular mitochondria (Figures 7C and S7B), consistent with observations in *Caenorhabditis elegans* (Liu et al., 2014). The morphology of statin-treated mitochondria was distinct from Mdivi-1-induced effects, and the statin effects were rescued by the addition of GGPP.

Mitochondrial morphology may be affected by fixation as well as mitochondrial dyes. To avoid such biases in our analysis, we further performed live cell experiments with Jurkat cells stained with MitoTracker green, which is structurally unrelated to MitoTracker red. MitoTracker-green-stained cells also displayed a more extensive tubular mitochondrial network in statin-treated cells than in control cells (Figures 7D and S7C). Quantitative analysis of mitochondrial volumes confirmed that statin-treated cells have significantly larger average mitochondrial size compared with controls (p = 3.5 × 10^−6^, two-tailed t test) (Figures 7E and S7D). This increase in mitochondrial size could, in theory, be due to inhibition of mitochondrial fragmentation. We therefore analyzed U2OS cells expressing mitochondria-targeted mCherry-GFP fusion protein (Allen et al., 2013) before and after disruption of the mitochondrial network by the mitochondrial uncoupler carbonyl cyanide 4-(trifluoromethoxy)phenylhydrazone (FCCP). U2OS cells display a well-connected mitochondrial network, which was efficiently fragmented by FCCP. This fragmentation could not be prevented, although it was potentially reduced in pitavastatin and Mdivi-1 treated cells (Figures 7F and 7G). Altogether, these analyses indicate that mitochondrial size and morphology is affected by the mevalonate pathway.

## Discussion

In contrast to the isometric cell size scaling of mitochondrial content, we report a nonproportional scaling of mitochondrial functions with cell size. Our results suggest that the physiological consequence of this nonlinear mitochondrial functional scaling is the establishment of an optimal cell size, as also supported by the known decline of growth rate in the largest cells (Maranon, 2015, Son et al., 2012, Tzur et al., 2009). These findings extend the allometric scaling laws on metabolism to the cellular level, as previously predicted (Kozlowski et al., 2003), and suggest that the allometric laws apply to the normal growth regime within a cell cycle. Nearly 20 years ago West, Brown, and Enquist provided a general model to explain allometric laws through transport of materials and energy through space-filling fractal networks of branching tubes (West et al., 1997). Only recently, it was shown that mitochondria may promote intracellular resource distribution by providing a conductive pathway for energy supply (Glancy et al., 2015). We now observe that the cellular allometry of mitochondrial metabolism is controlled by mitochondrial fusion and fission. It is thus possible that mitochondrial dynamics could influence cellular resource distribution by controlling the formation of a highly connected fractal-like mitochondrial network. This implies that a highly connected mitochondrial network could promote growth, improve fitness, and enable larger cell size by counteracting the biophysical limitations in resource distribution.

We also find that the mevalonate pathway affects mitochondrial dynamics and scaling of mitochondrial functionality. The mevalonate pathway, which produces various plasma membrane components, including cholesterol, could potentially act as a reporter for cell surface-to-volume ratio and relay this information to mitochondria via protein prenylation to maintain optimal cell size. It was recently shown that the levels of many proteins and metabolites in the mevalonate pathway are reduced in mitofusin knockout cells (Mourier et al., 2015), suggesting that mitochondrial dynamics can influence mevalonate pathway activity. This, together with our findings that the mevalonate pathway regulates cell size (Miettinen and Bjorklund, 2015) and mitochondrial dynamics, suggests that the mevalonate pathway and mitochondrial dynamics may constitute a growth and cell size regulatory network.

Finally, conditions such as cardiac hypertrophy and cellular aging are characterized by abnormal increases in cell size and reduced mitochondrial functionality. The potential implication of our current and previous (Miettinen and Bjorklund, 2015, Miettinen et al., 2014) observations is that changes in cell size may affect mitochondrial functionality. Thus, cell size may affect mitochondrial functions in various physiological and pathological settings (Lloyd, 2013, Nunnari and Suomalainen, 2012), making cell size-induced metabolic changes directly relevant for development and disease.

## Experimental Procedures

Full details are provided in Supplemental Experimental Procedures.

### Cell Culture and Chemical Treatments

Kc167 cells were cultured in Schneider's *Drosophila* medium supplemented with 10% fetal bovine serum (FBS), penicillin, and streptomycin. Jurkat cells were cultured in high-glucose RPMI supplemented with 10% FBS, penicillin, streptomycin, and L-glutamine. In addition, the following cell lines were used in some experiments: yeast BY4741 cells, *Drosophila* cells, Clone 8 and S2R^+^ cells, chicken DT40 cells, and human NB4 and U2OS stably expressing mCherry-GFP-FIS1 (amino acids 101–152) cells. Primary HUVECs and rat hepatocytes were from Life Technologies. All cell lines have been tested negative for mycoplasma. All chemicals were obtained from Sigma-Aldrich, unless otherwise stated. Concentrations used were 1 μM CCCP, 10 μM oligomycin, 50 μM Mdivi-1, 5 μM pitavastatin, 5 μM atorvastatin, 40 μM rosuvastatin, 20 μM decylubiquinone, 20 μM farnesyl pyrophosphate, 20 μM GGPP, and 0.2 mM mevalolactone. Note that the used statin concentrations exhibit only a modest toxicity in the cell lines used (Miettinen and Bjorklund, 2015), that cell toxicity does not explain the altered cell size scaling of rΔΨm (data not shown), and that the statin-induced rΔΨm effects were seen in *Drosophila* cells, which are incapable of de novo cholesterol synthesis.

### Fluorescence Probes

A common oversight in cell biology is that fluorescent markers are not normalized to cell size. We used JC-1 dye (Smiley et al., 1991) to measure ΔΨm (unless otherwise indicated), as the ratiometric nature of this probe inherently normalizes the data with mitochondrial content, but also to cell size due to isometric scaling of mitochondrial content (see Figures 1B and S2G). Thus JC-1 dye reports the relative (cell size normalized) ΔΨm (rΔΨm). In a typical experiment, JC-1 staining time was 45 min with 2 μM dye concentration. Stainings were performed at the normal cell-culture temperature for each cell line (23.5°C for *Drosophila* cells, 37°C for mammalian cells). All fluorescence intensities, cell counts, and sizes were measured using an Accuri C6 flow cytometer (Becton Dickinson).

### Computational Cell Size Relation Analysis of ΔΨm

To analyze the fluorescence signals, and thus cell behavior, as a function of cell size, we devised a new flow cytometer-based single-cell approach dubbed computational size relation analysis (CoSRA). Single-cell data falling into a viable cell gate, as estimated using plasma membrane integrity based on propidium iodide exclusion was used to define the viable cell population (Figure S2B). Data were exported from the flow cytometer and analyzed in R environment (R version 3.1.0). FL2-A/FL1-A ratio representing the JC-1 aggregate/monomer ratio was calculated for each cell and the cells were then computationally fractionated into size-based subpopulations (these bins are typically 5 × 10^4^ FSC-A units, which corresponds to approximately 100 nm in diameter based on calibration data presented in Figures S1C and S1D). Median fluorescence intensities were calculated for each bin. When comparing the cell size scaling of rΔΨm after different cellular perturbations, the median rΔΨm values were normalized to the bin with the highest median rΔΨm. Oligomycin, CCCP, and valinomycin treatments were normalized to the maximum of the control sample. A local polynomial regression (loess) curve was fitted to the median values using the built-in R function *loess*. The 95% confidence intervals for the loess curve were estimated using the predict function. Finally, medians (dots), loess regression line (solid curve), and confidence intervals for the regression line (shaded area) were plotted as a function of cell size (FSC-A) in the same plot with a cell size distribution histogram. The global minima of the variability were identified from the loess regression fit and indicated with vertical arrows. To increase figure clarity, we have removed individual data points (median or coefficient of variability [CV] of rΔΨm in each bin) from most figures. The resulting regression curve displays the typical trajectory of the measured parameter as a function of cell size. Note that the CoSRA approach is not limited to analyzing the cell size scaling of rΔΨm, as any cellular parameter for which a suitable fluorescence-based reporter is available can be used (see Figure S1 for examples).

### Analyzing the Contribution of Cell Size and Cell Cycle to the rΔΨm

To analyze the cell size dependency of ΔΨm, we applied mutual information analysis from Shannon's information theory. Shannon's information theory provides a general model by which to measure dependencies between variables, even if there is no linear dependence between these variables (Steuer et al., 2002). In information theory, mutual information is the average amount of information about U from observing the value of V. In particular, conditional mutual information expressed as I(U; V|W) provides an estimate of the quantity of information shared between U and V when W is known (fixed). We can thus estimate the association between rΔΨm and cell size (S), when the cell-cycle (CC) status of the cells is known, i.e., I(rΔΨm; S|CC). If cell size S carries information regarding rΔΨm, which is not already contained in CC information, the conditional mutual information yields a value >0. Zero value means lack of additional information between the variables rΔΨm and S, given that CC is known. Mutual information can thus have values of zero to infinity (0 … ∞); however, this value directly has no obvious interpretation, but by comparing I(rΔΨm; S|CC) and I(rΔΨm; CC|S), the relative information content carried by size and cell cycle can be analyzed.

### Size Separation by Centrifugal Elutriation

To examine size-dependent changes using population-based assays, we separated cells into size-based subpopulations using centrifugal elutriation. Approximately 3 × 10^8^ cells were resuspended in 3 mL of PBS with 1% FBS. Cells were loaded into a counterflow centrifugal elutriator (Beckman JE-5.0/JE), equipped with a standard elutriation chamber and a peristaltic pump (see Supplemental Experimental Procedures for details). Collected cells were resuspended in culture medium, and cell size and counts were analyzed using flow cytometry. Mitochondrial mass and membrane potential from elutriated fractions were measured using MitoTracker green FM and MitoTracker red CMXRos, respectively. Flow cytometry-measured MitoTracker intensities were normalized to mean FSC-A values.

### Oxygen Consumption Measurements

Oxygen consumption was measured using an XF24 Extracellular Flux Analyzer (Seahorse Bioscience). The assay plates were coated with poly-L-lysine, and 80,000 Kc167 cells were plated on each well 2 hr before the analysis. The analysis was carried out in Schneider's cell-culture medium with 10% FBS at room temperature. Oxygen consumption was measured every 6 min and the following injections were performed after every four measurements: (1) 1 μM oligomycin, (2) 1 μM FCCP, and (3) 2 μM rotenone and 2 μM antimycin A (see Supplemental Experimental Procedures for details). For each replicate the four oxygen consumption measurements between each injection were averaged, after which the respiratory parameters were calculated. All the results were normalized to the subpopulation cell size (FSC-A). The analysis was also carried out after treating cells with the mitochondrial division inhibitor Mdivi-1 (50 μM) for 24 hr or pitavastatin (5 μM) for 72 hr. Mdivi-1 and pitavastatin were present for the whole duration of the oxygen consumption assay.

### Proliferation Measurements

For proliferation-based fitness measurements, Kc167 cells, Jurkat cells, and HUVECs were first separated into size-based subpopulations using centrifugal elutriation. The average cell size in each subpopulation was measured using flow cytometry, and an equal cell count of each subpopulation was taken for further culture in normal conditions. The final data are presented as relative cell counts after indicated culture time (y axis) as a function of the initial subpopulation cell size (x axis). The total cell population histogram is also shown for reference. For Kc167 cells the differences in proliferative capacity of different-sized cells were highlighted by presenting the data as a box plot.

The effect of mitochondrial membrane potential on cell proliferation was analyzed in Jurkat cells by first using centrifugal elutriation to separate two different-sized subpopulations. These cells were then stained with TMRE and cells with high, medium, and low rΔΨm (thus taking cell size into account in assessing the ΔΨm, see schematic in Figure 2E) were separated using flow sorting (Influx Cell Sorter, Becton Dickinson), and cell sizes were reanalyzed in the separated populations to validate that rΔΨm-based separation did not change the average cell size. Equal cell counts of these rΔΨm-separated cells were then cultured for 100 hr in normal growth conditions, after which cells were recounted.

When analyzing cell proliferation using the CoSRA method (see above), cells were first stained using DDAO-SE dye and the baseline staining levels were measured using flow cytometry. The cells were then cultured normally for 48 hr after which cells were reanalyzed for DDAO-SE stain dilution (see Figure S5A).

### Protein Synthesis Measurements

Jurkat cells were stained with Click-iT Plus OPP Alexa Fluor 488 Protein Synthesis Assay Kit (LifeTechnologies) and MitoTracker red CMXRos. OPP and MitoTracker red were added to 20 μM and 200 nM final concentration, respectively, and incubated for 30 min. Cells were fixed with 3.7% formaldehyde and processed as instructed in the OPP assay kit. Cells were analyzed using flow cytometry.

### Microscopy

For examination of mitochondrial morphology, Kc167 and Jurkat cells were treated with indicated chemicals and moved to coverslips coated with 0.1% poly-L-lysine. The cells were stained with MitoTracker red CMXRos or MitoTracker green with CellMask Deep Red. U2OS cells expressing mCherry-GFP were imaged in the absence of any stains. For live cell imaging, cells were incubated at 37°C and 5% CO_2_ in complete culture medium during the experiment. Cells were imaged with a DeltaVision wide-field deconvolution microscope using standard filters (DAPI, fluorescein isothiocyanate, tetramethylrhodamine isothiocyanate, Cy5) and 100× objective. For details of image processing, see Supplemental Information.

### Statistical Analyses

Pearson correlations (R^2^) were calculated using the square of the *cor* function in R. When comparing the cell size scaling of rΔΨm after modulations to the insulin signaling pathway, the correlations between the median rΔΨm values in each cell size bin were calculated after normalizing for the cell size change caused by insulin or rapamycin. Statistical significances were calculated using one-way ANOVA and Tukey's post hoc test or t test as indicated. Conditional mutual information was calculated using the *condinformation* function in the infotheo package in R. The statistical significance of the mutual information values was assessed from replicate samples using a two-tailed t test.

## Author Contributions

T.P.M. and M.B. performed the experiments, analyzed the data, and wrote the paper. M.B. conceived the study.

## Supplemental Information

Document S1. Supplemental Experimental Procedures and Figures S1–S7

Document S2. Article plus Supplemental Information
