# Supplementary material for: Cellular Allometry of Mitochondrial Functionality Establishes the Optimal Cell Size
Source: Dev Cell. 2016 Nov 7;39(3):370–82. doi: 10.1016/j.devcel.2016.09.004 (PMC5104693; doi:10.1016/j.devcel.2016.09.004)
Supplement: Document S1. Supplemental Experimental Procedures and Figures S1–S7 [file mmc1.pdf]

Developmental Cell, Volume *39*

Supplemental Information

Cellular Allometry of Mitochondrial Functionality Establishes the Optimal Cell Size

Teemu P. Miettinen and Mikael Björklund

INVENTORY OF SUPPLEMENTARY MATERIALS:

Figure S1. Cellular organelle contents scales isometrically with cell size and flow cytometer FSC-A value provides accurate measurements of cell size, related to Figure 1.

Figure S2. Establishing computational size relation analysis (CoSRA) for rΔΨm in different cell types, related to Figure 1.

Figure S3. Cell size scaling of rΔΨm in different cell cycle phases, related to Figure 2.

Figure S4. Analysis of optimal cell size by dye dilution and elutriation, related to Figures 3 and 4.

Figure S5. Mitochondrial dynamics and not mTOR regulate cell size scaling of mitochondrial functions , related to Figure 5.

Figure S6. Cell size scaling of rΔΨm requires the mevalonate pathway and cannot be explained by increased mitophagy in larger cells, related to Figure 6.

Figure S7. Mitochondrial size and morphology homeostasis require the mevalonate pathway activity, related to Figure 7.

Supplemental Experimental Procedures

Supplemental References

SUPPLEMENTAL FIGURES

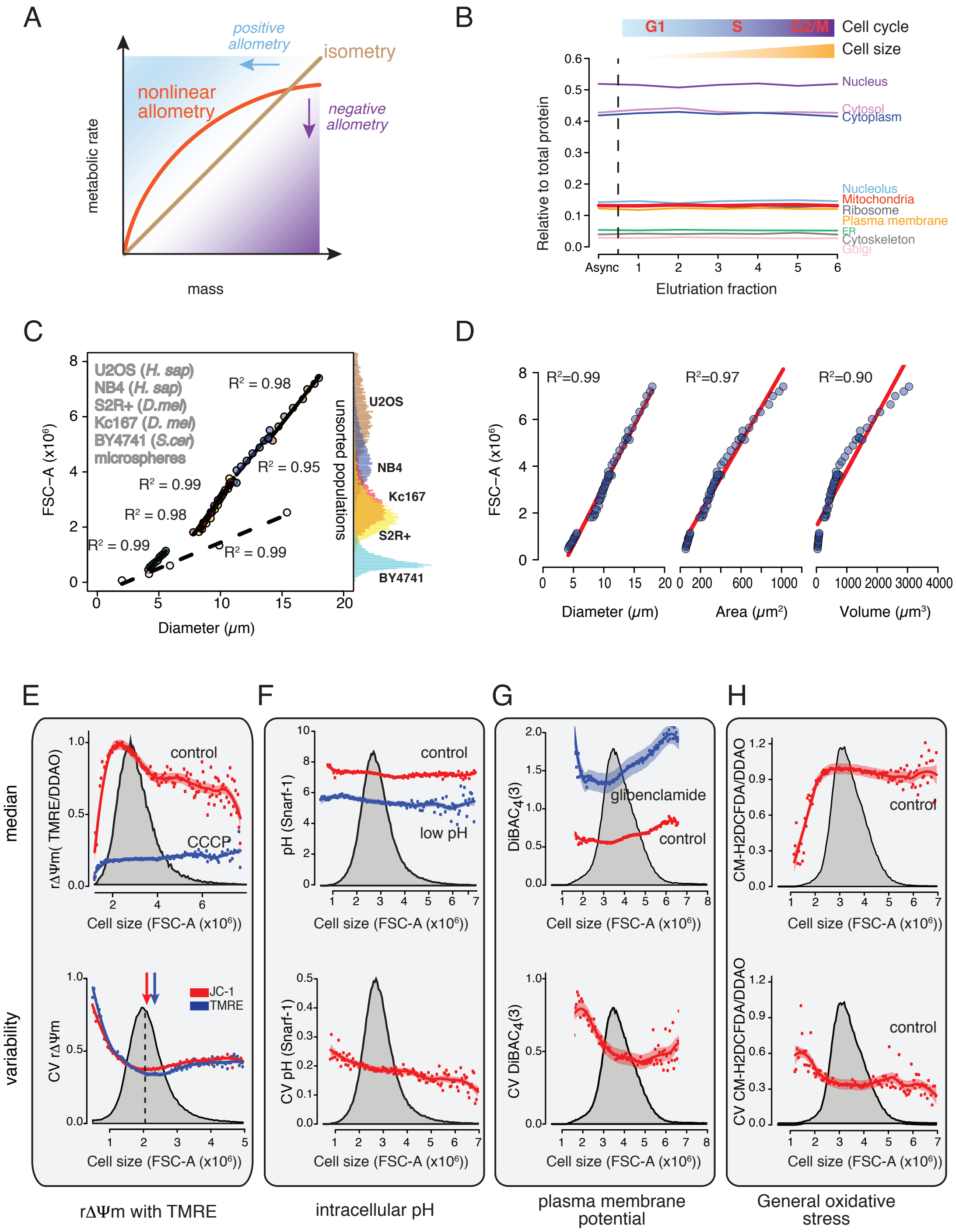

Figure S1. Cellular organelle contents scales isometrically with cell size and flow cytometer FSC-A value provides accurate measurements of cell size, related to Figure 1.

(A) Schematic presentation of different types of size scaling. Scaling where Y, for example metabolic rate, increases proportionally with mass (X) is isometric, whereas non-proportional scaling is allometric.

(B) Cell size scaling of relative proteome content associated with different subcellular compartments and organelles in NB4 leukemic cells. The data was normalised to the total protein content in the absence of absolute quantifications of protein levels. All organelle/protein groups examined display, on average, isometric cell size scaling during the normal cell cycle in an unperturbed cell population.

(C) Cultures of U2OS (human), NB4 (human), Kc167 (fruit fly), S2R+ (fruit fly) and BY4741 (yeast) cells were separated to different sized subpopulations using centrifugal elutriation. The mean size of each subpopulation was then assessed using electronic volume measurements (Casy TT) and flow cytometer measurements. Pearson correlations of the cell diameter, as measured using electronic volume measurements (x-axis), with FSC-A values (y-axis) are shown for each cell type assuming that cell size is spherical. In addition, data from polystyrene microbeads of known sizes are shown (light yellow markers and dashed line). Distributions of unsorted cell populations, as measured by flow cytometry, are shown on the right side of the plot.

(D) Correlations between FSC-A and electronic volume measurements based on cell diameter, area and volume from all cell measurements shown in (C). R^2 depicts Pearson correlation coefficient. Note, that although FSC-A is linearly best correlated with the cell diameter, absolute values of FSC-A may change over longer time periods due to instrument optical drift and thus cannot be directly used to obtain absolute cell sizes.

(E) Cell size scaling of r$\Delta\Psi$m as measured by TMRE and DDAO-SE staining. Kc167 cells were stained with DDAO-SE to label cellular protein content, after which $\Delta\Psi$m was measured using TMRE staining. TMRE was normalised to cellular protein content. Control and 1μM CCCP treated cells are shown. Bottom panel displays the cell size scaling of $\Delta\Psi$m cell-to-cell variability. Median cell size is indicated with a dotted black line, while arrows indicate the location of the minimum variability. Note that the ratiometric measurement of JC-1 normalises the $\Delta\Psi$m to mitochondrial mass, but as similar results are obtained without normalisation with TMRE. This suggests that the location of variability minimum depends on $\Delta\Psi$m, not mitochondrial content.

(F) Cell size scaling of intracellular pH was measured by staining Kc167 cells with SNARF-1 (top panel). Low pH control was made by incubating the cells in pH 5.5 buffer in the presence of valinomycin and nigericin for 10 min. pH was calibrated using the Intracellular pH Calibration Buffer Kit (Invitrogen). Note that SNARF-1 is a ratiometric dye and therefore further normalization to cell size is not required. Bottom panel displays the cell size scaling of intracellular pH cell-to-cell variability.

(G) Cell size scaling of plasma membrane potential ($\Delta\Psi p$) was measured by staining Jurkat cells with $DiBAC_4(3)$ (top panel). $DiBAC_4(3)$ entry into the cells is inhibited by high $\Delta\Psi p$. The negative control, 50μM glibenclamide, depolarizes plasma membrane and allows $DiBAC_4(3)$ entry. The results were normalised to DDAO-SE staining. $DiBAC_4(3)$ staining in Kc167 cells results in more linear cell size scaling of $\Delta\Psi p$, but glibenclamide was ineffective in depolarizing the Kc167 cell plasma membrane (data not shown). Bottom panel displays the cell size scaling of $\Delta\Psi p$ cell-to-cell variability.

(H) Cell size scaling of general oxidative stress was measured in Jurkat cells by CM-H2DCFDA staining which was normalized to total protein using content (DDAO-SE staining). Bottom panel displays the cell size scaling of oxidative stress cell-to-cell variability.

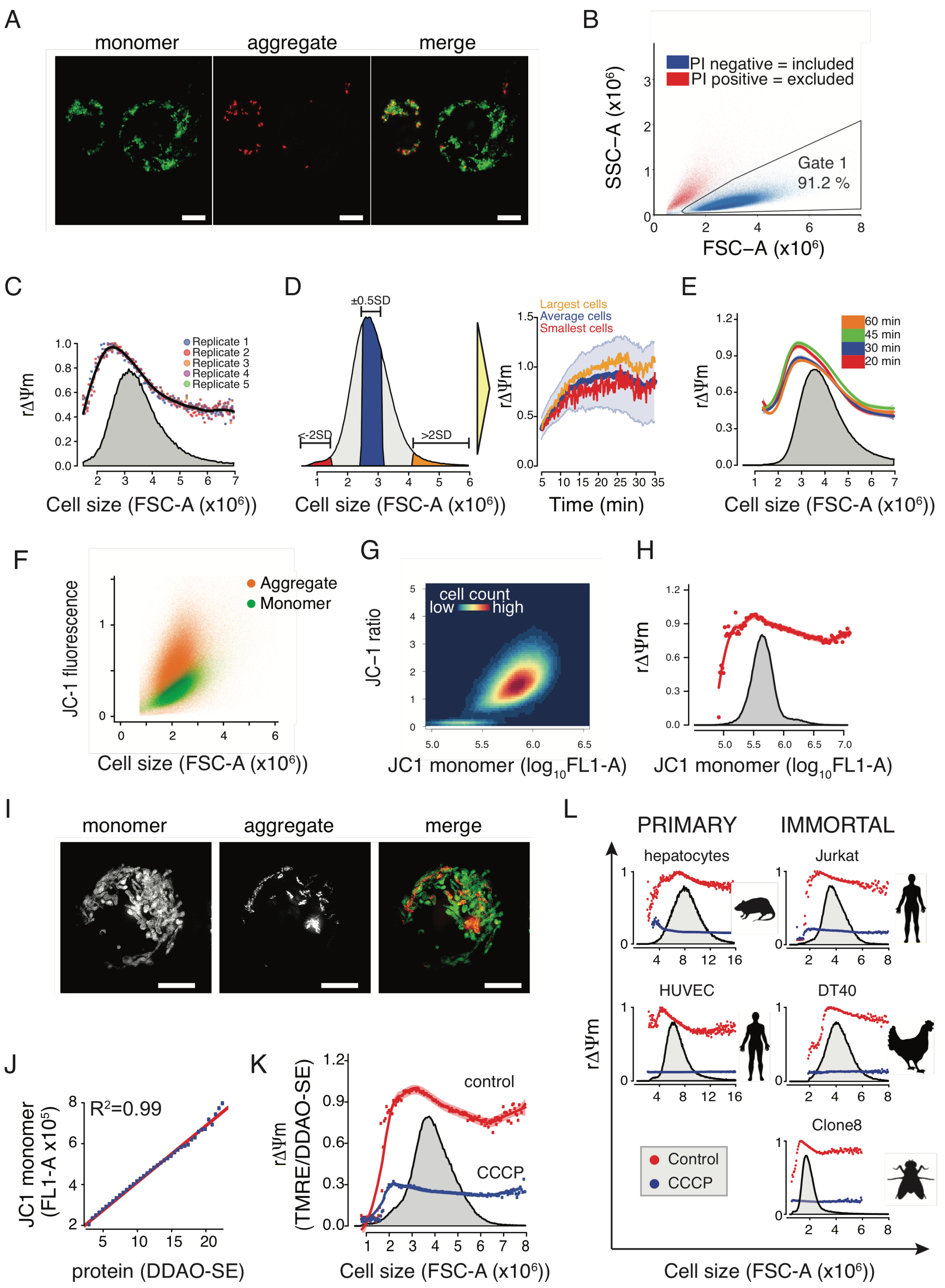

Figure S2. Establishing computational size relation analysis (CoSRA) for rΔΨm in different cell types, related to Figure 1.

(A) Maximum intensity projections of a small and a large Kc167 cell stained with JC-1 and imaged using OMX structural illumination microscopy. Monomer form (green) of the dye localizes to mitochondria and forms aggregates (red) in $\Delta\Psi m$ dependent manner. The ratio between the monomer and the aggregate in each cell reports the cell size/mitochondrial content normalized, relative $\Delta\Psi m$ (r$\Delta\Psi m$). Scale bar 5μm.
(B) Scatter plot of Kc167 cells stained with propidium iodide (PI) as a marker for cell viability (membrane integrity). SSC-A is the flow cytometer side scatter value. The main population (Gate 1) with mainly PI negative cells is used for all analyses.
(C) Reproducibility of CoSRA based r$\Delta\Psi m$ analysis as measured by JC-1 staining in Kc167 cells. Data shown is from five biological replicates, each with >100 000 cells.
(D) Analysis of JC-1 staining kinetics in Kc167 cells of different sizes. The cells were stained with JC-1 and signal intensity was measured over a 30 min time period. Change in JC-1 aggregate/monomer ratio (r$\Delta\Psi m$) was then analyzed separately for small cells, average sized cells and large cells, as indicated on the left panel. The right panel displays the staining kinetics. Light grey shading (right panel) indicates one standard deviation of the mean JC-1 ratio for the average sized cells.
(E) Alternative analysis of JC-1 staining kinetics in Kc167 cells. Cell size scaling of r$\Delta\Psi m$ is shown after different JC-1 staining times (20-60 min). Note that while JC-1 dye does not reach equilibrium, this does not affect the observed cell size scaling of r$\Delta\Psi m$.
(F) Scatter plot of JC-1monomer and aggregate as a function of cell size in Kc167 cells. Note that the $\Delta\Psi m$ dependent aggregate form displays much more variability than the mitochondrial mass dependent monomer form.
(G) Scatter plot of JC-1 ratio (r$\Delta\Psi m$) as a function of JC-1 monomer (mitochondrial content) in Jurkat cells.
(H) CoSRA style analysis of Jurkat cell r$\Delta\Psi m$, where data is displayed as a function of mitochondrial content (JC-1 monomer) instead of cell size. Due to the high correlation of JC1 monomer with cell size (see panel J), the median behavior of r$\Delta\Psi m$ is similar to that seen when analyzing r$\Delta\Psi m$ as a function of cell size.
(I) Maximum intensity projection of a Jurkat cell stained with JC-1 and imaged using OMX structural illumination microscopy. Similar to Kc167 cells (see panel A), the monomer form (green) of the dye localizes to mitochondria and in the presence of $\Delta\Psi m$ forms aggregates (red). Scale bar 5μm.

(J) CoSRA analysis based correlation between JC-1 monomer signal and DDAO-SE based cellular protein staining in Jurkat cells. The near perfect Pearson correlation coefficient (R^2) validates that mitochondrial content scales linearly with cellular protein content, as seen in Kc167 cells (Figure 1B, inset).

(K) Cell size scaling of rΔΨm in Jurkat cells. ΔΨm was measured using TMRE and the signal was normalized to DDAO-SE based cellular protein content staining to obtain rΔΨm values.

(L) Cell size scaling of rΔΨm in various cell lines and primary cells. All cells were stained with JC-1 for 45 min either in the presence or absence of CCCP, except the rat primary hepatocytes, where valinomycin was used instead of CCCP. Note that all cell types, despite being vastly different sized, display similar rΔΨm scaling, where rΔΨm is low in the very smallest cells, peaks in cells which are smaller than average population size and then steadily declines towards larger cell size.

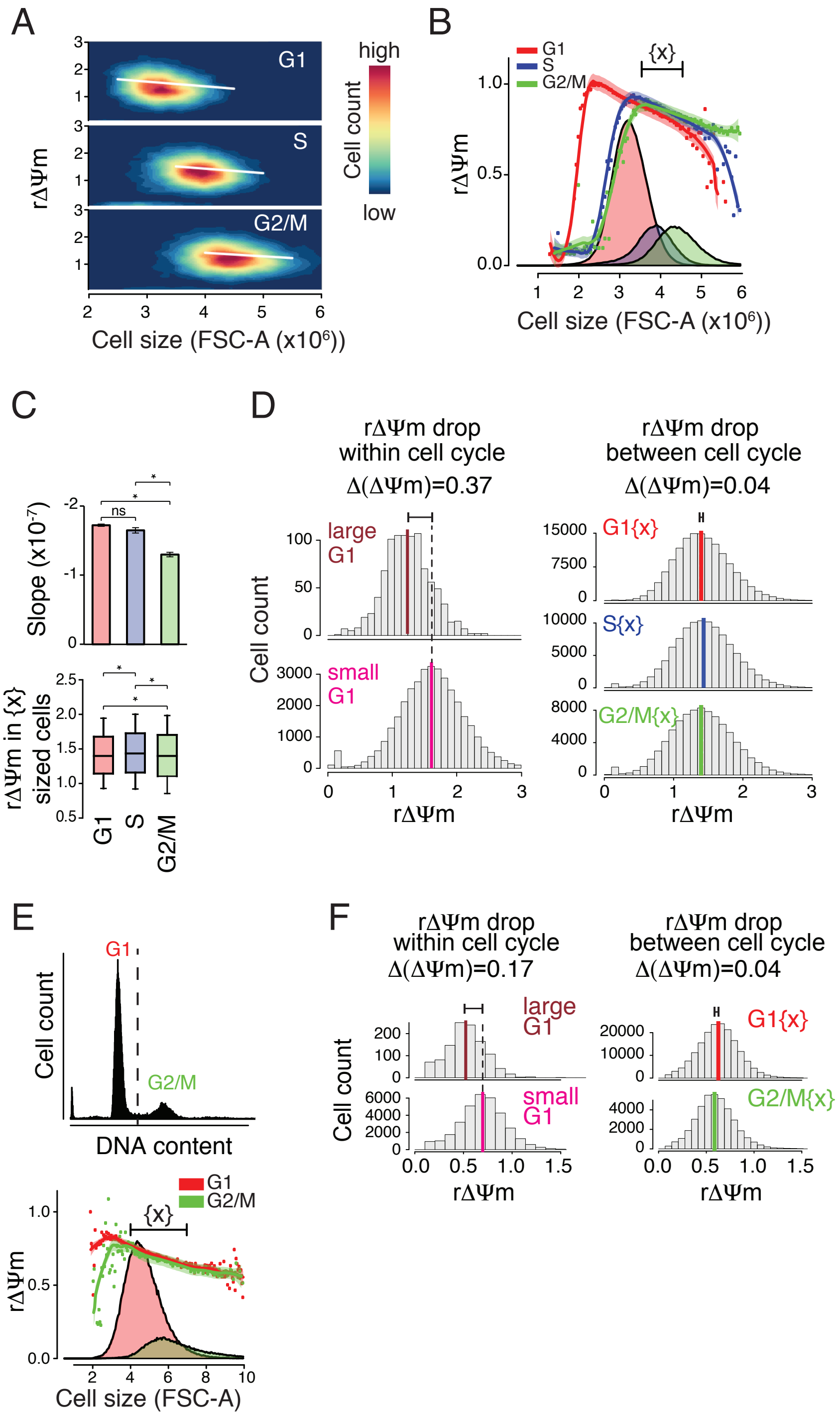

Figure S3. Cell size scaling of rΔΨm in different cell cycle phases, related to Figure 2.
(A) Jurkat cells were stained with JC-1 and Nuclear-ID Red DNA stain. G1, S and G2/M cells were separated based on DNA content and rΔΨm was plotted as a function of cell size. Linear regression curve (white line) was fitted excluding the smallest and largest cells (see experimental procedures for more details). Data is from 8.9x10[5] cells.

(B) CoSRA based analysis of rΔΨm as a function of cell size shown separately for each cell cycle phase. Cell size histograms of G1, S and G2/M cells are shown for reference. {x} displays the cell size area from which rΔΨm values were quantified. Same data as in (A).
(C) *Top,* Quantifications of the linear regression curve slopes in panel (A). Data is mean ± SEM. *Bottom,* quantifications of rΔΨm in cells of the same size, but different cell cycle phase (the cell size range {x} in panel (B)) ($n = 9x10^4$ cells). Note that while both quantifications provide statistically significant results, the extent of the actual changes is minimal. p-values were calculated with ANOVA & Tukey test; * depicts $p < 0.001$.
(D) Quantifications of the rΔΨm change observed in different sized cells within a single cell cycle phase (left) and of the rΔΨm change observed in same sized cells that are in different cell cycle phases (right). Only the cells in range {x} in panel (B) were used ($n = 9x10^4$ cells).
(E) *Top,* DNA content histogram of HUVEC cells stained with propidium iodide. *Bottom,* same as figure (B), but data from HUVEC cells ($n = 3.0x10^5$). Note that the low number of S phase cells precluded reliable analysis from the DNA synthesis phase.
(F) Same as figure (D), but data from HUVEC cells in figure (E).

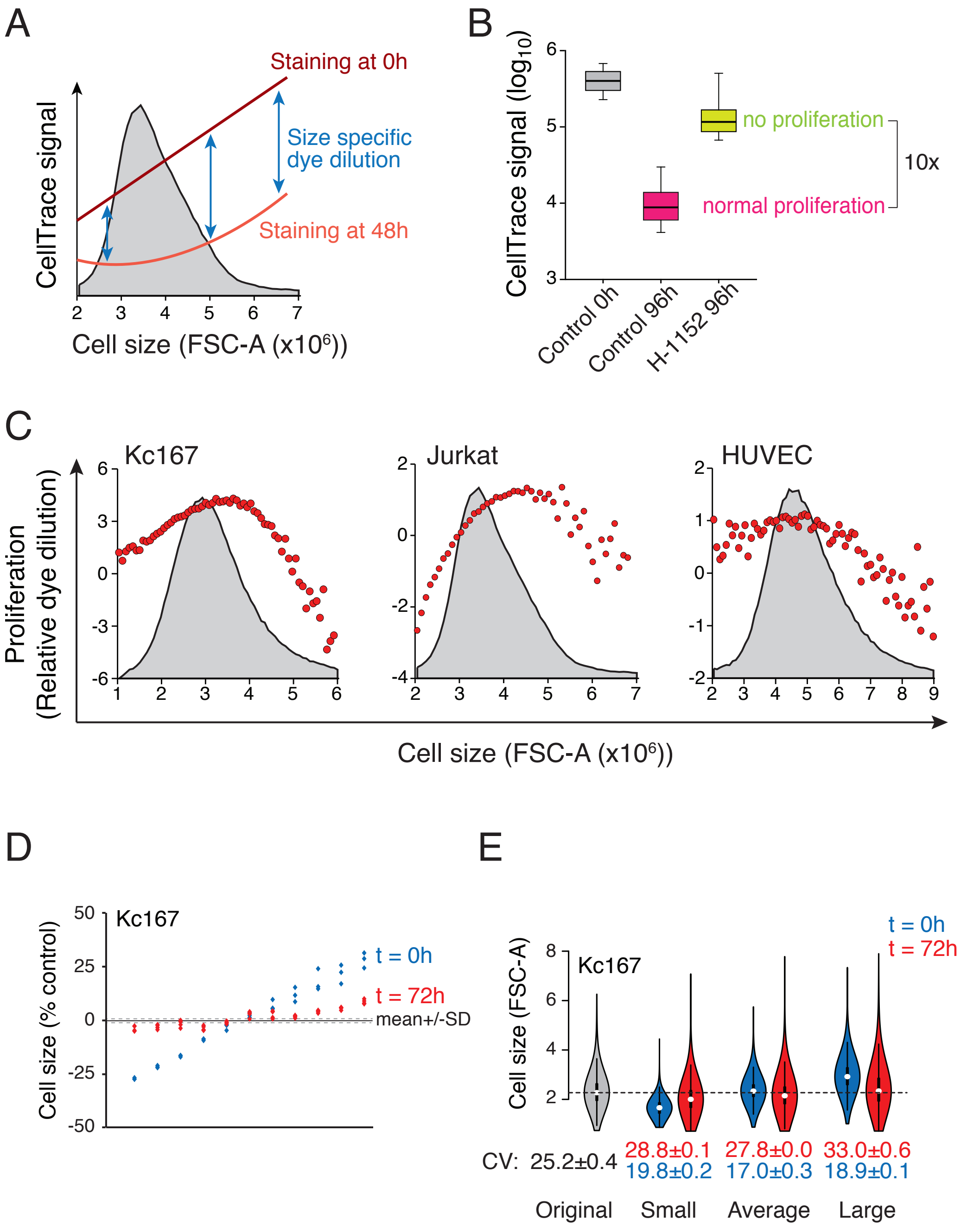

Figure S4. Analysis of optimal cell size by dye dilution and elutriation, related to Figures 3 and 4.

(A) A schematic presentation of how CoSRA approach was used to analyse proliferation. Cells were stained with DDAO-SE, analysed for baseline staining at 0h, grown for 48h and reanalyzed. The relative difference between staining at 0h and at 48h was used as a marker for proliferation.

(B) The suitability of DDAO-SE staining for dye dilution analyses was tested in Jurkat cells by blocking cytokinesis with 2 μM H-1152, a Rho-kinase inhibitor, for 96 h. In the absence of cell division the dye dilution was reduced approximately 10 fold, indicating that the DDAO-SE signal reduction is mainly due to cell proliferation.

(C) Dye dilution based analysis of proliferation in different sized Kc167, Jurkat and HUVEC cells. In all cell types, the proliferative fitness is highest in intermediate sized cells. For all cell types dye dilution was analyzed 48 h after initial staining from > $2x10^5$ cells.

(D) Kc167 cells aim to maintain optimal cell size. Kc167 cells were separated in to size-based subpopulations using centrifugal elutriation and then cultured under normal conditions in order to allow the cells to optimise their size. The mean cell size in each subpopulation immediately after elutriation (0 h, blue) and after 72 h in culture (red) is shown. The data is shown as a relative change in comparison to the unsorted original population (n = 3 in each subpopulation).

(E) Size distributions of the original Kc167 cell population as well as smallest, average sized and largest subpopulations after 0 h (blue) and 72 h (red). Coefficient of variation (CV) is shown below the size distribution plots. Data shown is mean ± SD (n = 3) from 6-$12x10^3$ cells per sample.

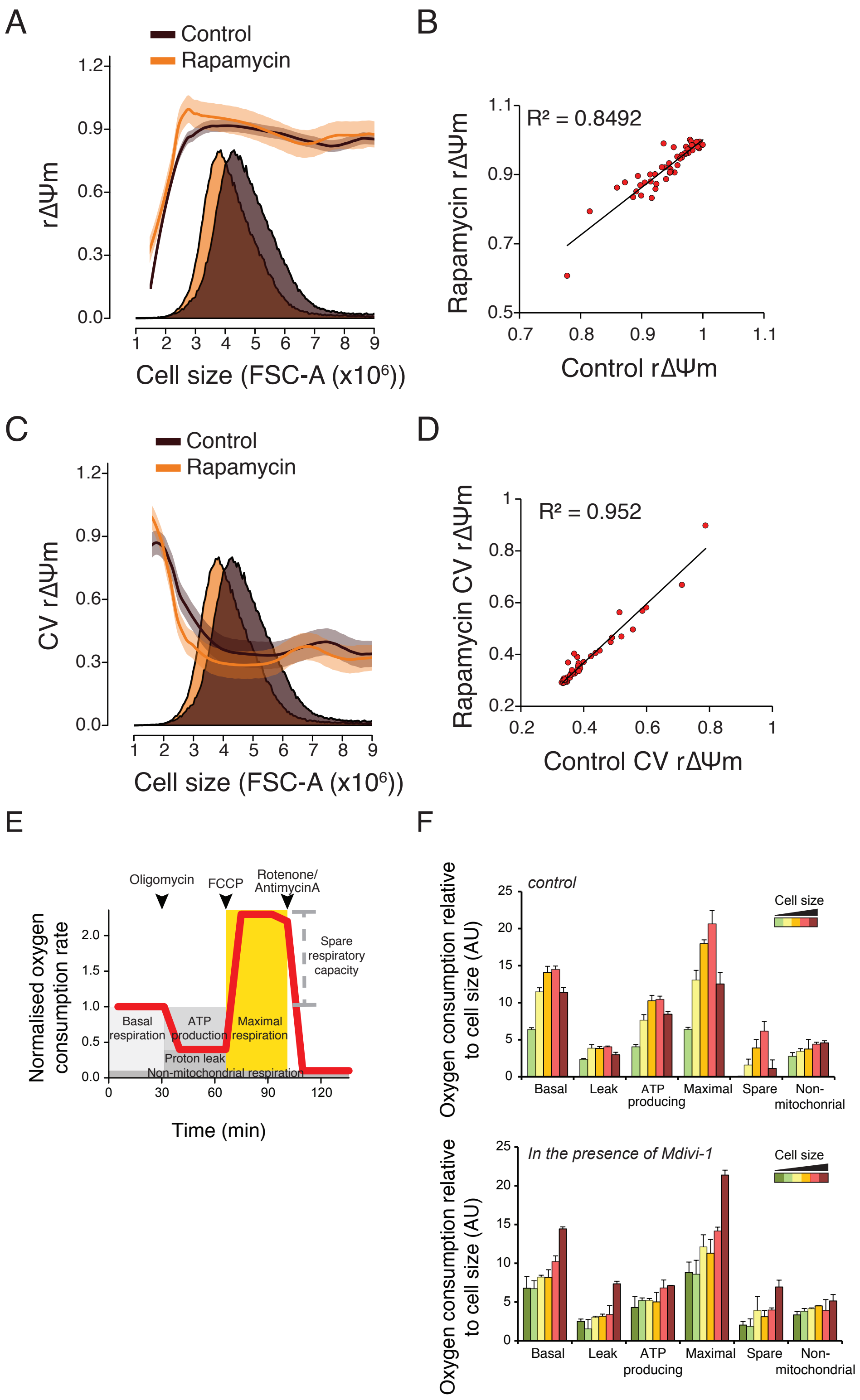

Figure S5. Mitochondrial dynamics and not mTOR regulate cell size scaling of mitochondrial functions , related to Figure 5.

(A) Cell size scaling of rΔΨm in Jurkat cells treated with control (MeOH) or 250 nM rapamycin for 18 h.

(B) Cell size scaling of the cell-to-cell variation in rΔΨm in Jurkat cells treated with control (MeOH) or 250 nM rapamycin for 18 h.

(C) Correlation plot of median rΔΨm values in each cell size bin of samples shown in panel (A). Data is normalised to the average cell size change caused by rapamycin. Pearson correlation coefficient R^2 is also shown.

(D) Correlation plot of rΔΨm variabilities in each cell size bin of samples shown in panel (B). Data is normalised to the average cell size change caused by rapamycin. Pearson correlation coefficient R^2 is also shown. In panels A-D n > $1.7x10^5$ cells.

(E) A schematic illustrating how various aspects of oxygen consumption are measured using successive additions of mitochondria perturbing chemicals (arrows) with the Seahorse Flux Analyser.

(F) Oxygen consumption parameters in different sized Kc167 subpopulations separated by centrifugal elutriation. Top panel displays data from control cells and bottom panel displays data from cells treated with 50μM Mdivi-1 for 24h. Same data as in Figures 1E and 5E.

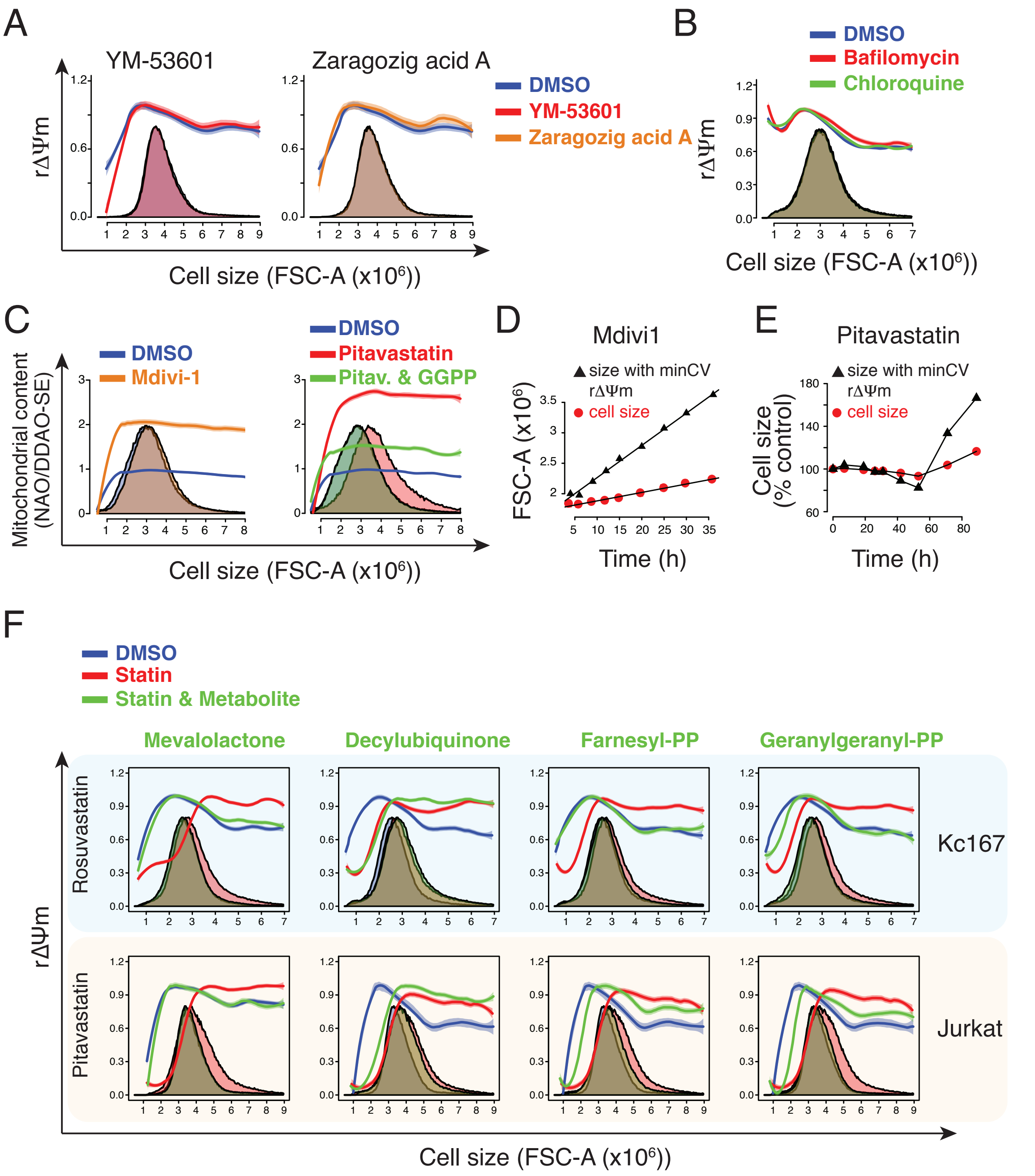

Figure S6. Cell size scaling of rΔΨm requires the mevalonate pathway and cannot be explained by increased mitophagy in larger cells, related to Figure 6.

(A) Cholesterol synthesis inhibitors YM-53601 and Zaragozig acid A do not affect the cell size scaling of rΔΨm in Jurkat cells. Cholesterol synthesis inhibitors were used at 8 μM (YM-53601) and 20 μM (Zaragozig acid A) final concentrations for 72 h.

(B) Autophagy/mitophagy inhibitors do not affect the cell size scaling of r$\Delta\Psi$m in Jurkat cells. Bafilomycin was used at 50 nM concentration and chloroquine at 60 μM for 24 h.

(C) *Left,* Cell size scaling of mitochondrial content in Kc167 cells after 24 h treatment with control (DMSO) or 50 μM Mdivi-1. *Right,* Cell size scaling of mitochondrial content in Kc167 cells after 72 h treatment with control (DMSO), 5 μM pitavastatin or pitavastatin together with geranylgeranylpyrophosphate (GGPP). Note that apart from the very smallest cells both Mdivi-1 and Pitavastatin increase the relative mitochondrial content uniformly in all cell sizes. Mitochondrial content was analyzed using NAO based mitochondrial staining that was normalized to cellular protein content, as measured by DDAO-SE staining. n > 1.0×10^5 cells in all samples.

(D) Median cell size and the size with minimum variability of mitochondrial membrane potential (min CV of r$\Delta\Psi$m) at various time points after addition of 50 μM Mdivi1 in Kc167 cells.

(E) Median cell size and the size with minimum variability of mitochondrial membrane potential (min CV of r$\Delta\Psi$m) at various time points after addition of 5 μM Pitavastatin in Jurkat cells.

(F) Metabolite rescues of statin induced changes in the cell size scaling of r$\Delta\Psi$m. Statin effects in both Kc167 and Jurkat cells were rescued by mevalolactone (which is converted in to mevalonate) and geranylgeranyl-pyrophosphate (GGPP), but not decylubiquinone. These results indicate that protein prenylation regulates the cell size scaling of r$\Delta\Psi$m. Pitavastatin was used at 5 μM concentration and rosuvastatin at 40 μM concentration. n > 1.0×10^5 cells in all samples.

Note that several other cellular perturbations, including mtUPR induction and reactive oxygen species, did not display major changes in the cell size scaling of r$\Delta\Psi$m (data not shown).

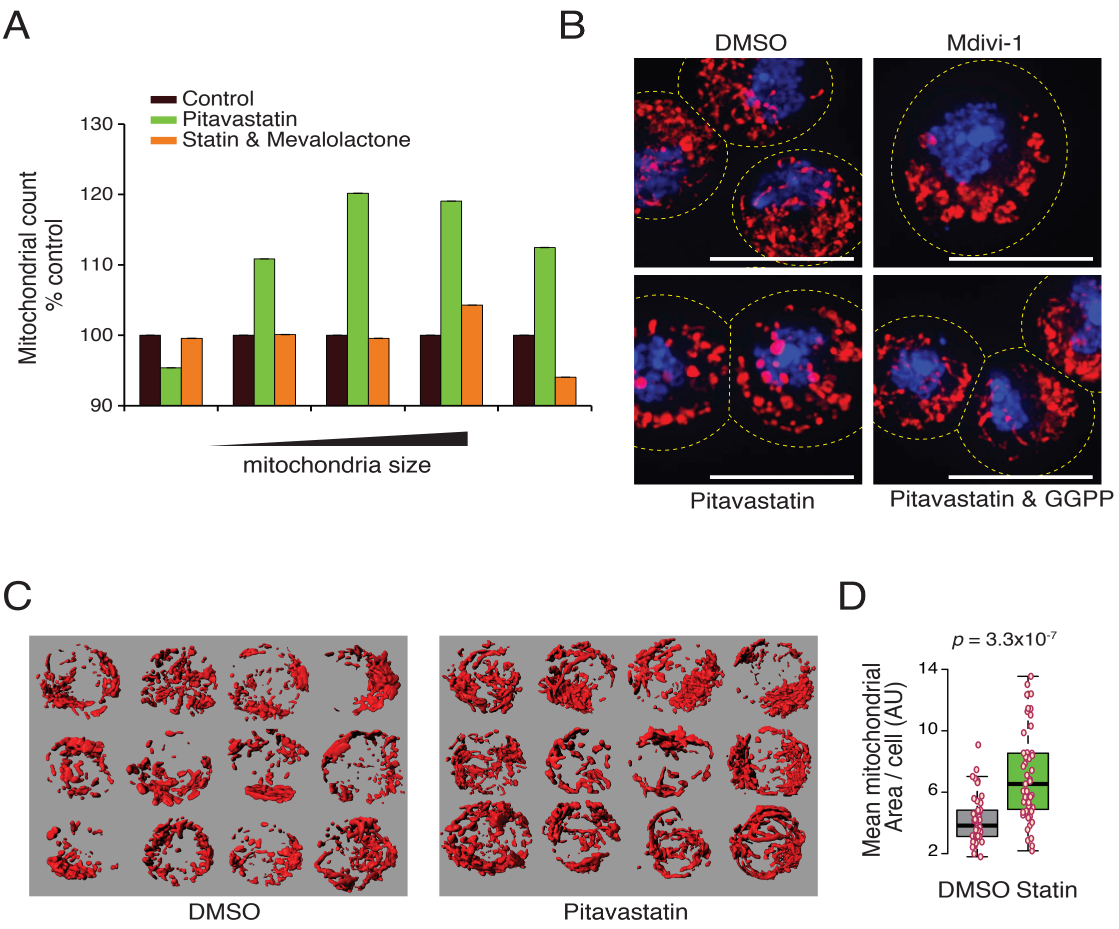

Figure S7. Mitochondrial size and morphology homeostasis require the mevalonate pathway activity, related to Figure 7.

(A) Analysis of mitochondrial sizes in Kc167 cells by flow cytometry. Pitavastatin treatment was rescued by mevalonate (supplied as mevalolactone). Mitochondrial size analysis data represents mean ± SD (n = 3 technical replicates).

(B) Representative maximum-intensity projections of Kc167 cells stained with DAPI (nucleus, blue) and MitoTracker Red (mitochondria, red) after treatment with indicated chemicals. Statin treatment was 72 h and Mdivi-1 treatment was 24 h. Note that Kc167 cells do not form long, elongated mitochondria and highly connected mitochondrial network even in the presence of Drp1 inhibition. Nonetheless, mitochondrial size increases, when mitochondrial division is inhibited. Scalebar 10 μm.

(C) Representative 3D surface projections of mitochondrial network of live Jurkat cells after 72 h treatment with pitavastatin. Cells were stained with MitoTracker Green.

(D) Quantification of mean mitochondrial surface area in each Jurkat cell from samples shown in panel (D) (n = 48 (DMSO) and 51 (Pitavastatin)).

SUPPLEMENTAL EXPERIMENTAL PROCEDURES

Transfections

Kc167 dsRNA transfections were carried out as before (Bjorklund et al., 2006; Miettinen et al., 2014). To construct the long dsRNAs used for RNAi, the following primers were used: taatacgactcactatagggCTCATGATAGCTGAACGCCA and taatacgactcactatagggGTCCGTTTTCAGCAGACTGG for Drp1 as well as taatacgactcactatagggTGAGCAAATACCCCCAAAAG and taatacgactcactatagggGATCTGGAGCGGTGATTTGT for Marf (mitofusin) knockdown.

Jurkat siRNA transfections were carried out using two independent 40nM siRNAs (IDT DNA) for each target and the TransIT-TKO transfection reagent (Mirus Bio) according to manufacturer's instructions. MFN2 was knocked down with two pairs of siRNAs GCAUGGUACCAAGGAGUUAAGUUGA (sense) / UCAACUUAACUCCUUGGUACCAUGCUG (antisense) and GGUUUACUGCGAGGAAAUGCGUGAA (sense) / UUCACGCAUUUCCUCGCAGUAAACCUG (antisense). DNM1L (hsDRP1) was knocked down with GGUUUACUGCGAGGAAAUGCGUGAA (sense) / UUCACGCAUUUCCUCGCAGUAAACCUG (antisense) and AGGAUAUUGAGCUUCAAAUCAGAGA (sense) / UCUCUGAUUUGAAGCUCAAUAUCCUUA (antisense) oligonucleotides.

Cellular proteome scaling with cell size

The protein abundances for the NB4 leukemia cells proteome were from a previously published dataset, where unperturbed NB4 cells were separated by cell size using elutriation and analysed by label-free mass spectrometry (Ly et al., 2014). For the analysis of organelle scaling, the measured proteins were classified to subcellular structures based on GeneOntology annotations obtained from Ensembl Genome Browser. For each subcellular structure, data was normalised to the smallest cell size fraction as well as total protein abundance in each fraction to obtain organelle scaling normalised to total protein.

Flow cytometry measurements

Although ratiometric dyes are the most accurate for measurement of membrane potential (Novo et al., 1999), JC-1 as well as non-ratiometric $\Delta\Psi m$ responsive dyes have potential limitations (Perry et al., 2011). We thus qualitatively confirmed the the JC-1 based results using TMRE based staining of $\Delta\Psi m$ and normalization of this signal to total amine reactive cellular protein content using DDAO-SE. Note that DDAO-SE provides a reliable measure of cell size

(Miettinen and Bjorklund, 2015) and an accurate estimate for total cellular protein content (Kafri et al., 2013). Intracellular pH was measured using the ratiometric SNARF-1 dye, plasma membrane potential using DiBAC4(3) and apoptosis using polarity-sensitive annexin-based biosensor (pSIVA). The fluorescent dyes used were (with final concentrations): DDAO-SE (0.5 μM for protein content and 1 μM for dye dilution), DiBAC4(3) (1 μM), JC-1 (2 μM, except 0.5 μM for hepatocytes and 4 μM for HUVECs). MitoTracker Green FM (50 nM, except 200 nM for mitochondria isolation), MitoTracker Red CMXRos (125 nM), NAO (100 nM), Nuclear-ID Red (20 μM), propidum iodide (30 μg/ml), pSIVA-IANBD (diluted as per suppliers instructions), SNARF-1 (5 μM), TMRE (100 nM) and CellMask Deep Red (1:1000 dilution).

All fluorescence intensities, cell counts and sizes were measure using Accuri C6 flow cytometer (Accuri, now Becton-Dickinson) equipped with 488 nm and 640 nm lasers and standard optical filters (FL1 533/30 nm, FL2 585/40 nm, FL3 >670 nm, FL4 675/25 nm). Electrical current exclusion method was performed using CASY TT (Roche) as before (Miettinen et al., 2014). Flow cytometry measurement of cell size is based on Mie theory on light scattering and usually employ the forward scatter (FSC) pulse area (FSC-A). To validate the relationship between our instrument's (Accuri C6) FSC-A values and electronic cell size measurements (Casy TT instrument), we separated multiple cell types by size using elutriation and measured the average sizes of these subpopulations by flow cytometry and electronic volume measurement. With each cell line, we observed near-perfect correlation's with FSC-A and cell diameter (Pearson R^2=0.95-0.99, Figure S1C). The FSC-A and cell diameter measurements correlated well over the whole range of measured cell sizes from yeast to human U2OS cell (R^2=0.99, Figure S1C). Notably, commercially available size calibration beads also displayed strong size correlation (R^2=0.99), but the slope of the regression line was substantially different from cells, precluding bead-based calibration for cell volumes (Figure S1C). Correlations between FSC-A values were best with cell diameter (R^2=0.99) rather than cell area (R^2=0.97) or volume (R^2=0.90) measurements calculated from electronic volume measurements (Figure S1D), consistent with previous analyses using beads (Cookson et al., 2010). In conclusion, cell size measurements using FSC-A values, at least with our flow cytometer, are accurate, but more closely reflect changes in cell diameter than in cell volume. Note that the ratiometric measurement of JC-1 nevertheless normalises the r$\Delta\Psi$m to mitochondrial and cell volume.

Computational cell size relation analysis (CoSRA) of ΔΨm variability and other cellular parameters

The CoSRA approach was used to analyse cell size scaling of several cellular parameters using the same principle as with ΔΨm analysis. For example, we analysed mitochondrial content as a function of total protein. For this, cells were stained with DDAO-SE, the DDAO-SE signal (FL4-A) was fractionated in to bins instead of FSC-A followed by calculation of the median JC-1 monomer signal for each bin. We also validated that the cell size scaling of rΔΨm observed with JC-1 staining is not due to differential staining kinetics or dye diffusion in small and large cells. This was done by analyzing Kc167 cells through the JC-1 dye incubation. The flow cytometer data was then sorted for different sized cells and the rΔΨm of small, medium and large cells was plotted as a function of time using the flow cytometer time stamp for each cell.

CoSRA based approach was also used to investigate the cell size scaling of the cell-to-cell variability in rΔΨm. The analysis was carried out as above with the exception that fluorescence signal variabilities (expressed as coefficient of variation (CV), which is standard deviation divided by the mean) were calculated for each cell size bin instead of the median fluorescence. The dependency of the CV values on then number of cells in each bin was tested by random sampling of the total population in each bin. The CV rΔΨm curve robustly retained the observed shape including the global minima when equal number (200 or more cells) for each bin was sampled and the CV ΔΨm analysed from these (data not shown). Thus, the cell size with minimum variation of CoSRA-rΔΨm signal cannot be simply explained by the number of cells measured.

Analyzing the contribution of cell size and cell cycle to the rΔΨm

When analysing ΔΨm as a function of cell cycle, median JC-1 ratios (rΔΨm) were plotted by fractionating the population based on DNA content, which was measured using Nuclear-ID Red staining. Cell cycle phases were gated manually based on the resulting DNA histogram. Kc167 cells could not be analysed in this way due to the lack of a suitable live cell DNA stain. Comparison of rΔΨm behavior as a function of cell size and cell cycle in Figure S4 was done as follows. First, a cell size scale during which the rΔΨm scale in relatively linear fashion in all cell cycle phases was selected and marked as {x}. Second, the rΔΨm was quantified from each cell cycle phase using all the cells within {x}. These quantifications, in which the influence of cell size in minimized, suggested only marginal difference in rΔΨm between different cell cycle phases (see Figure S4C). Third, the extent to which rΔΨm changes within

a single cell cycle phase as cells grow in size (in the size range {x}) was compared to the change in rΔΨm when examining cells of same size {x} that are in different cell cycle phases. This indicated that the size dependent change in rΔΨm is several fold larger than the change caused by cells being in a different cell cycle phase, as shown in Figures S4D and S4F.

Also the rate of rΔΨm decrease in each cell cycle phase was analyzed. This was carried out by fitting linear regression lines were to the raw data in Figure S4A so that the smallest and the largest cells, in which ΔΨm does not change quasi-linearly with cell size, were excluded. The slopes of these regression lines were then compared, as shown in Figure S4C.

Size separation by centrifugal elutriation

To examine size dependent changes using population based assays, we separated cells into size-based subpopulations using centrifugal elutriation. As centrifugal elutriation separates cell based on their size and sedimentation density (Banfalvi, 2008), and as cell density is relatively constant (Feijo Delgado et al., 2013), elutriation separates cells essentially based on their size. Approximately $3x10^8$ were resuspended in 3 ml of PBS with 1% FBS. Cells were loaded into counterflow centrifugal elutriator (Beckman JE-5.0/JE), equipped with a standard elutriation chamber and a Cole–Parmer MasterFlex Model 900-292 peristaltic pump. The centrifuge was operated at 3000 rpm and the flow rate was initially set to 21 ml/min. After cells were loaded into the elutriation chamber, 50 ml fractions were collected with increasing flow rates. Collected cells were resuspended in culture medium and cell size and counts were analysed using flow cytometry. Mitochondrial mass and membrane potential from elutriated fractions were measured using MitoTracker Green FM and MitoTracker Red CMXRos, respectively. Flow cytometry measured MitoTracker intensities were normalised to mean FSC-A values.

Oxygen consumption measurements

Oxygen consumption was measured using XF24 Extracellular Flux Analyzer (Seahorse Bioscience). The measurement plates were coated with poly-L-lysine and 80 000 Kc167 cells were plated on each well 2h before the analysis. The analysis was carried out in Schneider's cell culture medium with 10% FBS at room temperature. Oxygen consumption was measured every 6 minutes and the following injections were performed after every 4 measurements: (1) 1μM Oligomycin, (2) 1μM FCCP and (3) 2 μM Rotenone and 2 μM Antimycin A. Figure S5E explains the how each oxygen consumption related parameter was obtained. For each replicate the four oxygen consumption measurements between each injection were averaged, after which

the respiratory parameters were calculated. All the results were normalised to the subpopulation cell size (FSC-A). The analysis was also carried out after treating cells with the mitochondrial division inhibitor Mdivi-1 (50 μM) for 24h or Pitavastatin (5 μM) for 72h. Mdivi-1 and pitavastatin were present for the whole duration of the oxygen consumption assay.

Proliferation measurements

For proliferation based fitness measurements, Kc167, Jurkat and HUVEC cells were first separated into size-based subpopulations using centrifugal elutriation. The average cell size in each subpopulation was measured using flow cytometry and equal cell count of each subpopulation was taken for further culture in normal conditions. The final data is presented as relative cell counts after indicated culture time (y-axis) as a function of the initial subpopulation cell size (x-axis). The total cell population histogram is also show for reference. For Kc167 cells the differences in proliferative capacity of different sized cells were highlighted by presenting the data as a box plot.

The effect of mitochondrial membrane potential on cell proliferation was analyzed in Jurkat cells by first using centrifugal elutriation to separate two different sized subpopulations. These cells were then stained with TMRE and cells with high, medium and low r$\Delta\Psi$m (thus taking cell size into account in assessing the $\Delta\Psi$m, see schematic in Figure 2E) were separated using flow sorting (Influx Cell Sorter, Becton Dickinson) and cell sizes were reanalyzed in the separated populations to validate that r$\Delta\Psi$m based separation did not change the average cell size. Equal cell counts of these r$\Delta\Psi$m separated cells were then cultured for 100h in normal growth conditions, after which cells were recounted.

When analyzing cell proliferation using the CoSRA method (see above), cells were first stained using DDAO-SE and the baseline staining levels were measured using flow cytometer. The cells were then cultured normally for 48h after which cells were reanalyzed for DDAO-SE stain dilution (See Figure S5A). The relative dye dilution was calculated for each cell size bin and illustrated as before. If cells of all sizes proliferate at an equal rate, the relative dye dilution should be the same in all cells. High dye dilution indicates faster proliferation (Miettinen and Bjorklund, 2015).

Protein synthesis measurements

Jurkat cells were stained with Click-iT Plus OPP Alexa Fluor 488 Protein Synthesis Assay Kit (LifeTechnologies) and MitoTracker Red CMXRos. OPP and MitoTracker Red were added to

to 20 μM and 200 nM final concentration, respectively and incubated for 30 min. Cells were fixed with 3.7% formaldehyde and processed as instructed in the OPP assay kit. Cells were analysed using flow cytometry.

Western blots

Jurkat siRNA efficiency was validated by western blot. The cells were lysed directly in Laemmli buffer, sonicated and analyzed on a SDS-PAGE gel. The antibodies were obtained from Abcam (DRP1 (ab56788) and MFN2 (ab124773)) and Cell Signaling Technology (GAPDH (#5174)), used at the concentrations recommended by suppliers and detected using infrared-dye conjugated secondary antibodies and LICOR Odyssey detection system.

Isolation of mitochondria

Cells were stained with 200 nM NAO for 15 min and collected by centrifugation. Crude mitochondrial fraction was isolated after lysis in 10 mM Tris–MOPS (pH to 7.4), 1mM EGTA, 0.2M Sucrose using dounce homogeniser. Nuclear pellet was removed by centrifugation at 600xg for 5 min. Mitochondrial fraction was obtained by centrifugation 1t 7000xg for 5 min. The resulting pellet was suspended in PBS and immediately analysed by flow cytometry (Mattiasson, 2004; Petit et al., 1990). Mitochondria were identified by measuring NAO signal (FL1-A) and forward scatter (FSC-A). For mitochondrial size analysis, FSC-A ranges of increasing sizes were selected and the percentage of mitochondria relative to control sample in each size range was calculated.

Microscopy

For JC-1 staining validations, live Kc167 and Jurkat cells were imaged using OMX structured illumination microscope (GE Healthcare) with standard filters (528/48 nm and 609/37 nm filters for JC-1 monomer and aggregate, respectively) and 100x objective. Microscopy slides were coated with 0.1% poly-L-lysine. Cells were imaged in the presence of JC-1 stain in complete media. For examination of mitochondrial morphology, Kc167 and Jurkat cells were treated with indicated chemicals and moved to coverslips coated with 0.1% poly-L-lysine. The cells were stained with MitoTracker Red CMXRos, washed twice with PBS and fixed with 4% paraformaldehyde. Cells were then permeabilized with 0.1% Tween in PBS, washed with TBS

and the nuclei were stained with 4',6-diamidino-2-phenylindole (DAPI) followed by two washes with TBS. Cells were mounted using VectaShield mounting media (VectorLabs).

For microscopic analysis of isolated mitochondria, cells were incubated with 200 nM MitoTracker green for 30 min. Mitochondria were isolated and imaged as shown below. For live cell imaging, Jurkat cells were stained with 50 nM MitoTracker Green and 1:1000 diluted CellMask Deep Red for 15 min in complete medium at 37°C. Cells were centrifuged for 3 min at 500xg and resuspended in fresh complete medium without the dyes but with DMSO or Pitavastatin and added on polylysine coated coverslips for imaging. U2OS cells expressing mCherry-GFP were imaged in the absence of any stains. Mitochondrial fragmentation was induced with 2 μM FCCP for 1 h. Cells were imaged with DeltaVision widefield deconvolution microscope using standard filters (DAPI, and TRITC) and 100X objective.

Image processing

All images were deconvoluted using DeltaVision SoftWoRx software. Image processing was done using ImageJ and Imaris (v. 7.2.1, Bitplane.com). Binary images of mitochondria stained with MitoTracker Red were created using the binary processing tool in ImageJ with default settings for all images. For surface rendering, individual cells were cut out from widefield images and contrast was adjusted to 0.5% saturated pixels per slice in ImageJ. 3D rendering of MitoTracker Green stained mitochondrial network was performed with Imaris software. Surface smoothing was used with 0.5 μm Surfaces Area Detail Level and thresholding adjusted to half-maximal intensity. Mitochondrial size quantifications were exported from Imaris and analysed using R. Maximum intensity projections were created in ImageJ.

SUPPLEMENTAL REFERENCES

Banfalvi, G. (2008). Cell cycle synchronization of animal cells and nuclei by centrifugal elutriation. Nat Protoc *3*, 663-673.

Bjorklund, M., Taipale, M., Varjosalo, M., Saharinen, J., Lahdenpera, J., and Taipale, J. (2006). Identification of pathways regulating cell size and cell-cycle progression by RNAi. Nature *439*, 1009-1013.

Cookson, N.A., Cookson, S.W., Tsimring, L.S., and Hasty, J. (2010). Cell cycle-dependent variations in protein concentration. Nucleic Acids Res *38*, 2676-2681.

Feijo Delgado, F., Cermak, N., Hecht, V.C., Son, S., Li, Y., Knudsen, S.M., Olcum, S., Higgins, J.M., Chen, J., Grover, W.H.*, et al.* (2013). Intracellular water exchange for measuring the dry mass, water mass and changes in chemical composition of living cells. PLoS One *8*, e67590.

Kafri, R., Levy, J., Ginzberg, M.B., Oh, S., Lahav, G., and Kirschner, M.W. (2013). Dynamics extracted from fixed cells reveal feedback linking cell growth to cell cycle. Nature *494*, 480-483.

Ly, T., Ahmad, Y., Shlien, A., Soroka, D., Mills, A., Emanuele, M.J., Stratton, M.R., and Lamond, A.I. (2014). A proteomic chronology of gene expression through the cell cycle in human myeloid leukemia cells. Elife *3*, e01630.

Mattiasson, G. (2004). Flow cytometric analysis of isolated liver mitochondria to detect changes relevant to cell death. Cytometry A *60*, 145-154.

Miettinen, T.P., and Bjorklund, M. (2015). Mevalonate Pathway Regulates Cell Size Homeostasis and Proteostasis through Autophagy. Cell Rep *13*, 2610-2620.

Miettinen, T.P., Pessa, H.K., Caldez, M.J., Fuhrer, T., Diril, M.K., Sauer, U., Kaldis, P., and Bjorklund, M. (2014). Identification of transcriptional and metabolic programs related to mammalian cell size. Curr Biol *24*, 598-608.

Novo, D., Perlmutter, N.G., Hunt, R.H., and Shapiro, H.M. (1999). Accurate flow cytometric membrane potential measurement in bacteria using diethyloxacarbocyanine and a ratiometric technique. Cytometry *35*, 55-63.

Perry, S.W., Norman, J.P., Barbieri, J., Brown, E.B., and Gelbard, H.A. (2011). Mitochondrial membrane potential probes and the proton gradient: a practical usage guide. Biotechniques *50*, 98-115.

Petit, P.X., O'Connor, J.E., Grunwald, D., and Brown, S.C. (1990). Analysis of the membrane potential of rat- and mouse-liver mitochondria by flow cytometry and possible applications. Eur J Biochem *194*, 389-397.
