# Supplementary material for: Cellular Allometry of Mitochondrial Functionality Establishes the Optimal Cell Size
Source: Dev Cell. 2016 Nov 7;39(3):370–82. doi: 10.1016/j.devcel.2016.09.004 (PMC5104693; doi:10.1016/j.devcel.2016.09.004)
Supplement: Document S2. Article plus Supplemental Information [file mmc2.pdf]

Article

Developmental Cell

Cellular Allometry of Mitochondrial Functionality Establishes the Optimal Cell Size

Graphical Abstract

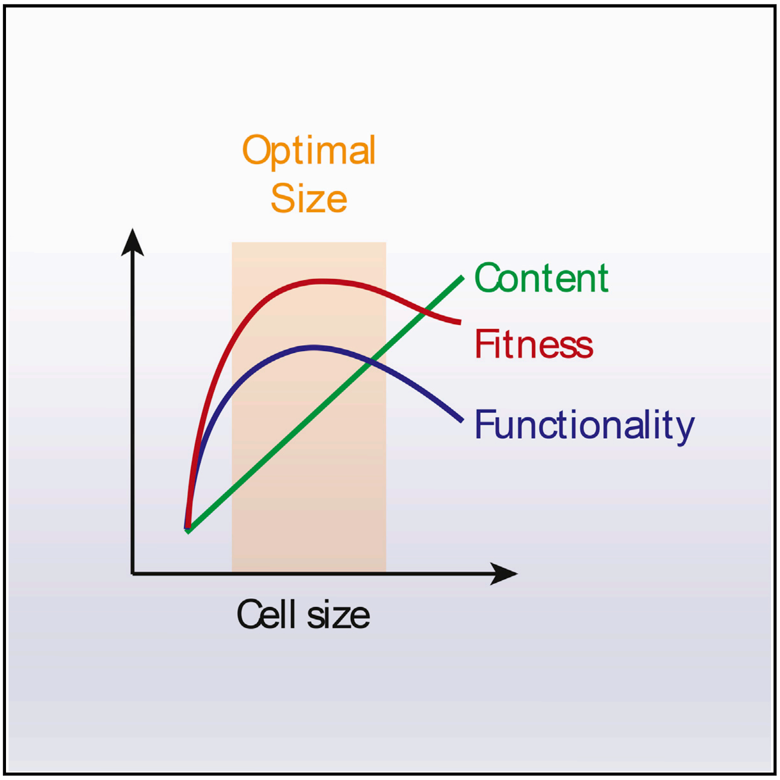

Authors

Teemu P. Miettinen, Mikael Björklund

Correspondence

mikael.bjorklund.lab@gmail.com

In Brief

Organelle content scales linearly with cell size. Miettinen and Björklund investigate how this relates to organelle function and show that mitochondrial functionality and cellular fitness are highest at intermediate cell sizes, suggesting the existence of an optimal cell size. The mevalonate pathway contributes to cell size scaling of mitochondrial function.

Highlights

- Mitochondrial functionality is highest in intermediate-sized cells in a population
- Mitochondrial membrane potential changes with cell size, not cell cycle
- Evidence for an optimal cell size, whereby functionality and fitness are maximized
- Mitochondrial dynamics and mevalonate pathway required for the optimal cell size

CrossMark
Miettinen & Björklund, 2016, Developmental Cell *39*, 370–382
November 7, 2016 © 2016 The Authors. Published by Elsevier Inc.
http://dx.doi.org/10.1016/j.devcel.2016.09.004

CellPress

Developmental Cell
Article

Cellular Allometry of Mitochondrial Functionality Establishes the Optimal Cell Size

Teemu P. Miettinen[1,2,3] and Mikael Björklund[1,4,*]
[1]Division of Cell and Developmental Biology, School of Life Sciences, University of Dundee, Dundee DD1 5EH, UK
[2]MRC Laboratory for Molecular Cell Biology, University College London, Gower Street, London WC1E 6BT, UK
[3]Koch Institute for Integrative Cancer Research, Massachusetts Institute of Technology, Cambridge, MA 02139, USA
[4]Lead Contact
*Correspondence: mikael.bjorklund.lab@gmail.com
http://dx.doi.org/10.1016/j.devcel.2016.09.004

SUMMARY

Eukaryotic cells attempt to maintain an optimal size, resulting in size homeostasis. While cellular content scales isometrically with cell size, allometric laws indicate that metabolism per mass unit should decline with increasing size. Here we use elutriation and single-cell flow cytometry to analyze mitochondrial scaling with cell size. While mitochondrial content increases linearly, mitochondrial membrane potential and oxidative phosphorylation are highest at intermediate cell sizes. Thus, mitochondrial content and functional scaling are uncoupled. The nonlinearity of mitochondrial functionality is cell size, not cell cycle, dependent, and it results in an optimal cell size whereby cellular fitness and proliferative capacity are maximized. While optimal cell size is controlled by growth factor signaling, its establishment and maintenance requires mitochondrial dynamics, which can be controlled by the mevalonate pathway. Thus, optimization of cellular fitness and functionality through mitochondria can explain the requirement for size control, as well as provide means for its maintenance.

INTRODUCTION

For every cell type there is a typical cell size, possibly reflecting the size for optimal cellular functions (Ginzberg et al., 2015). Smaller size should be more efficient in nutrient uptake and metabolism due to greater surface-to-volume ratio. However, the very smallest cell size may not be ideal for cellular fitness and functionality. What determines the optimal cell size is not known. Cellular protein as well as organelle content typically scales linearly with cell size (Reber and Goehring, 2015; Schmoller and Skotheim, 2015), and it is commonly assumed that functionality increases linearly with total protein and organelle content. For example, the well-established linear mitochondrial content scaling (Kitami et al., 2012; Posakony et al., 1977; Rafelski et al., 2012) is considered a metabolic adaptation to increased cell size, as mitochondria are key organelles for growth and proliferation. However, it is difficult to reconcile how linear size scaling of mitochondrial functionality, which reflects the metabolic activity of the cell, could help in establishing an optimal cell size (Figure 1A). Instead, the presence of cell size-dependent changes in functionality and/or feedback to metabolic and growth processes have been considered critical for cell size homeostasis (Ginzberg et al., 2015; Schmoller and Skotheim, 2015).

Allometry is the study of biological scaling relative to organismal size with a key interest in the relationship between organismal metabolic rate and body mass. Allometric scaling laws were established through measurements of oxygen consumption (reflecting mitochondrial activity) and body mass. These experiments established that metabolic rate increases more slowly than body size. Thus larger organisms have less active oxidative metabolism per unit volume (Glazier, 2005; Kleiber, 1932). While allometry has been extensively studied at the organismal level, cellular allometry of metabolism and mitochondrial activity, i.e., how these functionalities scale with cell size, has not been carefully examined within the same cell population. Such analyses could be important for understanding the regulation of cell growth and cell size from evolutionary, biomedical, and biophysical perspectives.

Previous studies have shown that cellular growth rates are maximized in intermediate-sized phytoplankton species (Maranon, 2015) and that growth rates decline in the largest mammalian cells within a single population (Son et al., 2012; Tzur et al., 2009). These results suggest that cell growth may display nonlinear cellular allometry, whereby metabolic activity is reduced at both ends of the cell size range (Figure S1A). This is in stark contrast to the linear, isometric scaling of cellular protein and organelle content. Here we use both single-cell and population-based analyses to study the cell size scaling of mitochondrial functionality in animal cells. We show that cells within a single population exhibit an optimal cell size, whereby mitochondrial functionality and fitness are maximized.

RESULTS

Mitochondrial Functionality Scales Nonlinearly with Cell Size

We recently reported that the relative expression of many mitochondrial genes declines in cells larger than normal size in cultured *Drosophila* cells and in mouse hepatocytes in vivo, although the mitochondrial content per volume unit did not

 © 2016 The Authors. Published by Elsevier Inc.
This is an open access article under the CC BY license (http://creativecommons.org/licenses/by/4.0/).

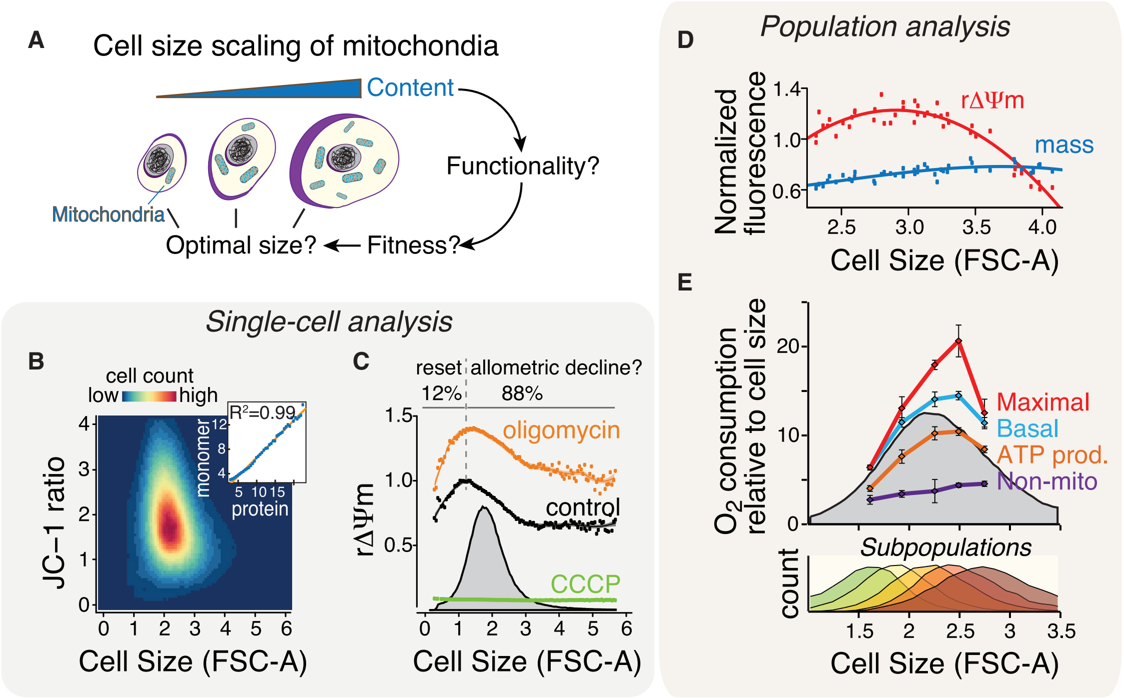

Figure 1. Cell Size Scaling of Mitochondrial Membrane Potential and Respiration Are Nonlinear

(A) Cell size scaling of mitochondrial content is known to be linear, but how this translates to mitochondrial functionality is unknown.

(B) Flow cytometry data of Kc167 cells stained with ratiometric mitochondrial membrane potential (ΔΨm) responsive JC-1 dye and plotted as a function of cell size (forward scatter, FSC-A). Inset shows the correlation between total cellular protein (DDAO-SE) and mitochondrial content (JC-1 monomer).

(C) Computational cell size relation analysis for rΔΨm, the cell size normalized mitochondrial membrane potential. Untreated control cells as well as oligomycin- and CCCP-treated cells were stained with JC-1 and measured with flow cytometry. The cells were computationally fractionated by size and median ΔΨm was calculated for each size fraction (bin). Gray plot shows the size distribution of the cell population. Individual data points display the median rΔΨm for each cell size with line depicting the local polynomial regression curve (typical rΔΨm trajectory with cell size). The percentages of cells smaller ("reset phase") and larger ("allometric decline") in size than those with maximal rΔΨm are shown above the plot. n > 3.5×10^5 cells for each sample.

(D) Population-based analysis of mitochondrial content and rΔΨm with cell size. Kc167 cells were separated by centrifugal elutriation to size-based subpopulations. Mitochondrial content and ΔΨm were measured using MitoTracker green and red, respectively, and normalized to cell size (FSC-A). Three replicates for each subpopulation were measured and data were fitted using a trendline.

(E) Oxygen consumption parameters for elutriated subpopulations (lower panel) were measured using the Seahorse Extracellular Flux analyzer and normalized to mean cell size of each subpopulation. Oxygen consumption data represent mean ± SD (n = 3–4).

See also Figures S1 and S2.

change (Miettinen et al., 2014). Further analysis of proteome data from a leukemia cell line separated by cell size (Ly et al., 2014) showed that cell size scaling of proteins associated with different organelles, including mitochondria, scales isometrically (Figure S1B). As mitochondrial gene expression responds sensitively to functional demands, these data lead us to hypothesize that mitochondrial functional scaling could differ from the mitochondrial content scaling with cell size.

We wanted to analyze cell size scaling of mitochondrial functionality in a steady state, unperturbed cell population, where cell size differences reflect growth observed during the cell cycle. To achieve this aim, we used flow cytometry-based single-cell measurements together with JC-1 dye, which as a ratiometric reporter provides cell size normalized (relative) mitochondrial membrane potential (rΔΨm) measurements (see Experimental Procedures). In a steady state ΔΨm reflects the balance between the rate of electron transport and the rate of ATP utilization, thus providing a convenient measure for mitochondrial functionality. We also validated that the forward scatter (FSC-A) values provided by flow cytometry are accurate measurements of cell size (Figures S1C and S1B). The flow cytometry data consisting of cell size measurements (FSC-A) and rΔΨm are computationally fractionated into size-based subpopulations (bins) for which median rΔΨm is calculated and used to fit a local polynomial regression curve (loess) that visualizes the typical rΔΨm trajectory with cell size (Figure S2; see also Experimental Procedures). In our approach, each cell size bin corresponds to approximately 100 nm in cell diameter based on calibration data. To overcome the high cell-to-cell variability in ΔΨm (Figures 1B and S2), we used 10^5–10^6 cells for typical analysis.

Consistent with previous reports (Kitami et al., 2012; Posakony et al., 1977; Rafelski et al., 2012), mitochondrial mass (as indicated by the green JC-1 dye monomer fluorescence) increased linearly with *Drosophila* Kc167 cell size (Pearson correlation R^2 = 0.99; Figures 1B [inset], S2F). However, rΔΨm displayed a sharp increase in the smallest cells followed by a slower decline toward larger cells (Figure 1C). Similar cell size scaling of rΔΨm was

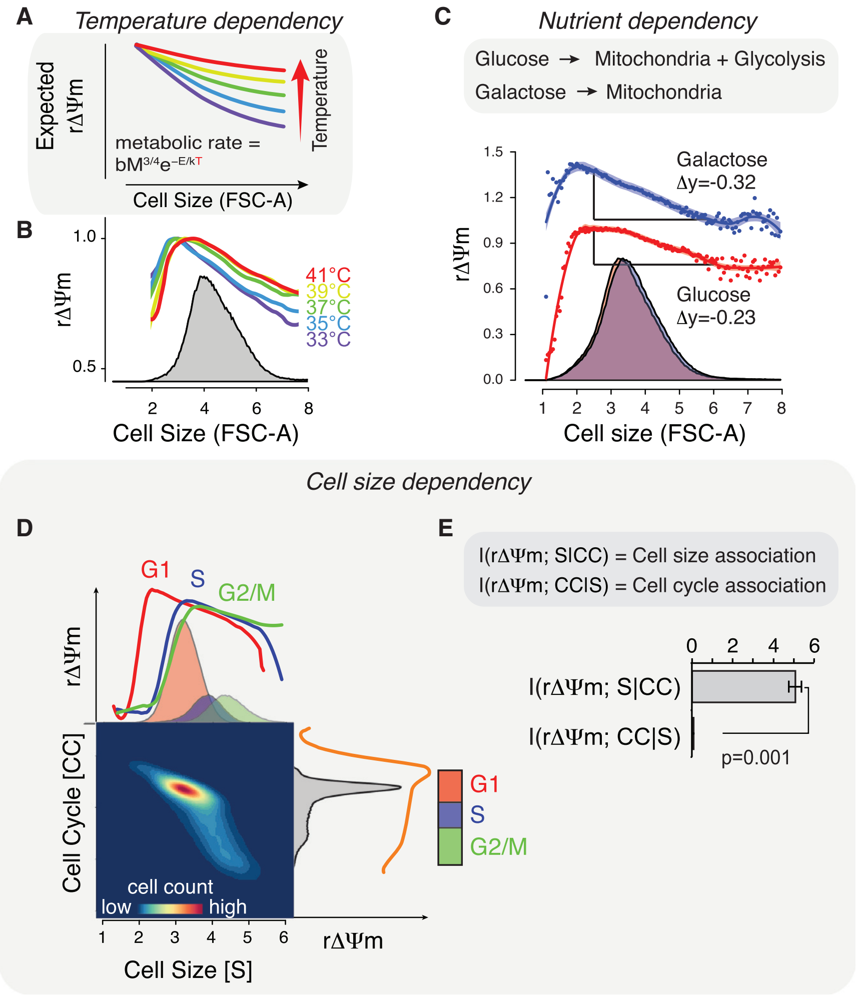

Figure 2. Cell Size Scaling of rΔΨm Is Affected by Temperature and Cellular Metabolism, but Not Cell Cycle

(A) Expected effect of temperature on cell size scaling of rΔΨm. Metabolic theory predicts that metabolic rate changes not only with size but also with temperature (Gillooly et al., 2001), as indicated by the equation. b is a general scaling constant, M is mass, T is temperature, k is Boltzmann's constant, and E is the activation energy of metabolism. Thus, increased temperature is expected to cause a shallower decline in rΔΨm.

(B) Jurkat cells were incubated in complete medium at different temperatures for 2 hr to adjust the metabolic rate followed by JC-1 staining-based analysis. Note that rΔΨm scaling does not change further at the highest temperatures as cells approach denaturating temperatures. $n > 1 \times 10^5$ for each temperature point.

(C) Effect of mitochondrial nutrient source on cell size scaling of rΔΨm. Glucose-grown cells utilize both glycolysis and mitochondria for energy generation. Galactose-grown cells are reliant on mitochondrial metabolism. Jurkat cells were cultured on glucose-containing medium or adapted to grow on galactose followed by rΔΨm measurement. Δy values show the magnitude of rΔΨm decline in the linear range of the curve.

(D) ΔΨm associates with cell size but not cell cycle. Jurkat cells were stained with JC-1 and NuclearRed ID DNA dye to visualize rΔΨm scaling with cell size in each cell-cycle phase (top left) and with DNA content (bottom right).

(E) Quantification of conditional information analysis of ΔΨm association with cell size and cell cycle showing the strength of ΔΨm association with cell size (S) and cell cycle (CC) from Jurkat cells measured as shown in (D). Data shown represent mean ± SD (n = 3) with $>7.5 \times 10^5$ cells per replicate.

See also Figure S3.

observed in primary (e.g., human umbilical vein endothelial cells [HUVECs]) and immortalized (e.g., Jurkat) cell types from various species and also when using tetramethylrhodamine ethyl ester (TMRE), another ΔΨm-responsive dye (Figures S2L, and S1E). The nonlinear scaling pattern of rΔΨm persisted when mitochondria were further polarized by blocking ATP synthase, and was lost when mitochondria were uncoupled (Figure 1C). Plasma membrane potential did not display similar scaling with cell size (Figure S1G).

We next examined the cell size scaling of mitochondrial functions by first separating Kc167 cells into size-based subpopulations using centrifugal elutriation. Mitochondrial mass, as measured by MitoTracker green dye, remained constant in different-sized cells after normalization to cell size. In contrast, the ΔΨm-dependent fluorescence of MitoTracker red dye displayed a substantial decrease in both the smallest and largest cells (Figure 1D). Consistent with the MitoTracker red data, direct analysis of oxygen consumption indicated that various parameters of mitochondrial respiration displayed a nonlinear cell size scaling, whereby mitochondrial respiration is highest in intermediate-sized cells (Figure 1E). These single-cell and population-level data indicate that cell size scaling of mitochondrial content and functionality are distinct from each other, as mitochondrial functionality is maximized in intermediate-sized cells.

Scaling of Mitochondrial Membrane Potential Is Cell Size, Not Cell Cycle, Dependent

We reasoned that if the rΔΨm scaling is indeed cell size dependent and related to the allometric decline in metabolic rate, we should also observe temperature dependency as predicted by the Arrhenius equation (Gillooly et al., 2001) (Figure 2A). We measured the cell size scaling of rΔΨm at temperatures ranging from 33°C to 41°C in Jurkat cells and observed a stronger decline in rΔΨm scaling with lower temperatures, a result consistent with the theory (Figure 2B). In addition, *Drosophila* cells, which are cultured at 23.5°C, display a stronger decline in rΔΨm toward larger cells than Jurkat cells, which are cultured at 37°C (compare Figures 1C and 2B).

We next examined the nutrient dependency of rΔΨm scaling. We cultured Jurkat cells in galactose-containing medium to increase their dependency on mitochondrial activity. In comparison with Jurkat cells grown in glucose-containing medium, galactose-grown cells displayed higher rΔΨm in smaller cells

and a slightly stronger decline in r$\Delta\Psi$m in larger cells (Figure 2C). These data suggests that the observed mitochondrial functional scaling is due to the mitochondria in smallest and largest cells being less active than their mid-sized counterparts, although mitochondria in all sizes of cells remain functional (see also Figure 1C). Thus, the r$\Delta\Psi$m decrease in larger cells could be explained by reduced metabolic activity caused by the increase in cell size, possibly through changes in surface-to-volume ratio. The low r$\Delta\Psi$m in the very smallest cells may be a "newborn effect," whereby the small daughter cells are yet to reset their inherited low metabolic activity after mitosis.

As cells grow larger within the cell cycle, we next examined whether the $\Delta\Psi$m changes are size specific or whether they could be better explained by the cell cycle. Analysis of human Jurkat cells and HUVECs stained for r$\Delta\Psi$m and DNA content indicated that r$\Delta\Psi$m declines also with the cell cycle, as expected. However, analysis of r$\Delta\Psi$m as a function of cell size separately in G_1, S, and G_2/M phases of the cell cycle revealed that r$\Delta\Psi$m scaling with cell size behaves similarly regardless of the cell-cycle phase (Figures 2D, S3B, and S3E). Comparison of the size-dependent change in r$\Delta\Psi$m within a single cell-cycle phase with the cell-cycle-dependent change in cells of the same size indicated that the r$\Delta\Psi$m change is 5–10 times greater with cell size than between the cell-cycle phases in Jurkat cells and HUVECs (Figures S3D and S3F). We also used Shannon's information theory (conditional mutual information) (Shannon, 1948) to quantify the association of $\Delta\Psi$m with cell size = S, when cell cycle is fixed, i.e., I($\Delta\Psi$m, S|CC) and with cell cycle = CC, when cell size is fixed, i.e., I($\Delta\Psi$m, CC|S). r$\Delta\Psi$m displayed $\sim$40 times stronger association with cell size than the cell cycle in Jurkat cells ($p = 0.001$, t test) (Figure 2E). In conclusion, r$\Delta\Psi$m is dependent on cell size, not the cell cycle, as could be predicted from allometric laws (Glazier, 2005; Kleiber, 1932; Kozlowski et al., 2003).

Cell Size Scaling of Mitochondrial Functions Sets the Optimal Cell Size

We next asked what are the consequences resulting from the uncoupling of mitochondrial content and function. Apoptosis and heterogeneity are determinants of mitochondrial and cellular fitness. We therefore examined whether metabolic heterogeneity (measured as coefficient of variability of $\Delta\Psi$m) is minimized at any particular cell size and whether this is related to cellular fitness. Minimum r$\Delta\Psi$m variability was found to be associated with the median cell size in the Kc167 population using both JC-1 and TMRE dyes (Figures 3A and S1E). Similarly, there was a strong correlation between minimum variability and median cell size across primary and immortalized cell types from multiple organisms (Figure 3B). Measurement of apoptosis using phosphatidylserine exposure indicated that the number of dying cells increases especially in the smallest but also in the largest Kc167 cells (Figure 3C). These cells were early apoptotic cells, as our experiments exclude the cell population with compromised plasma membrane integrity (see Experimental Procedures). Furthermore, the cell size dependency pattern of apoptosis strongly correlated with the $\Delta\Psi$m variability (R^2 = 0.94, Figure 3C inset), supporting the concept that a low $\Delta\Psi$m variability is beneficial for cellular fitness. These data, together with the cell size scaling of oxygen consumption, suggest that mitochondrial functions and fitness are optimized in intermediate-sized cells within a single population.

Cellular fitness is commonly evaluated by measuring cell proliferation, which for cells in nutrient-rich culture conditions can also be considered as a measure of the growth rate (production of cellular biomass). We separated Kc167 cells, HUVECs, and Jurkat cells into subpopulations with different cell sizes and thereafter allowed them to proliferate in complete culture medium. As with mitochondrial respiration (see Figure 1E), the average-sized cell populations appeared optimal, resulting in the highest number of cells (Figure 3D). Similar results were obtained when cells of different sizes were not physically separated but proliferation was analyzed using fluorescent dye dilution upon cell division (Miettinen and Bjorklund, 2015) (Figures S4A–S4C). To analyze the role of mitochondria more directly, we sorted Jurkat cells first by size using elutriation and then by r$\Delta\Psi$m using fluorescence-activated cell sorting, to obtain populations with low, medium, and high r$\Delta\Psi$m (Figure 3E). Within the same-sized cells, medium and high r$\Delta\Psi$m cells were more proliferative than low r$\Delta\Psi$m cells, indicating that mitochondrial activity promotes cellular fitness (Figure 3E). To further test the connection between r$\Delta\Psi$m and cellular fitness and growth rate, we measured Jurkat cell $\Delta\Psi$m and protein synthesis rates with MitoTracker red and O-propargyl-puromycin (OPP), respectively. The r$\Delta\Psi$m explained up to 75% of the variability in protein synthesis rates (Figure 3F, R^2 = 0.74), suggesting that mitochondrial activity is a key factor behind variability in cellular growth rate. Altogether, these experiments indicate that intermediate-sized cells within a population have the best cellular fitness, which is supported by optimal mitochondrial activity and minimal mitochondrial variability.

Cells Aim to Maintain Their Optimal Cell Size

We next analyzed whether cells separated by size return to their original size distribution, as this would be indicative of an optimal cell size (Figure 4A). Jurkat cells were elutriated into six fractions, and the mean sizes and size distributions of these subpopulations were measured at 0 hr and 100 hr. After 100 hr in culture, the median cell sizes of the subpopulations were indistinguishable from the median size of the original population (Figure 4B). As the resumption of normal cell size took a significant amount of time, the data suggest that potential cell size checkpoint(s) is/are not able to immediately reset cell size back to the optimal size. The narrower size distributions obtained by elutriation also broadened to resemble the original size distribution (Figure 4C). This widening of the size distribution also took place in the intermediate-sized subpopulation. While this might suggest that some cells are moving away from the optimal cell size, this widening of the size distribution mostly reflects the re-establishment of the normal cell-cycle distribution. This is because the selection of intermediate-sized cells by elutriation results in a cell population that is not only average in cell size but also highly enriched in cells in the middle of the cell cycle. Altogether, these data suggest that cells aim to maintain an optimal cell size at the population level. However, cells also maintain a size variation around the optimal cell size, as it is necessary to allow progression through the cell cycle. Similar results were observed in Kc167 cells (Figures S4D and S4E).

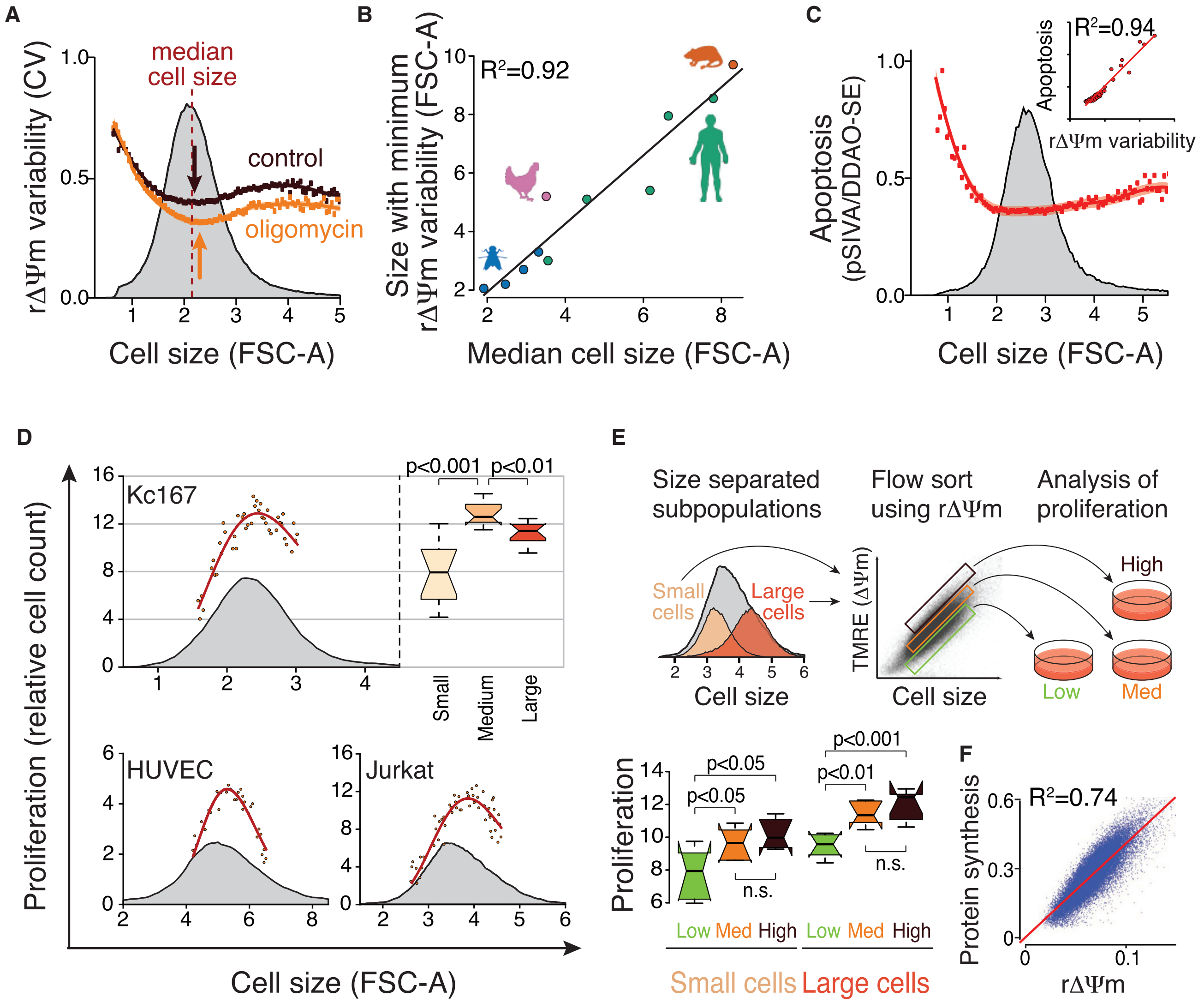

Figure 3. Cell Size Scaling of Mitochondrial Functions Establishes the Optimal Cell Size with Maximal Cellular Fitness

(A) Minimum of ΔΨm variability associates with median cell size. Same data as in Figure 1B were analyzed for cell-to-cell variability in ΔΨm. CV, coefficient of variability.

(B) Correlation between cell size with minimum ΔΨm variability and median cell size in cells of human (green), rat (orange), chicken (pink), and *Drosophila* (blue).

(C) Levels of apoptotic cells as a function of cell size as analyzed by an annexin-based biosensor (pSIVA) and normalized for cell size using cellular protein content using amino-reactive DDAO-SE dye (Miettinen and Bjorklund, 2015). Inset: Pearson correlation of apoptosis with ΔΨm variability in each size of cell. $n > 2.5 \times 10^5$ cells.

(D) Cell size scaling of overall cellular fitness, as measured by proliferative capacity. Kc167 cells, HUVECs, and Jurkat cells were separated to size-based subpopulations and equal numbers of cells were cultured for 72 hr (Kc167 and HUVECs) or 100 hr (Jurkat) before the final cell numbers were counted. Top right displays statistical analysis (ANOVA with Tukey's post hoc test) of Kc167 data (n = 12).

(E) Effect of rΔΨm on cell proliferation. Jurkat cells were first separated by size and then by flow sorting to populations with different rΔΨm. Cells were cultured for 100 hr (n = 6) and analyzed as in (D).

(F) Correlation between ΔΨm (MitoTracker red) and protein synthesis rate (OPP incorporation) in individual Jurkat cells ($n = 3.1 \times 10^4$ cells). Both fluorescence signals were normalized to cell size (FSC-A).

See also Figure S4.

Insulin Signaling Controls the Optimal Cell Size, but Not the Allometry, in Mitochondrial Functionality

Growth factor signaling is a key determinant in controlling average cell size at the population level in multicellular organisms, the insulin/mTOR (mammalian target of rapamycin) pathway being particularly important for cell size (Lloyd, 2013). To analyze how insulin signaling contributes to the optimal cell size and mitochondrial functional scaling, we treated Kc167 cells with insulin and rapamycin. While rapamycin decreased and insulin increased the cell size, scaling of mitochondrial functionality followed the new cell size distribution. The scaling remained nearly identical, as quantified by analyzing the correlations

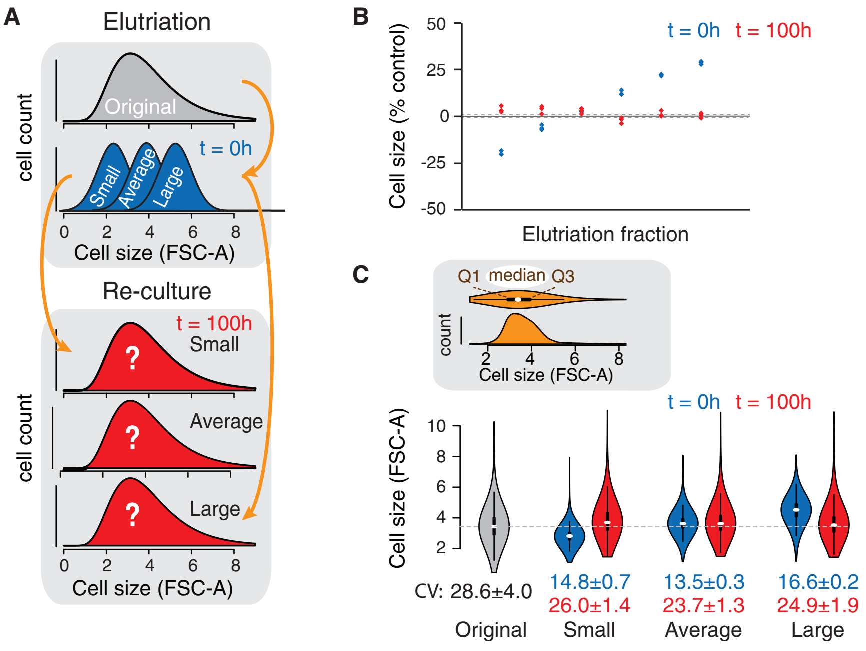

Figure 4. Cells Aim to Maintain Optimal Cell Size

(A) Schematic of the experimental setup. Cells are separated into size-based subpopulations by elutriation and returned to culture. After 100 hr, mean cell size and size distribution are measured using flow cytometry to see whether the cell populations maintain their original median size and discover the resulting cell size distribution.

(B) Mean cell size in each Jurkat cell subpopulation immediately after elutriation (0 hr, blue) and after 100 hr in culture (red). The data shown represent percent change versus unsorted original population. The size changes after 100 hr are statistically nonsignificant (two-tailed t test) (n = 3 in each subpopulation).

(C) Size distributions of the original Jurkat cell population as well as smallest, average-sized, and largest subpopulations after 0 hr (blue) and 100 hr (red). The cell size distributions are illustrated with violin plots, and the inset above displays a comparison between a typical density plot and a violin plot representation of the data. White oval represents the median size and the thicker black line shows the first and third quartile (Q1 and Q3, where 25% and 75% of the data are below these points, respectively). CV is shown below the size distribution plots. Data shown represent mean ± SD (n = 3) from 1–5 × 10^4 cells per sample. The gray dotted line indicates the median cell size in the original population.

See also Figure S4.

between cell size normalized rΔΨm medians and variabilities (R^2 = 0.94 and 0.96, respectively, Figure 5A). Similar results were seen in Jurkat cells (Figure S5). Thus, insulin/mTOR signaling controls the average cell size but does not affect the scaling of mitochondrial functionality or the association of minimum rΔΨm variability with median cell size. Insulin signaling, and possibly growth factors in general, therefore controls the optimal cell size, but not necessarily its establishment and maintenance through mitochondria. It is possible that, as mTOR promotes mitochondrial biogenesis, mTOR activity has a much larger impact on the mitochondrial content than function.

Mitochondrial Fusion and Fission Control the Cell Size Scaling of Mitochondrial Functions

We next wanted to identify which intrinsic processes are required for the observed allometric scaling of mitochondrial functionality. Changes in mitochondrial fusion and fission are tightly linked to ΔΨm (Chen et al., 2005; Legros et al., 2002) as well as mitochondrial homogeneity (Chan and Marshall, 2012). Furthermore, mitochondrial dynamics are predicted to affect cellular functions in a nonlinear manner (Hoitzing et al., 2015). We therefore asked whether mitochondrial fusion and fission affect the cell size scaling of mitochondrial functionality. Genetic inhibition of the mitochondrial dynamics by RNAi of the fission-regulating guanosine triphosphatase *Drp1* and the fusion-regulating mitofusins perturbed the cell size scaling of rΔΨm in Kc167 and Jurkat cells. Drp1 knockdown increased rΔΨm in larger cells, whereas mitofusin knockdown caused opposite effects (Figures 5B and 5C). As expected, a small-molecule inhibitor of mitochondrial division, Mdivi-1, induced effects similar to those of *Drp1* RNAi (Figure 5D). Inhibition of mitochondrial division also increased cell size. Furthermore, inhibition of mitochondrial fusion and fission also shifted the cell size with minimal ΔΨm variability (Figure 5B, right). These data suggest that mitochondrial dynamics or normal mitochondrial morphology is required for the typical cell size scaling of mitochondrial functions and the maintenance of optimal cell size. To further validate this, we analyzed Kc167 cell size scaling of mitochondrial respiration in the presence of Mdivi-1. In contrast to the untreated control cells (Figure 1E), Mdivi-1 treatment resulted in increased respiration in the largest cells (Figures 5E and S5F), as suggested by the changes in rΔΨm.

The Mevalonate Pathway Is Required for Cell Size Scaling of Mitochondrial Functions

The mevalonate pathway is important for cellular and organelle homeostasis by providing plasma membrane (cholesterol) and electron transport chain components (ubiquinones/coenzyme Q) as well as isoprenyls, which are required for post-translational modifications of proteins (Figure 6A). We previously identified the mevalonate pathway as a cell size regulator (Miettinen and Bjorklund, 2015; Miettinen et al., 2014), and this pathway has been shown to affect mitochondria (Andreux et al., 2014; Liu et al., 2014). We therefore examined how the mevalonate pathway affects the cell size scaling of mitochondrial functionality. In Kc167 cells inhibition of the mevalonate pathway by pitavastatin resulted in increased rΔΨm in larger cells (Figure 6B), consistent with the statin-induced ΔΨm increase in other cell types (Andreux et al., 2014). These effects were rescued by mevalonate, the metabolite immediately downstream of the statin target HMG-CoA reductase (Figure 6B). Further downstream of the mevalonate pathway the statin-induced scaling effects were

CellPress

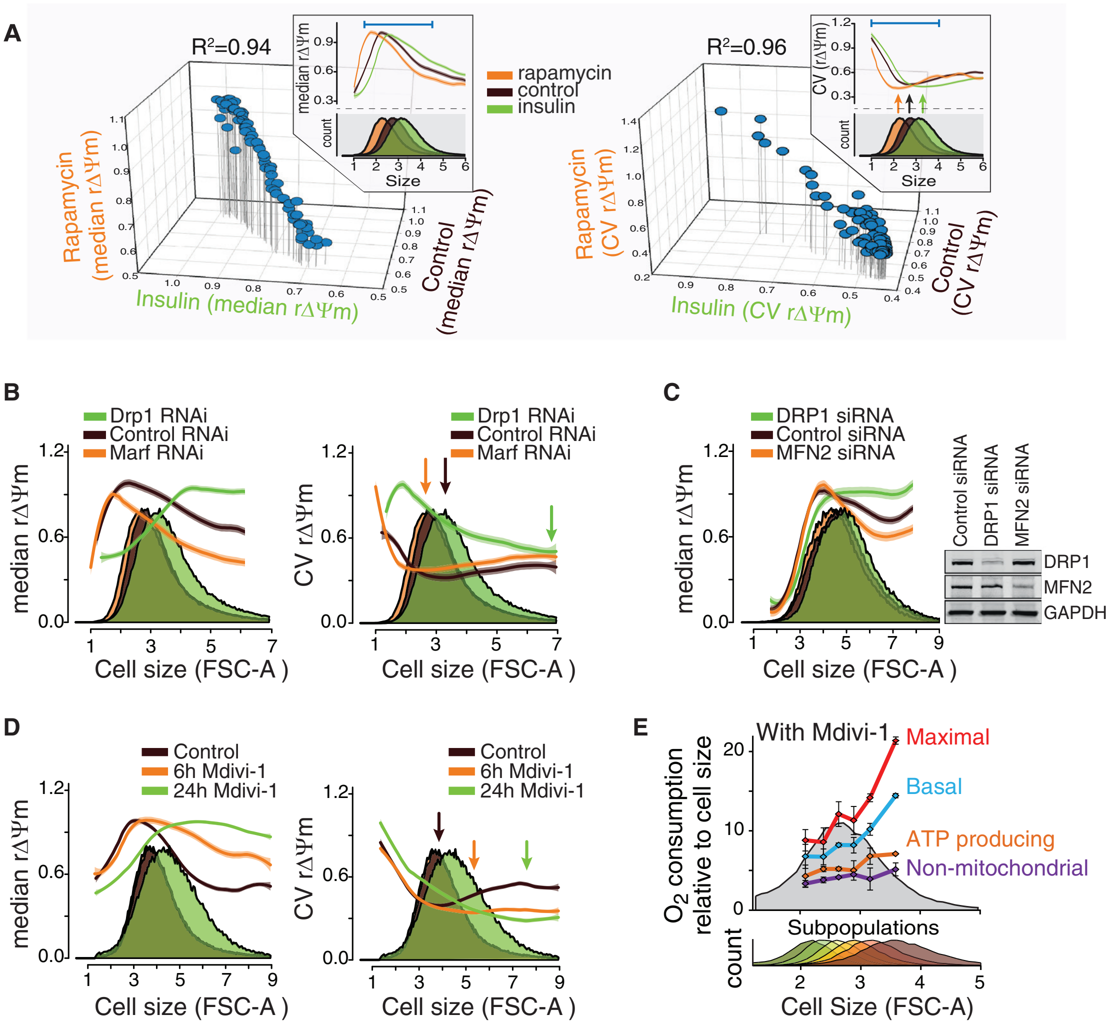

Figure 5. Mitochondrial Dynamics Regulate Cell Size Scaling of Mitochondrial Functions

(A) Insulin signaling controls optimal cell size without changing the scaling of rΔΨm. Kc167 cells were treated with insulin and rapamycin for 14 hr and rΔΨm was analyzed with JC-1 staining. The 3D plots display median (left) and variability (right) of rΔΨm for each cell size bin after normalizing for the overall cell size change. Insets display size distribution and scaling of rΔΨm and its variability. $n > 2 \times 10^5$ cells.

(B) Cell size scaling of rΔΨm (left) and its cell-to-cell variability (right) in Kc167 cells after 5 days of double-stranded RNA-mediated knockdown of Drp1 and Marf (mitofusin). Arrows indicate the cell size with the minimum variability. $n > 1.7 \times 10^5$ cells.

(C) Cell size scaling of rΔΨm in Jurkat cells after 65 hr of small interfering RNA-mediated knockdown of DRP1 and MNF2 (mitofusin 2). Inset displays western blot of the knockdown efficiency. $n > 1.2 \times 10^5$ cells.

(D) Kc167 cell rΔΨm (left) and variability (right) changes after 6 and 24 hr of treatment with the Drp1 inhibitor Mdivi-1. Arrows indicate the cell size with the minimum variability. $n > 2.5 \times 10^5$ cells.

(E) The changes in the rΔΨm caused by Mdivi-1 translate into increased oxygen consumption in the largest cells. Kc167 cells treated with Mdivi-1 were separated by centrifugal elutriation to size-based subpopulations (bottom panel) from which oxygen consumption parameters were analyzed. Oxygen consumption data represent mean ± SD (n = 3–4).

See also Figure S5.

rescued by farnesyl pyrophosphate and geranylgeranyl pyrophosphate (GGPP), isoprenyls required for protein prenylation (Figure 6B). Although ubiquinones are critical for mitochondrial electron transport chain function, the cell-permeable ubiquinone analog decylubiquinone did not rescue the statin phenotype (Figure 6B). Also, the statin-induced changes in rΔΨm cannot be due to cholesterol, as the Kc167 cells are incapable of de novo cholesterol synthesis. Consistently, inhibition of cholesterol synthesis downstream of farnesyl pyrophosphate did not change the cell size scaling of mitochondrial functionality (Figure S6A). Together, these results indicate that the mevalonate pathway and, more specifically, protein prenylation is required for the typical cell size scaling of rΔΨm observed in untreated cells.

We next investigated whether the mevalonate pathway could also affect the optimal cell size. Statin treatment dissociated the rΔΨm variability from the Kc167 median population cell size and this effect was rescued by GGPP supplementation (Figure 6C), suggesting that the mevalonate pathway is required for

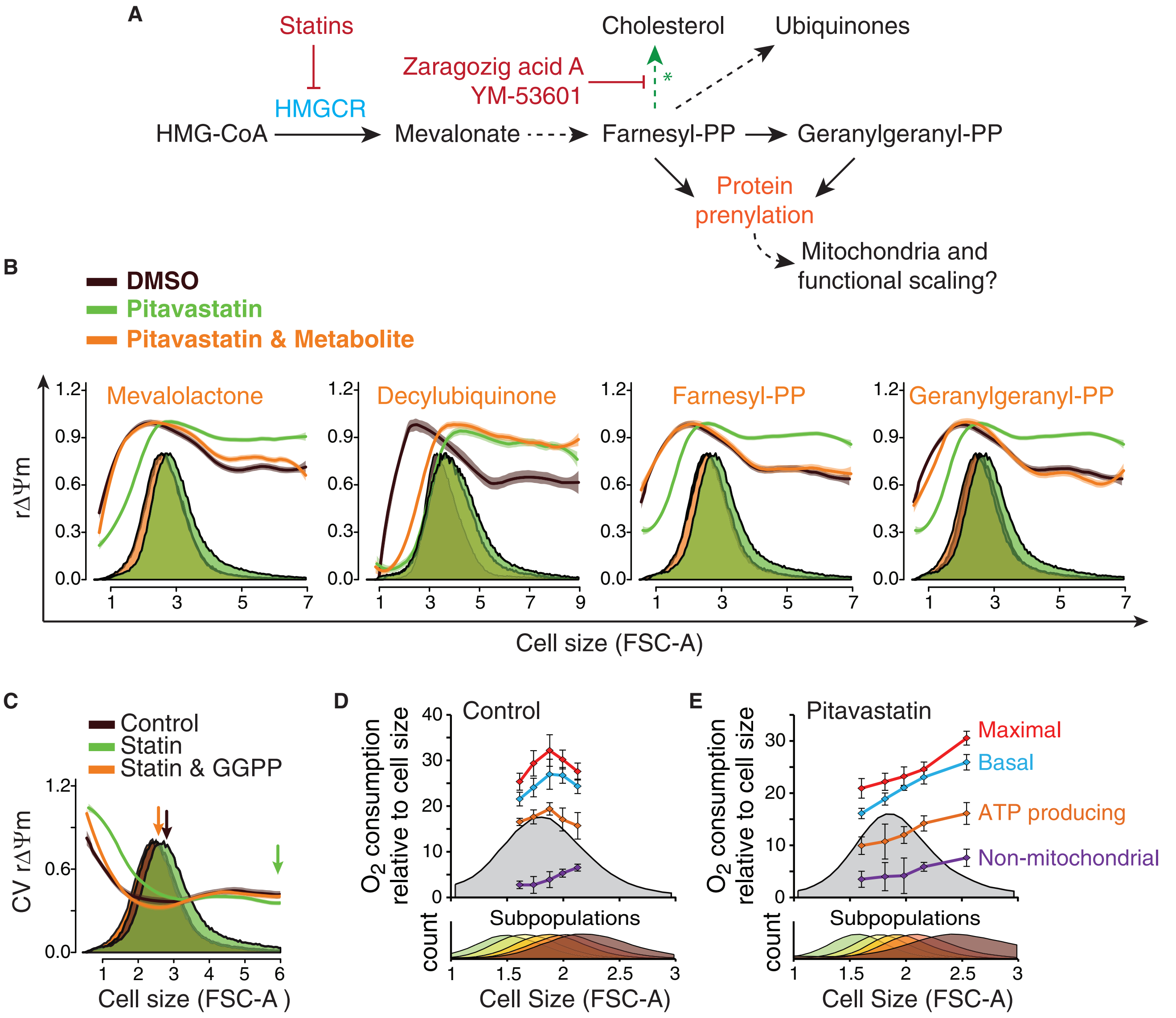

Figure 6. The Mevalonate Pathway-Mediated Protein Prenylation Is Required for Normal Cell Size Scaling of Mitochondrial Functions

(A) Schematic of the mevalonate pathway. Chemical inhibitors used are shown in red. *Note that *Drosophila* cells are incapable of de novo cholesterol synthesis.

(B) Mevalonate pathway regulates cell size scaling of rΔΨm through protein prenylation. Cell size scaling of rΔΨm in Kc167 cells treated with pitavastatin and indicated metabolites for 72 hr. $n > 2 \times 10^5$ cells.

(C) Cell size scaling of rΔΨm variability after 72 hr of treatment with statin. The statin effects were fully rescued by GGPP. Arrows indicate the cell size with the minimum variability. $n > 2 \times 10^5$ cells.

(D) Cell size scaling of respiration in control Kc167 cells. The experiment carried out as in Figure 1E. Data represent mean ± SD (n = 4).

(E) Same as (D), but cells were treated with statin for 72 hr before the oxygen consumption assay. Data represent mean ± SD (n = 2–4).

See also Figure S6.

maintaining the optimal cell size. We validated this by measuring the cell size-dependent oxygen consumption for statin-treated Kc167 cells. In contrast to the control cells (Figure 6D), where the relative oxygen consumption was reduced in larger cells (as also seen in Figure 1E), statin treatment caused oxygen consumption to increase even in the very largest cells (Figure 6E).

We also examined if the altered cell size scaling of rΔΨm could be due to inhibition of late autophagy and consequential mitochondrial accumulation (Miettinen and Bjorklund, 2015). Inhibitors of autophagy did not change the cell size scaling of rΔΨm (Figure S6B), and pitavastatin and Mdivi-1 treatments increased mitochondrial content proportionally to cell size (Figure S6C). Thus, altered autophagy and mitophagy cannot explain the statin-induced phenotype.

We next performed time-course analyses of Mdivi-1 and statin-induced changes in ΔΨm variability and population cell size to examine the relationship between these aspects in more detail. Mdivi-1 linearly increased cell size and changes in rΔΨm (Figure S6D). In contrast, rΔΨm, its variability, and cell size responded to pitavastatin after a 24-hr delay, further suggesting

that depletion of one or more prenylated proteins is necessary for these effects (Figure S6E). After this initial lag phase, both the cell size with minimum r$\Delta\Psi$m variability and the median population cell size slightly decreased, followed by a final increase. Overall, the median population cell size and r$\Delta\Psi$m changed with very similar time delays, indicating tight coupling of these processes. Finally, the statin-induced mitochondrial effects were not cell type or statin specific as they were also observed with another statin and in both Kc167 and Jurkat cells (Figure S6F). In conclusion, the mevalonate pathway is required for the cell size scaling of and the optimal cell size for mitochondrial functionality.

The Mevalonate Pathway Regulates Mitochondrial Size and Morphology

As the statin-induced mitochondrial functional scaling effects resembled those caused by Drp1 inhibition, we studied how the mevalonate pathway affects mitochondrial dynamics. We first isolated mitochondria from cells treated with statins and analyzed mitochondrial sizes using flow cytometry (Dabkowski et al., 2009; Song et al., 2015). Statin treatment of Jurkat and Kc167 cells increased the proportion of larger mitochondria, and this was prevented by addition of mevalonate or GGPP (Figures 7A and S7A). Microscopic observations validated that this analysis of isolated mitochondria could identify different-sized mitochondria, although the majority of them were round (Figure 7B). Thus, mitochondrial isolation allows size measurements but does not preserve mitochondrial morphology. We then examined the effects of mevalonate pathway inhibition on mitochondrial morphology using MitoTracker-red-stained and paraformaldehyde-fixed Jurkat and Kc167 cells. We found that the mevalonate pathway inhibition increases the number of enlarged tubular mitochondria (Figures 7C and S7B), consistent with observations in *Caenorhabditis elegans* (Liu et al., 2014). The morphology of statin-treated mitochondria was distinct from Mdivi-1-induced effects, and the statin effects were rescued by the addition of GGPP.

Mitochondrial morphology may be affected by fixation as well as mitochondrial dyes. To avoid such biases in our analysis, we further performed live cell experiments with Jurkat cells stained with MitoTracker green, which is structurally unrelated to MitoTracker red. MitoTracker-green-stained cells also displayed a more extensive tubular mitochondrial network in statin-treated cells than in control cells (Figures 7D and S7C). Quantitative analysis of mitochondrial volumes confirmed that statin-treated cells have significantly larger average mitochondrial size compared with controls ($p = 3.5 \times 10^{-6}$, two-tailed t test) (Figures 7E and S7D). This increase in mitochondrial size could, in theory, be due to inhibition of mitochondrial fragmentation. We therefore analyzed U2OS cells expressing mitochondria-targeted mCherry-GFP fusion protein (Allen et al., 2013) before and after disruption of the mitochondrial network by the mitochondrial uncoupler carbonyl cyanide 4-(trifluoromethoxy)phenylhydrazone (FCCP). U2OS cells display a well-connected mitochondrial network, which was efficiently fragmented by FCCP. This fragmentation could not be prevented, although it was potentially reduced in pitavastatin and Mdivi-1 treated cells (Figures 7F and 7G). Altogether, these analyses indicate that mitochondrial size and morphology is affected by the mevalonate pathway.

DISCUSSION

In contrast to the isometric cell size scaling of mitochondrial content, we report a nonproportional scaling of mitochondrial functions with cell size. Our results suggest that the physiological consequence of this nonlinear mitochondrial functional scaling is the establishment of an optimal cell size, as also supported by the known decline of growth rate in the largest cells (Maranon, 2015; Son et al., 2012; Tzur et al., 2009). These findings extend the allometric scaling laws on metabolism to the cellular level, as previously predicted (Kozlowski et al., 2003), and suggest that the allometric laws apply to the normal growth regime within a cell cycle. Nearly 20 years ago West, Brown, and Enquist provided a general model to explain allometric laws through transport of materials and energy through space-filling fractal networks of branching tubes (West et al., 1997). Only recently, it was shown that mitochondria may promote intracellular resource distribution by providing a conductive pathway for energy supply (Glancy et al., 2015). We now observe that the cellular allometry of mitochondrial metabolism is controlled by mitochondrial fusion and fission. It is thus possible that mitochondrial dynamics could influence cellular resource distribution by controlling the formation of a highly connected fractal-like mitochondrial network. This implies that a highly connected mitochondrial network could promote growth, improve fitness, and enable larger cell size by counteracting the biophysical limitations in resource distribution.

We also find that the mevalonate pathway affects mitochondrial dynamics and scaling of mitochondrial functionality. The mevalonate pathway, which produces various plasma membrane components, including cholesterol, could potentially act as a reporter for cell surface-to-volume ratio and relay this information to mitochondria via protein prenylation to maintain optimal cell size. It was recently shown that the levels of many proteins and metabolites in the mevalonate pathway are reduced in mitofusin knockout cells (Mourier et al., 2015), suggesting that mitochondrial dynamics can influence mevalonate pathway activity. This, together with our findings that the mevalonate pathway regulates cell size (Miettinen and Bjorklund, 2015) and mitochondrial dynamics, suggests that the mevalonate pathway and mitochondrial dynamics may constitute a growth and cell size regulatory network.

Finally, conditions such as cardiac hypertrophy and cellular aging are characterized by abnormal increases in cell size and reduced mitochondrial functionality. The potential implication of our current and previous (Miettinen and Bjorklund, 2015; Miettinen et al., 2014) observations is that changes in cell size may affect mitochondrial functionality. Thus, cell size may affect mitochondrial functions in various physiological and pathological settings (Lloyd, 2013; Nunnari and Suomalainen, 2012), making cell size-induced metabolic changes directly relevant for development and disease.

EXPERIMENTAL PROCEDURES

Full details are provided in Supplemental Experimental Procedures.

Cell Culture and Chemical Treatments

Kc167 cells were cultured in Schneider's *Drosophila* medium supplemented with 10% fetal bovine serum (FBS), penicillin, and streptomycin. Jurkat cells were cultured in high-glucose RPMI supplemented with 10% FBS, penicillin,

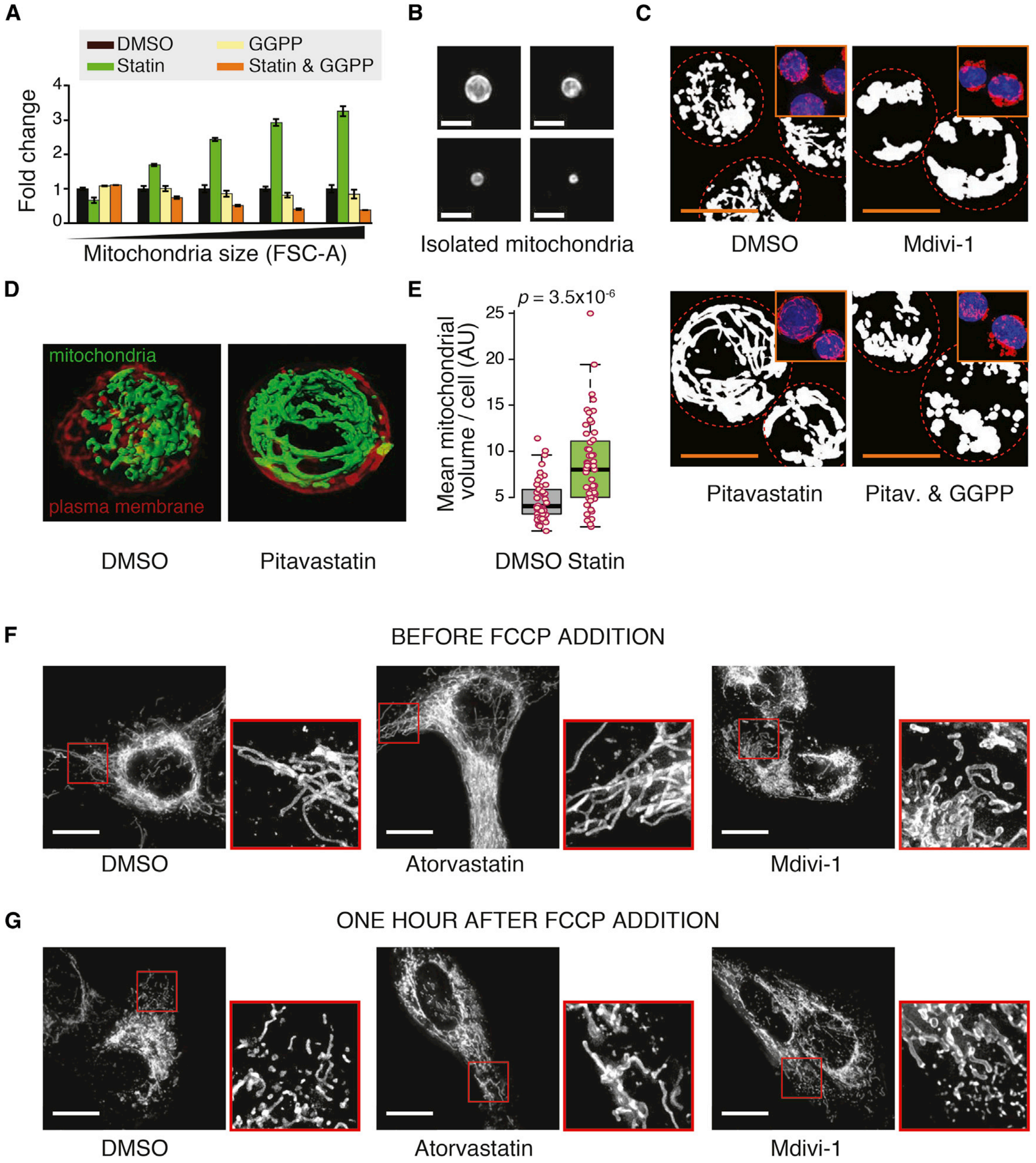

Figure 7. The Mevalonate Pathway Regulates Mitochondrial Volume and Morphology

(A) Statins increase the size of isolated mitochondria. Flow cytometry-based size analysis of isolated mitochondria from Jurkat cells. Cells were treated with indicated chemicals for 72 hr. Data represent mean ± SD (n = 3).

(B) Representative maximum-intensity projections of MitoTracker-green-stained, isolated mitochondria from Jurkat cells. Scale bars, 2.5 μm.

(C) Representative binary images of Jurkat cell mitochondria. Insets show maximum-intensity projections of fixed Jurkat cells stained with DAPI (nucleus, blue) and MitoTracker red (mitochondria, red) after treatment with indicated chemicals. Statin treatment was for 72 hr and Mdivi-1 treatment for 12 hr. Scale bars, 10 μm.

(D) Representative 3D projections of live Jurkat cell mitochondria after 72 hr of treatment with statin. Cells were stained with MitoTracker green and CellMask Deep Red plasma membrane stain.

(E) Statins increase mitochondrial volume in live cells. Quantification of average mitochondrial volume within each Jurkat cell from samples shown in (D) (n = 48 [control] and 51 [pitavastatin], p value is from a t test).

(F) Representative maximum-intensity projections, and their zoom-ins, of U2OS cells expressing mitochondria-targeted mCherry-GFP fusion after 72 hr of statin treatment or 24 hr of Mdivi-1 treatment. Scale bars, 10 μm.

(G) Same as (F), except that cells were treated with 2 μM FCCP for 1 hr to induce mitochondrial fragmentation. Scale bars, 10 μm.

See also Figure S7.

streptomycin, and L-glutamine. In addition, the following cell lines were used in some experiments: yeast BY4741 cells, *Drosophila* cells, Clone 8 and S2R+ cells, chicken DT40 cells, and human NB4 and U2OS stably expressing mCherry-GFP-FIS1 (amino acids 101–152) cells. Primary HUVECs and rat hepatocytes were from Life Technologies. All cell lines have been tested negative for mycoplasma. All chemicals were obtained from Sigma-Aldrich, unless otherwise stated. Concentrations used were 1 μM CCCP, 10 μM oligomycin, 50 μM Mdivi-1, 5 μM pitavastatin, 5 μM atorvastatin, 40 μM rosuvastatin, 20 μM decylubiquinone, 20 μM farnesyl pyrophosphate, 20 μM GGPP, and 0.2 mM mevalolactone. Note that the used statin concentrations exhibit only a modest toxicity in the cell lines used (Miettinen and Bjorklund, 2015), that cell toxicity does not explain the altered cell size scaling of rΔΨm (data not shown), and that the statin-induced rΔΨm effects were seen in *Drosophila* cells, which are incapable of de novo cholesterol synthesis.

Fluorescence Probes

A common oversight in cell biology is that fluorescent markers are not normalized to cell size. We used JC-1 dye (Smiley et al., 1991) to measure ΔΨm (unless otherwise indicated), as the ratiometric nature of this probe inherently normalizes the data with mitochondrial content, but also to cell size due to isometric scaling of mitochondrial content (see Figures 1B and S2G). Thus JC-1 dye reports the relative (cell size normalized) ΔΨm (rΔΨm). In a typical experiment, JC-1 staining time was 45 min with 2 μM dye concentration. Stainings were performed at the normal cell-culture temperature for each cell line (23.5°C for *Drosophila* cells, 37°C for mammalian cells). All fluorescence intensities, cell counts, and sizes were measured using an Accuri C6 flow cytometer (Becton Dickinson).

Computational Cell Size Relation Analysis of ΔΨm

To analyze the fluorescence signals, and thus cell behavior, as a function of cell size, we devised a new flow cytometer-based single-cell approach dubbed computational size relation analysis (CoSRA). Single-cell data falling into a viable cell gate, as estimated using plasma membrane integrity based on propidium iodide exclusion was used to define the viable cell population (Figure S2B). Data were exported from the flow cytometer and analyzed in R environment (R version 3.1.0). FL2-A/FL1-A ratio representing the JC-1 aggregate/monomer ratio was calculated for each cell and the cells were then computationally fractionated into size-based subpopulations (these bins are typically 5×10^4 FSC-A units, which corresponds to approximately 100 nm in diameter based on calibration data presented in Figures S1C and S1D). Median fluorescence intensities were calculated for each bin. When comparing the cell size scaling of rΔΨm after different cellular perturbations, the median rΔΨm values were normalized to the bin with the highest median rΔΨm. Oligomycin, CCCP, and valinomycin treatments were normalized to the maximum of the control sample. A local polynomial regression (loess) curve was fitted to the median values using the built-in R function *loess*. The 95% confidence intervals for the loess curve were estimated using the predict function. Finally, medians (dots), loess regression line (solid curve), and confidence intervals for the regression line (shaded area) were plotted as a function of cell size (FSC-A) in the same plot with a cell size distribution histogram. The global minima of the variability were identified from the loess regression fit and indicated with vertical arrows. To increase figure clarity, we have removed individual data points (median or coefficient of variability [CV] of rΔΨm in each bin) from most figures. The resulting regression curve displays the typical trajectory of the measured parameter as a function of cell size. Note that the CoSRA approach is not limited to analyzing the cell size scaling of rΔΨm, as any cellular parameter for which a suitable fluorescence-based reporter is available can be used (see Figure S1 for examples).

Analyzing the Contribution of Cell Size and Cell Cycle to the rΔΨm

To analyze the cell size dependency of ΔΨm, we applied mutual information analysis from Shannon's information theory. Shannon's information theory provides a general model by which to measure dependencies between variables, even if there is no linear dependence between these variables (Steuer et al., 2002). In information theory, mutual information is the average amount of information about U from observing the value of V. In particular, conditional mutual information expressed as I(U; V|W) provides an estimate of the quantity of information shared between U and V when W is known (fixed). We can thus estimate the association between rΔΨm and cell size (S), when the cell-cycle (CC) status of the cells is known, i.e., I(rΔΨm; S|CC). If cell size S carries information regarding rΔΨm, which is not already contained in CC information, the conditional mutual information yields a value >0. Zero value means lack of additional information between the variables rΔΨm and S, given that CC is known. Mutual information can thus have values of zero to infinity (0 … ∞); however, this value directly has no obvious interpretation, but by comparing I(rΔΨm; S|CC) and I(rΔΨm; CC|S), the relative information content carried by size and cell cycle can be analyzed.

Size Separation by Centrifugal Elutriation

To examine size-dependent changes using population-based assays, we separated cells into size-based subpopulations using centrifugal elutriation. Approximately 3×10^8 cells were resuspended in 3 mL of PBS with 1% FBS. Cells were loaded into a counterflow centrifugal elutriator (Beckman JE-5.0/JE), equipped with a standard elutriation chamber and a peristaltic pump (see Supplemental Experimental Procedures for details). Collected cells were resuspended in culture medium, and cell size and counts were analyzed using flow cytometry. Mitochondrial mass and membrane potential from elutriated fractions were measured using MitoTracker green FM and MitoTracker red CMXRos, respectively. Flow cytometry-measured MitoTracker intensities were normalized to mean FSC-A values.

Oxygen Consumption Measurements

Oxygen consumption was measured using an XF24 Extracellular Flux Analyzer (Seahorse Bioscience). The assay plates were coated with poly-L-lysine, and 80,000 Kc167 cells were plated on each well 2 hr before the analysis. The analysis was carried out in Schneider's cell-culture medium with 10% FBS at room temperature. Oxygen consumption was measured every 6 min and the following injections were performed after every four measurements: (1) 1 μM oligomycin, (2) 1 μM FCCP, and (3) 2 μM rotenone and 2 μM antimycin A (see Supplemental Experimental Procedures for details). For each replicate the four oxygen consumption measurements between each injection were averaged, after which the respiratory parameters were calculated. All the results were normalized to the subpopulation cell size (FSC-A). The analysis was also carried out after treating cells with the mitochondrial division inhibitor Mdivi-1 (50 μM) for 24 hr or pitavastatin (5 μM) for 72 hr. Mdivi-1 and pitavastatin were present for the whole duration of the oxygen consumption assay.

Proliferation Measurements

For proliferation-based fitness measurements, Kc167 cells, Jurkat cells, and HUVECs were first separated into size-based subpopulations using centrifugal elutriation. The average cell size in each subpopulation was measured using flow cytometry, and an equal cell count of each subpopulation was taken for further culture in normal conditions. The final data are presented as relative cell counts after indicated culture time (y axis) as a function of the initial subpopulation cell size (x axis). The total cell population histogram is also shown for reference. For Kc167 cells the differences in proliferative capacity of different-sized cells were highlighted by presenting the data as a box plot.

The effect of mitochondrial membrane potential on cell proliferation was analyzed in Jurkat cells by first using centrifugal elutriation to separate two different-sized subpopulations. These cells were then stained with TMRE and cells with high, medium, and low rΔΨm (thus taking cell size into account in assessing the ΔΨm, see schematic in Figure 2E) were separated using flow sorting (Influx Cell Sorter, Becton Dickinson), and cell sizes were reanalyzed in the separated populations to validate that rΔΨm-based separation did not change the average cell size. Equal cell counts of these rΔΨm-separated cells were then cultured for 100 hr in normal growth conditions, after which cells were recounted.

When analyzing cell proliferation using the CoSRA method (see above), cells were first stained using DDAO-SE dye and the baseline staining levels were measured using flow cytometry. The cells were then cultured normally for 48 hr after which cells were reanalyzed for DDAO-SE stain dilution (see Figure S5A).

Protein Synthesis Measurements

Jurkat cells were stained with Click-iT Plus OPP Alexa Fluor 488 Protein Synthesis Assay Kit (LifeTechnologies) and MitoTracker red CMXRos. OPP and

MitoTracker red were added to 20 μM and 200 nM final concentration, respectively, and incubated for 30 min. Cells were fixed with 3.7% formaldehyde and processed as instructed in the OPP assay kit. Cells were analyzed using flow cytometry.

Microscopy

For examination of mitochondrial morphology, Kc167 and Jurkat cells were treated with indicated chemicals and moved to coverslips coated with 0.1% poly-L-lysine. The cells were stained with MitoTracker red CMXRos or MitoTracker green with CellMask Deep Red. U2OS cells expressing mCherry-GFP were imaged in the absence of any stains. For live cell imaging, cells were incubated at 37°C and 5% CO_2 in complete culture medium during the experiment. Cells were imaged with a DeltaVision wide-field deconvolution microscope using standard filters (DAPI, fluorescein isothiocyanate, tetramethylrhodamine isothiocyanate, Cy5) and 100× objective. For details of image processing, see Supplemental Information.

Statistical Analyses

Pearson correlations (R^2) were calculated using the square of the *cor* function in R. When comparing the cell size scaling of r$\Delta\Psi$m after modulations to the insulin signaling pathway, the correlations between the median r$\Delta\Psi$m values in each cell size bin were calculated after normalizing for the cell size change caused by insulin or rapamycin. Statistical significances were calculated using one-way ANOVA and Tukey's post hoc test or t test as indicated. Conditional mutual information was calculated using the *condinformation* function in the infotheo package in R. The statistical significance of the mutual information values was assessed from replicate samples using a two-tailed t test.

SUPPLEMENTAL INFORMATION

Supplemental Information includes Supplemental Experimental Procedures and seven figures and can be found with this article online at http://dx.doi.org/10.1016/j.devcel.2016.09.004.

AUTHOR CONTRIBUTIONS

T.P.M. and M.B. performed the experiments, analyzed the data, and wrote the paper. M.B. conceived the study.

ACKNOWLEDGMENTS

We thank A. Lloyd, J. Bähler, and S. Manalis for comments, A. Irazoki and A. McLeod for technical assistance, T. Ly for advice on elutriation, M. Posch for assistance with imaging, R. Clarke for cell sorting, and L. Albergante and P. Miettinen for advice on information theory. This study was funded by the Scottish Universities Life Sciences Alliance (SULSA) and the Wellcome Trust Career Development Fellowship (grant 089999/Z/09/Z) to M.B. The use of microscopy and flow sorting was supported by the Wellcome Trust Strategic Award grant 097945/Z/11/Z.

Received: February 15, 2016
Revised: June 27, 2016
Accepted: September 2, 2016
Published: October 6, 2016

REFERENCES

Allen, G.F., Toth, R., James, J., and Ganley, I.G. (2013). Loss of iron triggers PINK1/Parkin-independent mitophagy. EMBO Rep. *14*, 1127–1135.

Andreux, P.A., Mouchiroud, L., Wang, X., Jovaisaite, V., Mottis, A., Bichet, S., Moullan, N., Houtkooper, R.H., and Auwerx, J. (2014). A method to identify and validate mitochondrial modulators using mammalian cells and the worm *C. elegans*. Sci. Rep. *4*, 5285.

Chan, Y.H., and Marshall, W.F. (2012). How cells know the size of their organelles. Science *337*, 1186–1189.

Chen, H., Chomyn, A., and Chan, D.C. (2005). Disruption of fusion results in mitochondrial heterogeneity and dysfunction. J. Biol. Chem. *280*, 26185–26192.

Dabkowski, E.R., Williamson, C.L., Bukowski, V.C., Chapman, R.S., Leonard, S.S., Peer, C.J., Callery, P.S., and Hollander, J.M. (2009). Diabetic cardiomyopathy-associated dysfunction in spatially distinct mitochondrial subpopulations. Am. J. Physiol. Heart Circ. Physiol. *296*, H359–H369.

Gillooly, J.F., Brown, J.H., West, G.B., Savage, V.M., and Charnov, E.L. (2001). Effects of size and temperature on metabolic rate. Science *293*, 2248–2251.

Ginzberg, M.B., Kafri, R., and Kirschner, M. (2015). Cell biology. On being the right (cell) size. Science *348*, 1245075.

Glancy, B., Hartnell, L.M., Malide, D., Yu, Z.X., Combs, C.A., Connelly, P.S., Subramaniam, S., and Balaban, R.S. (2015). Mitochondrial reticulum for cellular energy distribution in muscle. Nature *523*, 617–620.

Glazier, D.S. (2005). Beyond the '3/4-power law': variation in the intra- and interspecific scaling of metabolic rate in animals. Biol. Rev. Camb. Philos. Soc. *80*, 611–662.

Hoitzing, H., Johnston, I.G., and Jones, N.S. (2015). What is the function of mitochondrial networks? A theoretical assessment of hypotheses and proposal for future research. Bioessays *37*, 687–700.

Kitami, T., Logan, D.J., Negri, J., Hasaka, T., Tolliday, N.J., Carpenter, A.E., Spiegelman, B.M., and Mootha, V.K. (2012). A chemical screen probing the relationship between mitochondrial content and cell size. PLoS One *7*, e33755.

Kleiber, M. (1932). Body size and metabolism. Hilgardia *6*, 315–353.

Kozlowski, J., Konarzewski, M., and Gawelczyk, A.T. (2003). Cell size as a link between noncoding DNA and metabolic rate scaling. Proc. Natl. Acad. Sci. USA *100*, 14080–14085.

Legros, F., Lombes, A., Frachon, P., and Rojo, M. (2002). Mitochondrial fusion in human cells is efficient, requires the inner membrane potential, and is mediated by mitofusins. Mol. Biol. Cell *13*, 4343–4354.

Liu, Y., Samuel, B.S., Breen, P.C., and Ruvkun, G. (2014). Caenorhabditis elegans pathways that surveil and defend mitochondria. Nature *508*, 406–410.

Lloyd, A.C. (2013). The regulation of cell size. Cell *154*, 1194–1205.

Ly, T., Ahmad, Y., Shlien, A., Soroka, D., Mills, A., Emanuele, M.J., Stratton, M.R., and Lamond, A.I. (2014). A proteomic chronology of gene expression through the cell cycle in human myeloid leukemia cells. Elife *3*, e01630.

Maranon, E. (2015). Cell size as a key determinant of phytoplankton metabolism and community structure. Annu. Rev. Mar. Sci. *7*, 241–264.

Miettinen, T.P., and Bjorklund, M. (2015). Mevalonate pathway regulates cell size homeostasis and proteostasis through autophagy. Cell Rep. *13*, 2610–2620.

Miettinen, T.P., Pessa, H.K., Caldez, M.J., Fuhrer, T., Diril, M.K., Sauer, U., Kaldis, P., and Bjorklund, M. (2014). Identification of transcriptional and metabolic programs related to mammalian cell size. Curr. Biol. *24*, 598–608.

Mourier, A., Motori, E., Brandt, T., Lagouge, M., Atanassov, I., Galinier, A., Rappl, G., Brodesser, S., Hultenby, K., Dieterich, C., et al. (2015). Mitofusin 2 is required to maintain mitochondrial coenzyme Q levels. J. Cell Biol. *208*, 429–442.

Nunnari, J., and Suomalainen, A. (2012). Mitochondria: in sickness and in health. Cell *148*, 1145–1159.

Posakony, J.W., England, J.M., and Attardi, G. (1977). Mitochondrial growth and division during the cell cycle in HeLa cells. J. Cell Biol. *74*, 468–491.

Rafelski, S.M., Viana, M.P., Zhang, Y., Chan, Y.H., Thorn, K.S., Yam, P., Fung, J.C., Li, H., Costa Lda, F., and Marshall, W.F. (2012). Mitochondrial network size scaling in budding yeast. Science *338*, 822–824.

Reber, S., and Goehring, N.W. (2015). Intracellular scaling mechanisms. Cold Spring Harb. Perspect. Biol. *7*, http://dx.doi.org/10.1101/cshperspect.a019067.

Schmoller, K.M., and Skotheim, J.M. (2015). The biosynthetic basis of cell size control. Trends Cell Biol. *25*, 793–802.

Shannon, C.E. (1948). A mathematical theory of communication. Bell Syst. Tech. J. *27*, 379–423.

Smiley, S.T., Reers, M., Mottola-Hartshorn, C., Lin, M., Chen, A., Smith, T.W., Steele, G.D., Jr., and Chen, L.B. (1991). Intracellular heterogeneity in

mitochondrial membrane potentials revealed by a J-aggregate-forming lipophilic cation JC-1. Proc. Natl. Acad. Sci. USA *88*, 3671–3675.

Son, S., Tzur, A., Weng, Y., Jorgensen, P., Kim, J., Kirschner, M.W., and Manalis, S.R. (2012). Direct observation of mammalian cell growth and size regulation. Nat. Methods *9*, 910–912.

Song, M., Mihara, K., Chen, Y., Scorrano, L., and Dorn, G.W., 2nd (2015). Mitochondrial fission and fusion factors reciprocally orchestrate mitophagic culling in mouse hearts and cultured fibroblasts. Cell Metab. *21*, 273–285.

Steuer, R., Kurths, J., Daub, C.O., Weise, J., and Selbig, J. (2002). The mutual information: detecting and evaluating dependencies between variables. Bioinformatics *18* (*Suppl 2*), S231–S240.

Tzur, A., Kafri, R., LeBleu, V.S., Lahav, G., and Kirschner, M.W. (2009). Cell growth and size homeostasis in proliferating animal cells. Science *325*, 167–171.

West, G.B., Brown, J.H., and Enquist, B.J. (1997). A general model for the origin of allometric scaling laws in biology. Science *276*, 122–126.

Developmental Cell, Volume *39*

Supplemental Information

Cellular Allometry of Mitochondrial Functionality Establishes the Optimal Cell Size

Teemu P. Miettinen and Mikael Björklund

INVENTORY OF SUPPLEMENTARY MATERIALS:

Figure S1. Cellular organelle contents scales isometrically with cell size and flow cytometer FSC-A value provides accurate measurements of cell size, related to Figure 1.

Figure S2. Establishing computational size relation analysis (CoSRA) for r$\Delta\Psi$m in different cell types, related to Figure 1.

Figure S3. Cell size scaling of r$\Delta\Psi$m in different cell cycle phases, related to Figure 2.

Figure S4. Analysis of optimal cell size by dye dilution and elutriation, related to Figures 3 and 4.

Figure S5. Mitochondrial dynamics and not mTOR regulate cell size scaling of mitochondrial functions , related to Figure 5.

Figure S6. Cell size scaling of r$\Delta\Psi$m requires the mevalonate pathway and cannot be explained by increased mitophagy in larger cells, related to Figure 6.

Figure S7. Mitochondrial size and morphology homeostasis require the mevalonate pathway activity, related to Figure 7.

Supplemental Experimental Procedures

Supplemental References

SUPPLEMENTAL FIGURES

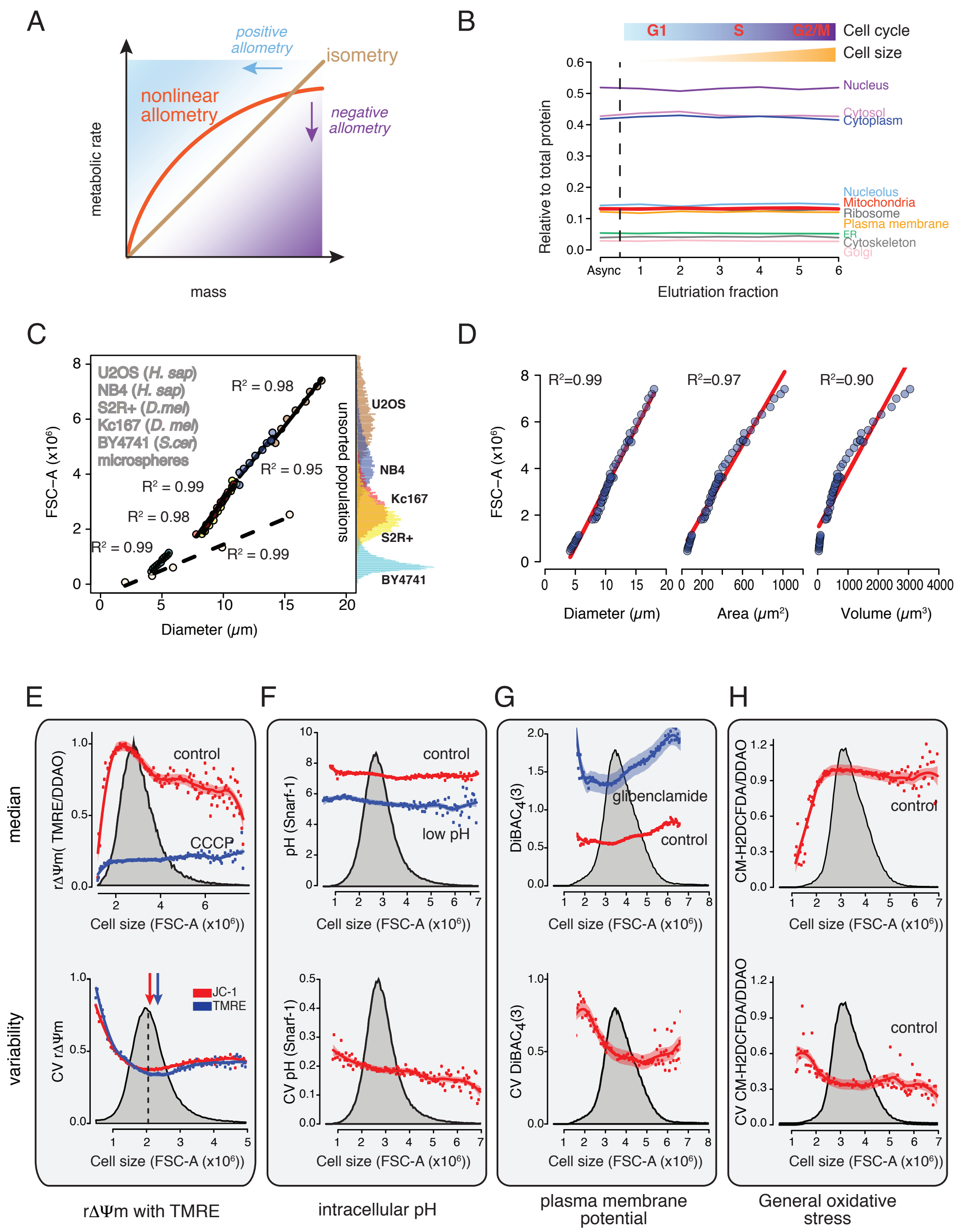

Figure S1. Cellular organelle contents scales isometrically with cell size and flow cytometer FSC-A value provides accurate measurements of cell size, related to Figure 1.

(A) Schematic presentation of different types of size scaling. Scaling where Y, for example metabolic rate, increases proportionally with mass (X) is isometric, whereas non-proportional scaling is allometric.

(B) Cell size scaling of relative proteome content associated with different subcellular compartments and organelles in NB4 leukemic cells. The data was normalised to the total protein content in the absence of absolute quantifications of protein levels. All organelle/protein groups examined display, on average, isometric cell size scaling during the normal cell cycle in an unperturbed cell population.

(C) Cultures of U2OS (human), NB4 (human), Kc167 (fruit fly), S2R+ (fruit fly) and BY4741 (yeast) cells were separated to different sized subpopulations using centrifugal elutriation. The mean size of each subpopulation was then assessed using electronic volume measurements (Casy TT) and flow cytometer measurements. Pearson correlations of the cell diameter, as measured using electronic volume measurements (x-axis), with FSC-A values (y-axis) are shown for each cell type assuming that cell size is spherical. In addition, data from polystyrene microbeads of known sizes are shown (light yellow markers and dashed line). Distributions of unsorted cell populations, as measured by flow cytometry, are shown on the right side of the plot.

(D) Correlations between FSC-A and electronic volume measurements based on cell diameter, area and volume from all cell measurements shown in (C). R^2 depicts Pearson correlation coefficient. Note, that although FSC-A is linearly best correlated with the cell diameter, absolute values of FSC-A may change over longer time periods due to instrument optical drift and thus cannot be directly used to obtain absolute cell sizes.

(E) Cell size scaling of r$\Delta\Psi$m as measured by TMRE and DDAO-SE staining. Kc167 cells were stained with DDAO-SE to label cellular protein content, after which $\Delta\Psi$m was measured using TMRE staining. TMRE was normalised to cellular protein content. Control and 1μM CCCP treated cells are shown. Bottom panel displays the cell size scaling of $\Delta\Psi$m cell-to-cell variability. Median cell size is indicated with a dotted black line, while arrows indicate the location of the minimum variability. Note that the ratiometric measurement of JC-1 normalises the $\Delta\Psi$m to mitochondrial mass, but as similar results are obtained without normalisation with TMRE. This suggests that the location of variability minimum depends on $\Delta\Psi$m, not mitochondrial content.

(F) Cell size scaling of intracellular pH was measured by staining Kc167 cells with SNARF-1 (top panel). Low pH control was made by incubating the cells in pH 5.5 buffer in the presence of valinomycin and nigericin for 10 min. pH was calibrated using the Intracellular pH Calibration Buffer Kit (Invitrogen). Note that SNARF-1 is a ratiometric dye and therefore further normalization to cell size is not required. Bottom panel displays the cell size scaling of intracellular pH cell-to-cell variability.

(G) Cell size scaling of plasma membrane potential ($\Delta\Psi p$) was measured by staining Jurkat cells with DiBAC$_4$(3) (top panel). DiBAC$_4$(3) entry into the cells is inhibited by high $\Delta\Psi p$. The negative control, 50μM glibenclamide, depolarizes plasma membrane and allows DiBAC$_4$(3) entry. The results were normalised to DDAO-SE staining. DiBAC$_4$(3) staining in Kc167 cells results in more linear cell size scaling of $\Delta\Psi p$, but glibenclamide was ineffective in depolarizing the Kc167 cell plasma membrane (data not shown). Bottom panel displays the cell size scaling of $\Delta\Psi p$ cell-to-cell variability.

(H) Cell size scaling of general oxidative stress was measured in Jurkat cells by CM-H2DCFDA staining which was normalized to total protein using content (DDAO-SE staining). Bottom panel displays the cell size scaling of oxidative stress cell-to-cell variability.

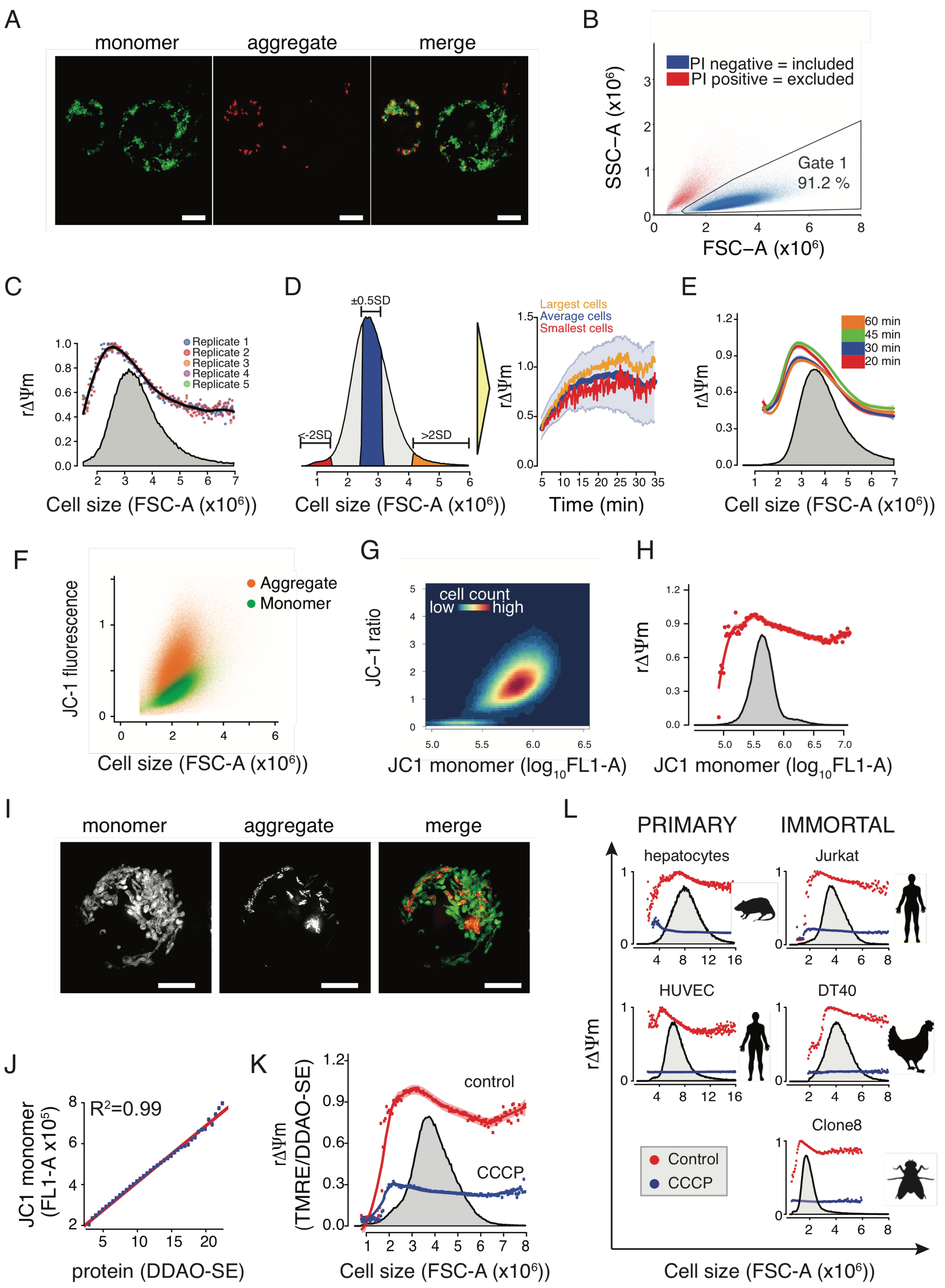

Figure S2. Establishing computational size relation analysis (CoSRA) for rΔΨm in different cell types, related to Figure 1.

(A) Maximum intensity projections of a small and a large Kc167 cell stained with JC-1 and imaged using OMX structural illumination microscopy. Monomer form (green) of the dye localizes to mitochondria and forms aggregates (red) in $\Delta\Psi m$ dependent manner. The ratio between the monomer and the aggregate in each cell reports the cell size/mitochondrial content normalized, relative $\Delta\Psi m$ ($r\Delta\Psi m$). Scale bar $5\mu m$.

(B) Scatter plot of Kc167 cells stained with propidium iodide (PI) as a marker for cell viability (membrane integrity). SSC-A is the flow cytometer side scatter value. The main population (Gate 1) with mainly PI negative cells is used for all analyses.

(C) Reproducibility of CoSRA based $r\Delta\Psi m$ analysis as measured by JC-1 staining in Kc167 cells. Data shown is from five biological replicates, each with >100 000 cells.

(D) Analysis of JC-1 staining kinetics in Kc167 cells of different sizes. The cells were stained with JC-1 and signal intensity was measured over a 30 min time period. Change in JC-1 aggregate/monomer ratio ($r\Delta\Psi m$) was then analyzed separately for small cells, average sized cells and large cells, as indicated on the left panel. The right panel displays the staining kinetics. Light grey shading (right panel) indicates one standard deviation of the mean JC-1 ratio for the average sized cells.

(E) Alternative analysis of JC-1 staining kinetics in Kc167 cells. Cell size scaling of $r\Delta\Psi m$ is shown after different JC-1 staining times (20-60 min). Note that while JC-1 dye does not reach equilibrium, this does not affect the observed cell size scaling of $r\Delta\Psi m$.

(F) Scatter plot of JC-1monomer and aggregate as a function of cell size in Kc167 cells. Note that the $\Delta\Psi m$ dependent aggregate form displays much more variability than the mitochondrial mass dependent monomer form.

(G) Scatter plot of JC-1 ratio ($r\Delta\Psi m$) as a function of JC-1 monomer (mitochondrial content) in Jurkat cells.

(H) CoSRA style analysis of Jurkat cell $r\Delta\Psi m$, where data is displayed as a function of mitochondrial content (JC-1 monomer) instead of cell size. Due to the high correlation of JC1 monomer with cell size (see panel J), the median behavior of $r\Delta\Psi m$ is similar to that seen when analyzing $r\Delta\Psi m$ as a function of cell size.

(I) Maximum intensity projection of a Jurkat cell stained with JC-1 and imaged using OMX structural illumination microscopy. Similar to Kc167 cells (see panel A), the monomer form (green) of the dye localizes to mitochondria and in the presence of $\Delta\Psi m$ forms aggregates (red). Scale bar $5\mu m$.

(J) CoSRA analysis based correlation between JC-1 monomer signal and DDAO-SE based cellular protein staining in Jurkat cells. The near perfect Pearson correlation coefficient (R^2) validates that mitochondrial content scales linearly with cellular protein content, as seen in Kc167 cells (Figure 1B, inset).

(K) Cell size scaling of rΔΨm in Jurkat cells. ΔΨm was measured using TMRE and the signal was normalized to DDAO-SE based cellular protein content staining to obtain rΔΨm values.

(L) Cell size scaling of rΔΨm in various cell lines and primary cells. All cells were stained with JC-1 for 45 min either in the presence or absence of CCCP, except the rat primary hepatocytes, where valinomycin was used instead of CCCP. Note that all cell types, despite being vastly different sized, display similar rΔΨm scaling, where rΔΨm is low in the very smallest cells, peaks in cells which are smaller than average population size and then steadily declines towards larger cell size.

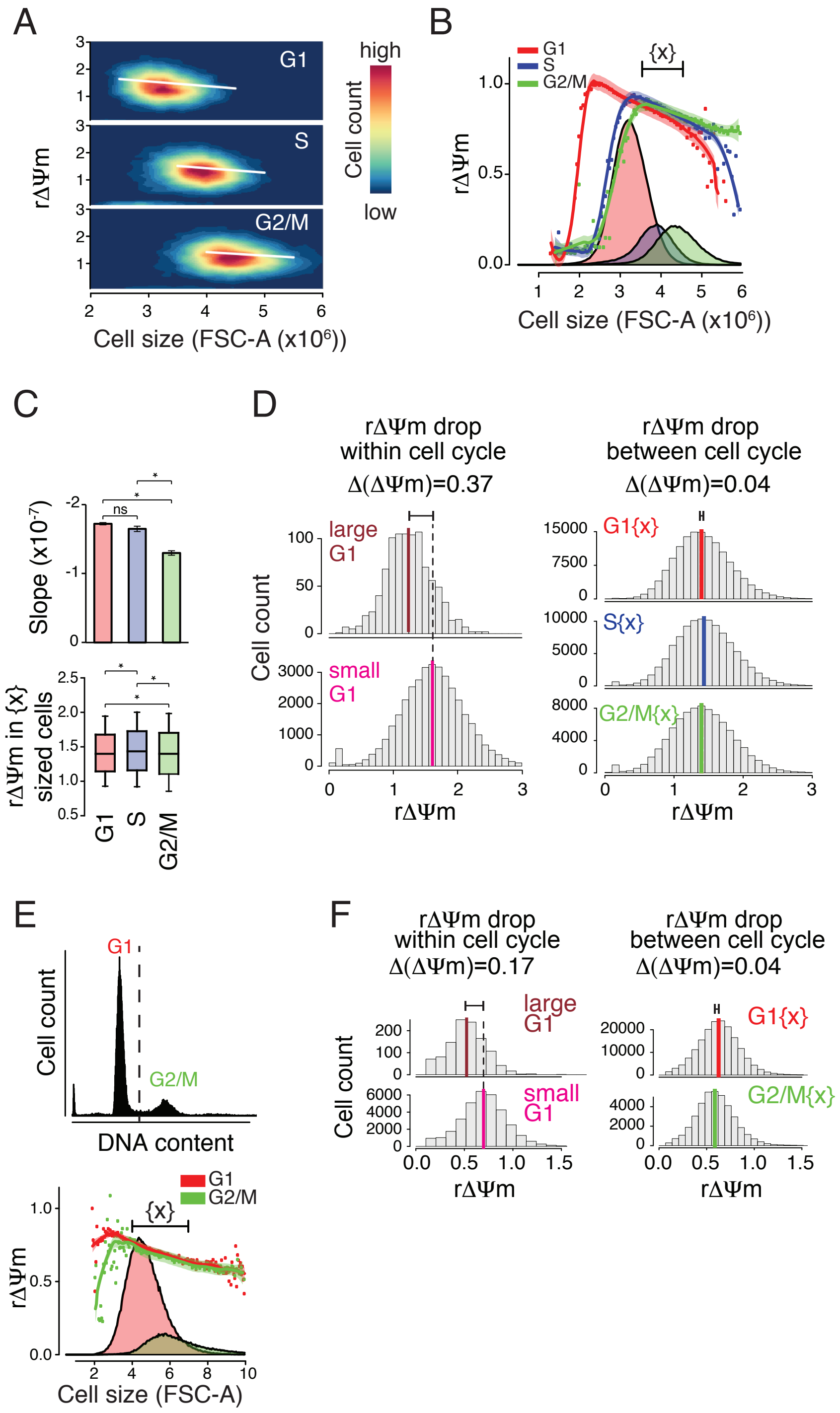

Figure S3. Cell size scaling of rΔΨm in different cell cycle phases, related to Figure 2.

(A) Jurkat cells were stained with JC-1 and Nuclear-ID Red DNA stain. G1, S and G2/M cells were separated based on DNA content and rΔΨm was plotted as a function of cell size. Linear regression curve (white line) was fitted excluding the smallest and largest cells (see experimental procedures for more details). Data is from 8.9×10^5 cells.

(B) CoSRA based analysis of rΔΨm as a function of cell size shown separately for each cell cycle phase. Cell size histograms of G1, S and G2/M cells are shown for reference. {x} displays the cell size area from which rΔΨm values were quantified. Same data as in (A).

(C) *Top,* Quantifications of the linear regression curve slopes in panel (A). Data is mean ± SEM. *Bottom,* quantifications of rΔΨm in cells of the same size, but different cell cycle phase (the cell size range {x} in panel (B)) ($n = 9x10^4$ cells). Note that while both quantifications provide statistically significant results, the extent of the actual changes is minimal. p-values were calculated with ANOVA & Tukey test; * depicts $p < 0.001$.

(D) Quantifications of the rΔΨm change observed in different sized cells within a single cell cycle phase (left) and of the rΔΨm change observed in same sized cells that are in different cell cycle phases (right). Only the cells in range {x} in panel (B) were used ($n = 9x10^4$ cells).

(E) *Top,* DNA content histogram of HUVEC cells stained with propidium iodide. *Bottom,* same as figure (B), but data from HUVEC cells ($n = 3.0x10^5$). Note that the low number of S phase cells precluded reliable analysis from the DNA synthesis phase.

(F) Same as figure (D), but data from HUVEC cells in figure (E).

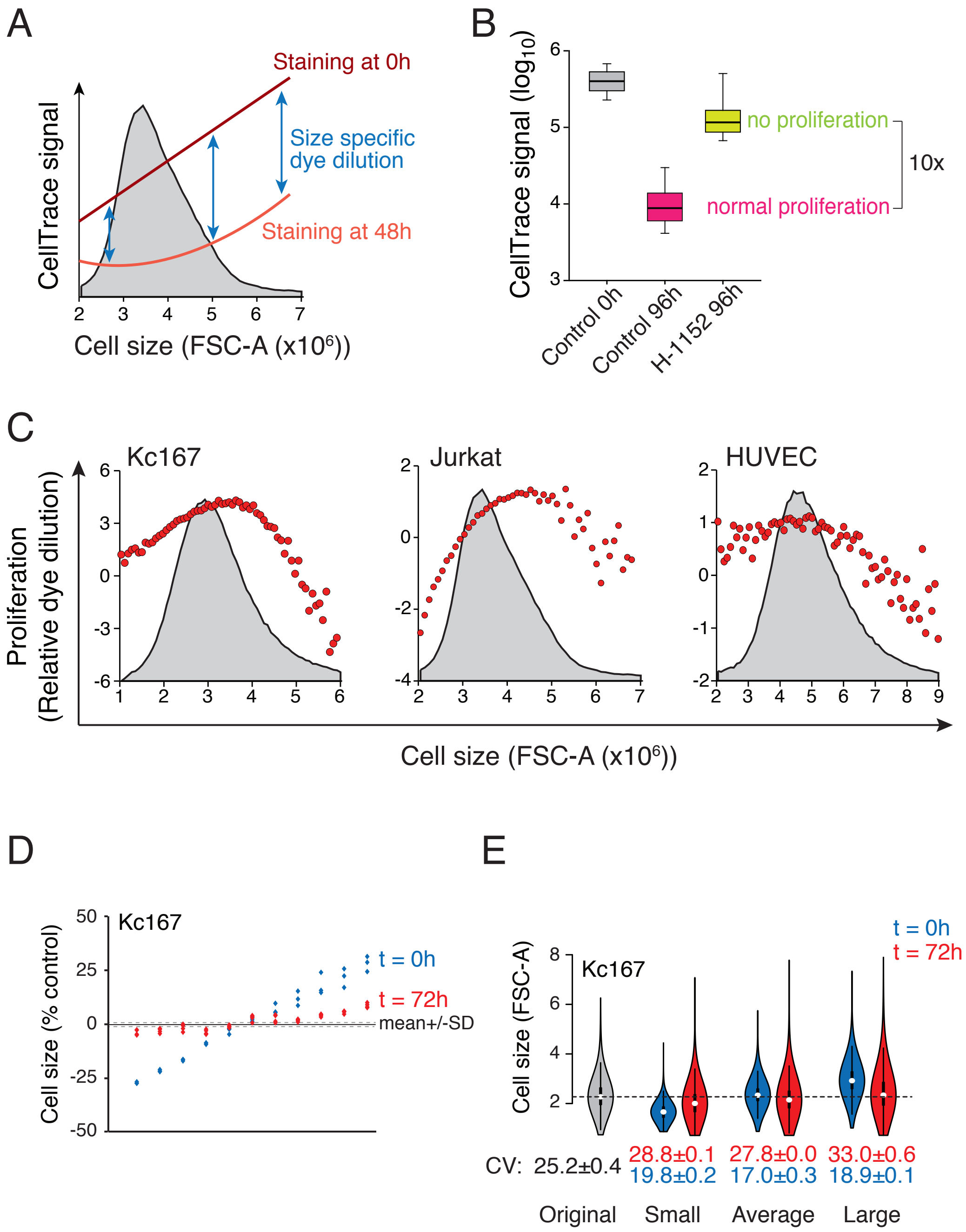

Figure S4. Analysis of optimal cell size by dye dilution and elutriation, related to Figures 3 and 4.

(A) A schematic presentation of how CoSRA approach was used to analyse proliferation. Cells were stained with DDAO-SE, analysed for baseline staining at 0h, grown for 48h and reanalyzed. The relative difference between staining at 0h and at 48h was used as a marker for proliferation.

(B) The suitability of DDAO-SE staining for dye dilution analyses was tested in Jurkat cells by blocking cytokinesis with 2 μM H-1152, a Rho-kinase inhibitor, for 96 h. In the absence of cell division the dye dilution was reduced approximately 10 fold, indicating that the DDAO-SE signal reduction is mainly due to cell proliferation.
(C) Dye dilution based analysis of proliferation in different sized Kc167, Jurkat and HUVEC cells. In all cell types, the proliferative fitness is highest in intermediate sized cells. For all cell types dye dilution was analyzed 48 h after initial staining from > $2x10^5$ cells.
(D) Kc167 cells aim to maintain optimal cell size. Kc167 cells were separated in to size-based subpopulations using centrifugal elutriation and then cultured under normal conditions in order to allow the cells to optimise their size. The mean cell size in each subpopulation immediately after elutriation (0 h, blue) and after 72 h in culture (red) is shown. The data is shown as a relative change in comparison to the unsorted original population (n = 3 in each subpopulation).
(E) Size distributions of the original Kc167 cell population as well as smallest, average sized and largest subpopulations after 0 h (blue) and 72 h (red). Coefficient of variation (CV) is shown below the size distribution plots. Data shown is mean ± SD (n = 3) from $6\text{-}12x10^3$ cells per sample.

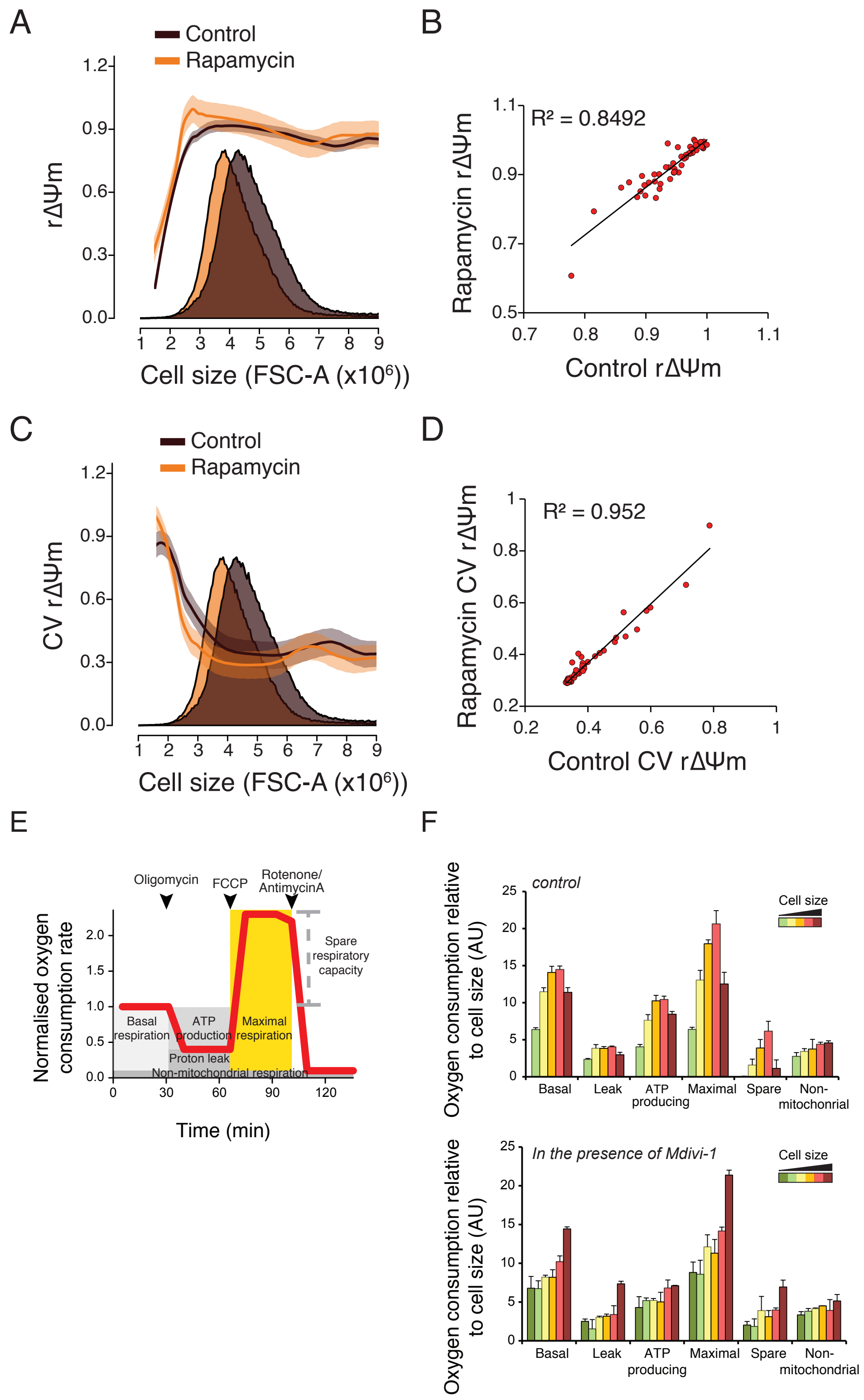

Figure S5. Mitochondrial dynamics and not mTOR regulate cell size scaling of mitochondrial functions , related to Figure 5.

(A) Cell size scaling of rΔΨm in Jurkat cells treated with control (MeOH) or 250 nM rapamycin for 18 h.

(B) Cell size scaling of the cell-to-cell variation in rΔΨm in Jurkat cells treated with control (MeOH) or 250 nM rapamycin for 18 h.

(C) Correlation plot of median rΔΨm values in each cell size bin of samples shown in panel (A). Data is normalised to the average cell size change caused by rapamycin. Pearson correlation coefficient R^2 is also shown.

(D) Correlation plot of rΔΨm variabilities in each cell size bin of samples shown in panel (B). Data is normalised to the average cell size change caused by rapamycin. Pearson correlation coefficient R^2 is also shown. In panels A-D n > 1.7×10^5 cells.

(E) A schematic illustrating how various aspects of oxygen consumption are measured using successive additions of mitochondria perturbing chemicals (arrows) with the Seahorse Flux Analyser.

(F) Oxygen consumption parameters in different sized Kc167 subpopulations separated by centrifugal elutriation. Top panel displays data from control cells and bottom panel displays data from cells treated with 50μM Mdivi-1 for 24h. Same data as in Figures 1E and 5E.

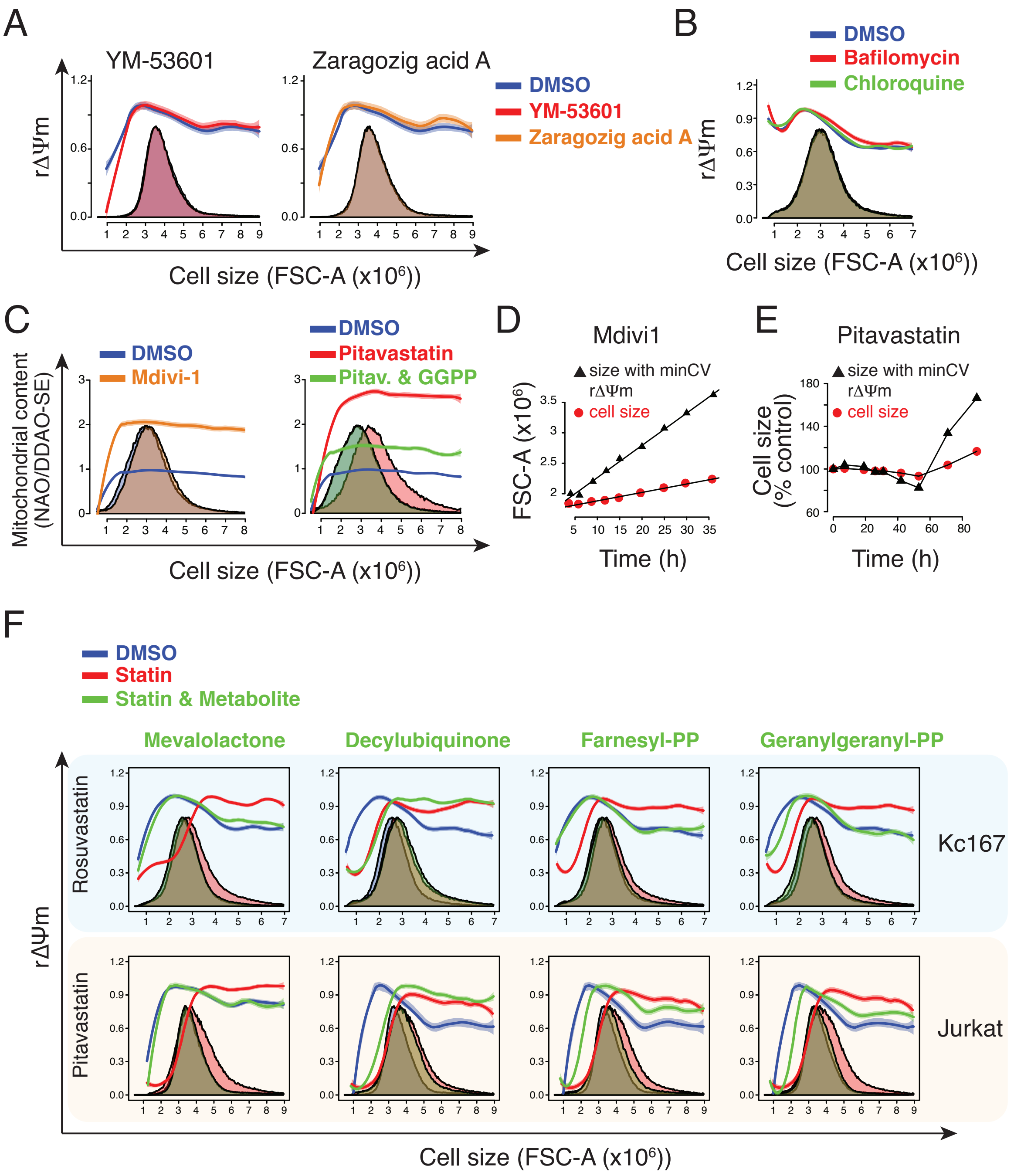

Figure S6. Cell size scaling of rΔΨm requires the mevalonate pathway and cannot be explained by increased mitophagy in larger cells, related to Figure 6.

(A) Cholesterol synthesis inhibitors YM-53601 and Zaragozig acid A do not affect the cell size scaling of rΔΨm in Jurkat cells. Cholesterol synthesis inhibitors were used at 8 μM (YM-53601) and 20 μM (Zaragozig acid A) final concentrations for 72 h.

(B) Autophagy/mitophagy inhibitors do not affect the cell size scaling of rΔΨm in Jurkat cells. Bafilomycin was used at 50 nM concentration and chloroquine at 60 μM for 24 h.
(C) *Left,* Cell size scaling of mitochondrial content in Kc167 cells after 24 h treatment with control (DMSO) or 50 μM Mdivi-1. *Right,* Cell size scaling of mitochondrial content in Kc167 cells after 72 h treatment with control (DMSO), 5 μM pitavastatin or pitavastatin together with geranylgeranylpyrophosphate (GGPP). Note that apart from the very smallest cells both Mdivi-1 and Pitavastatin increase the relative mitochondrial content uniformly in all cell sizes. Mitochondrial content was analyzed using NAO based mitochondrial staining that was normalized to cellular protein content, as measured by DDAO-SE staining. n > $1.0x10^5$ cells in all samples.
(D) Median cell size and the size with minimum variability of mitochondrial membrane potential (min CV of rΔΨm) at various time points after addition of 50 μM Mdivi1 in Kc167 cells.
(E) Median cell size and the size with minimum variability of mitochondrial membrane potential (min CV of rΔΨm) at various time points after addition of 5 μM Pitavastatin in Jurkat cells.
(F) Metabolite rescues of statin induced changes in the cell size scaling of rΔΨm. Statin effects in both Kc167 and Jurkat cells were rescued by mevalolactone (which is converted in to mevalonate) and geranylgeranyl-pyrophosphate (GGPP), but not decylubiquinone. These results indicate that protein prenylation regulates the cell size scaling of rΔΨm. Pitavastatin was used at 5 μM concentration and rosuvastatin at 40 μM concentration. n > $1.0x10^5$ cells in all samples.
Note that several other cellular perturbations, including mtUPR induction and reactive oxygen species, did not display major changes in the cell size scaling of rΔΨm (data not shown).

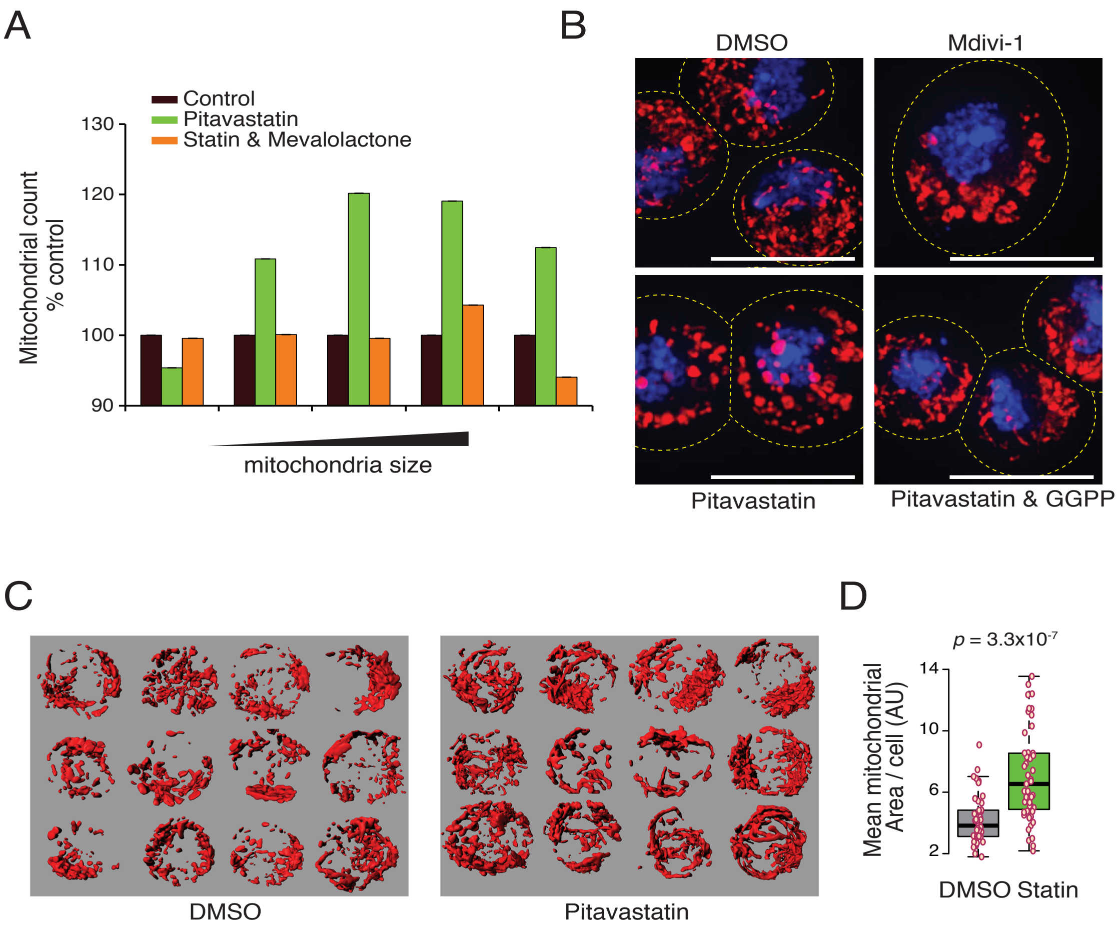

Figure S7. Mitochondrial size and morphology homeostasis require the mevalonate pathway activity, related to Figure 7.

(A) Analysis of mitochondrial sizes in Kc167 cells by flow cytometry. Pitavastatin treatment was rescued by mevalonate (supplied as mevalolactone). Mitochondrial size analysis data represents mean ± SD (n = 3 technical replicates).

(B) Representative maximum-intensity projections of Kc167 cells stained with DAPI (nucleus, blue) and MitoTracker Red (mitochondria, red) after treatment with indicated chemicals. Statin treatment was 72 h and Mdivi-1 treatment was 24 h. Note that Kc167 cells do not form long, elongated mitochondria and highly connected mitochondrial network even in the presence of Drp1 inhibition. Nonetheless, mitochondrial size increases, when mitochondrial division is inhibited. Scalebar 10 μm.

(C) Representative 3D surface projections of mitochondrial network of live Jurkat cells after 72 h treatment with pitavastatin. Cells were stained with MitoTracker Green.

(D) Quantification of mean mitochondrial surface area in each Jurkat cell from samples shown in panel (D) (n = 48 (DMSO) and 51 (Pitavastatin)).

SUPPLEMENTAL EXPERIMENTAL PROCEDURES

Transfections

Kc167 dsRNA transfections were carried out as before (Bjorklund et al., 2006; Miettinen et al., 2014). To construct the long dsRNAs used for RNAi, the following primers were used: taatacgactcactatagggCTCATGATAGCTGAACGCCA and taatacgactcactatagggGTCCGTTTTCAGCAGACTGG for Drp1 as well as taatacgactcactatagggTGAGCAAATACCCCCAAAAG and taatacgactcactatagggGATCTGGAGCGGTGATTTGT for Marf (mitofusin) knockdown.

Jurkat siRNA transfections were carried out using two independent 40nM siRNAs (IDT DNA) for each target and the TransIT-TKO transfection reagent (Mirus Bio) according to manufacturer's instructions. MFN2 was knocked down with two pairs of siRNAs GCAUGGUACCAAGGAGUUAAGUUGA (sense) / UCAACUUAACUCCUUGGUACCAUGCUG (antisense) and GGUUUACUGCGAGGAAAUGCGUGAA (sense) / UUCACGCAUUUCCUCGCAGUAAACCUG (antisense). DNM1L (hsDRP1) was knocked down with GGUUUACUGCGAGGAAAUGCGUGAA (sense) / UUCACGCAUUUCCUCGCAGUAAACCUG (antisense) and AGGAUAUUGAGCUUCAAAUCAGAGA (sense) / UCUCUGAUUUGAAGCUCAAUAUCCUUA (antisense) oligonucleotides.

Cellular proteome scaling with cell size

The protein abundances for the NB4 leukemia cells proteome were from a previously published dataset, where unperturbed NB4 cells were separated by cell size using elutriation and analysed by label-free mass spectrometry (Ly et al., 2014). For the analysis of organelle scaling, the measured proteins were classified to subcellular structures based on GeneOntology annotations obtained from Ensembl Genome Browser. For each subcellular structure, data was normalised to the smallest cell size fraction as well as total protein abundance in each fraction to obtain organelle scaling normalised to total protein.

Flow cytometry measurements

Although ratiometric dyes are the most accurate for measurement of membrane potential (Novo et al., 1999), JC-1 as well as non-ratiometric $\Delta\Psi m$ responsive dyes have potential limitations (Perry et al., 2011). We thus qualitatively confirmed the the JC-1 based results using TMRE based staining of $\Delta\Psi m$ and normalization of this signal to total amine reactive cellular protein content using DDAO-SE. Note that DDAO-SE provides a reliable measure of cell size

(Miettinen and Bjorklund, 2015) and an accurate estimate for total cellular protein content (Kafri et al., 2013). Intracellular pH was measured using the ratiometric SNARF-1 dye, plasma membrane potential using DiBAC4(3) and apoptosis using polarity-sensitive annexin-based biosensor (pSIVA). The fluorescent dyes used were (with final concentrations): DDAO-SE (0.5 μM for protein content and 1 μM for dye dilution), DiBAC4(3) (1 μM), JC-1 (2 μM, except 0.5 μM for hepatocytes and 4 μM for HUVECs). MitoTracker Green FM (50 nM, except 200 nM for mitochondria isolation), MitoTracker Red CMXRos (125 nM), NAO (100 nM), Nuclear-ID Red (20 μM), propidum iodide (30 μg/ml), pSIVA-IANBD (diluted as per suppliers instructions), SNARF-1 (5 μM), TMRE (100 nM) and CellMask Deep Red (1:1000 dilution).

All fluorescence intensities, cell counts and sizes were measure using Accuri C6 flow cytometer (Accuri, now Becton-Dickinson) equipped with 488 nm and 640 nm lasers and standard optical filters (FL1 533/30 nm, FL2 585/40 nm, FL3 >670 nm, FL4 675/25 nm). Electrical current exclusion method was performed using CASY TT (Roche) as before (Miettinen et al., 2014). Flow cytometry measurement of cell size is based on Mie theory on light scattering and usually employ the forward scatter (FSC) pulse area (FSC-A). To validate the relationship between our instrument's (Accuri C6) FSC-A values and electronic cell size measurements (Casy TT instrument), we separated multiple cell types by size using elutriation and measured the average sizes of these subpopulations by flow cytometry and electronic volume measurement. With each cell line, we observed near-perfect correlation's with FSC-A and cell diameter (Pearson R^2=0.95-0.99, Figure S1C). The FSC-A and cell diameter measurements correlated well over the whole range of measured cell sizes from yeast to human U2OS cell (R^2=0.99, Figure S1C). Notably, commercially available size calibration beads also displayed strong size correlation (R^2=0.99), but the slope of the regression line was substantially different from cells, precluding bead-based calibration for cell volumes (Figure S1C). Correlations between FSC-A values were best with cell diameter (R^2=0.99) rather than cell area (R^2=0.97) or volume (R^2=0.90) measurements calculated from electronic volume measurements (Figure S1D), consistent with previous analyses using beads (Cookson et al., 2010). In conclusion, cell size measurements using FSC-A values, at least with our flow cytometer, are accurate, but more closely reflect changes in cell diameter than in cell volume. Note that the ratiometric measurement of JC-1 nevertheless normalises the r$\Delta\Psi$m to mitochondrial and cell volume.

Computational cell size relation analysis (CoSRA) of ΔΨm variability and other cellular parameters

The CoSRA approach was used to analyse cell size scaling of several cellular parameters using the same principle as with ΔΨm analysis. For example, we analysed mitochondrial content as a function of total protein. For this, cells were stained with DDAO-SE, the DDAO-SE signal (FL4-A) was fractionated in to bins instead of FSC-A followed by calculation of the median JC-1 monomer signal for each bin. We also validated that the cell size scaling of rΔΨm observed with JC-1 staining is not due to differential staining kinetics or dye diffusion in small and large cells. This was done by analyzing Kc167 cells through the JC-1 dye incubation. The flow cytometer data was then sorted for different sized cells and the rΔΨm of small, medium and large cells was plotted as a function of time using the flow cytometer time stamp for each cell.

CoSRA based approach was also used to investigate the cell size scaling of the cell-to-cell variability in rΔΨm. The analysis was carried out as above with the exception that fluorescence signal variabilities (expressed as coefficient of variation (CV), which is standard deviation divided by the mean) were calculated for each cell size bin instead of the median fluorescence. The dependency of the CV values on then number of cells in each bin was tested by random sampling of the total population in each bin. The CV rΔΨm curve robustly retained the observed shape including the global minima when equal number (200 or more cells) for each bin was sampled and the CV ΔΨm analysed from these (data not shown). Thus, the cell size with minimum variation of CoSRA-rΔΨm signal cannot be simply explained by the number of cells measured.

Analyzing the contribution of cell size and cell cycle to the rΔΨm

When analysing ΔΨm as a function of cell cycle, median JC-1 ratios (rΔΨm) were plotted by fractionating the population based on DNA content, which was measured using Nuclear-ID Red staining. Cell cycle phases were gated manually based on the resulting DNA histogram. Kc167 cells could not be analysed in this way due to the lack of a suitable live cell DNA stain. Comparison of rΔΨm behavior as a function of cell size and cell cycle in Figure S4 was done as follows. First, a cell size scale during which the rΔΨm scale in relatively linear fashion in all cell cycle phases was selected and marked as {x}. Second, the rΔΨm was quantified from each cell cycle phase using all the cells within {x}. These quantifications, in which the influence of cell size in minimized, suggested only marginal difference in rΔΨm between different cell cycle phases (see Figure S4C). Third, the extent to which rΔΨm changes within

a single cell cycle phase as cells grow in size (in the size range {x}) was compared to the change in rΔΨm when examining cells of same size {x} that are in different cell cycle phases. This indicated that the size dependent change in rΔΨm is several fold larger than the change caused by cells being in a different cell cycle phase, as shown in Figures S4D and S4F.

Also the rate of rΔΨm decrease in each cell cycle phase was analyzed. This was carried out by fitting linear regression lines were to the raw data in Figure S4A so that the smallest and the largest cells, in which ΔΨm does not change quasi-linearly with cell size, were excluded. The slopes of these regression lines were then compared, as shown in Figure S4C.

Size separation by centrifugal elutriation

To examine size dependent changes using population based assays, we separated cells into size-based subpopulations using centrifugal elutriation. As centrifugal elutriation separates cell based on their size and sedimentation density (Banfalvi, 2008), and as cell density is relatively constant (Feijo Delgado et al., 2013), elutriation separates cells essentially based on their size. Approximately $3x10^8$ were resuspended in 3 ml of PBS with 1% FBS. Cells were loaded into counterflow centrifugal elutriator (Beckman JE-5.0/JE), equipped with a standard elutriation chamber and a Cole–Parmer MasterFlex Model 900-292 peristaltic pump. The centrifuge was operated at 3000 rpm and the flow rate was initially set to 21 ml/min. After cells were loaded into the elutriation chamber, 50 ml fractions were collected with increasing flow rates. Collected cells were resuspended in culture medium and cell size and counts were analysed using flow cytometry. Mitochondrial mass and membrane potential from elutriated fractions were measured using MitoTracker Green FM and MitoTracker Red CMXRos, respectively. Flow cytometry measured MitoTracker intensities were normalised to mean FSC-A values.

Oxygen consumption measurements

Oxygen consumption was measured using XF24 Extracellular Flux Analyzer (Seahorse Bioscience). The measurement plates were coated with poly-L-lysine and 80 000 Kc167 cells were plated on each well 2h before the analysis. The analysis was carried out in Schneider's cell culture medium with 10% FBS at room temperature. Oxygen consumption was measured every 6 minutes and the following injections were performed after every 4 measurements: (1) 1μM Oligomycin, (2) 1μM FCCP and (3) 2 μM Rotenone and 2 μM Antimycin A. Figure S5E explains the how each oxygen consumption related parameter was obtained. For each replicate the four oxygen consumption measurements between each injection were averaged, after which

the respiratory parameters were calculated. All the results were normalised to the subpopulation cell size (FSC-A). The analysis was also carried out after treating cells with the mitochondrial division inhibitor Mdivi-1 (50 μM) for 24h or Pitavastatin (5 μM) for 72h. Mdivi-1 and pitavastatin were present for the whole duration of the oxygen consumption assay.

Proliferation measurements

For proliferation based fitness measurements, Kc167, Jurkat and HUVEC cells were first separated into size-based subpopulations using centrifugal elutriation. The average cell size in each subpopulation was measured using flow cytometry and equal cell count of each subpopulation was taken for further culture in normal conditions. The final data is presented as relative cell counts after indicated culture time (y-axis) as a function of the initial subpopulation cell size (x-axis). The total cell population histogram is also show for reference. For Kc167 cells the differences in proliferative capacity of different sized cells were highlighted by presenting the data as a box plot.

The effect of mitochondrial membrane potential on cell proliferation was analyzed in Jurkat cells by first using centrifugal elutriation to separate two different sized subpopulations. These cells were then stained with TMRE and cells with high, medium and low r$\Delta\Psi$m (thus taking cell size into account in assessing the $\Delta\Psi$m, see schematic in Figure 2E) were separated using flow sorting (Influx Cell Sorter, Becton Dickinson) and cell sizes were reanalyzed in the separated populations to validate that r$\Delta\Psi$m based separation did not change the average cell size. Equal cell counts of these r$\Delta\Psi$m separated cells were then cultured for 100h in normal growth conditions, after which cells were recounted.

When analyzing cell proliferation using the CoSRA method (see above), cells were first stained using DDAO-SE and the baseline staining levels were measured using flow cytometer. The cells were then cultured normally for 48h after which cells were reanalyzed for DDAO-SE stain dilution (See Figure S5A). The relative dye dilution was calculated for each cell size bin and illustrated as before. If cells of all sizes proliferate at an equal rate, the relative dye dilution should be the same in all cells. High dye dilution indicates faster proliferation (Miettinen and Bjorklund, 2015).

Protein synthesis measurements

Jurkat cells were stained with Click-iT Plus OPP Alexa Fluor 488 Protein Synthesis Assay Kit (LifeTechnologies) and MitoTracker Red CMXRos. OPP and MitoTracker Red were added to

to 20 μM and 200 nM final concentration, respectively and incubated for 30 min. Cells were fixed with 3.7% formaldehyde and processed as instructed in the OPP assay kit. Cells were analysed using flow cytometry.

Western blots

Jurkat siRNA efficiency was validated by western blot. The cells were lysed directly in Laemmli buffer, sonicated and analyzed on a SDS-PAGE gel. The antibodies were obtained from Abcam (DRP1 (ab56788) and MFN2 (ab124773)) and Cell Signaling Technology (GAPDH (#5174)), used at the concentrations recommended by suppliers and detected using infrared-dye conjugated secondary antibodies and LICOR Odyssey detection system.

Isolation of mitochondria

Cells were stained with 200 nM NAO for 15 min and collected by centrifugation. Crude mitochondrial fraction was isolated after lysis in 10 mM Tris–MOPS (pH to 7.4), 1mM EGTA, 0.2M Sucrose using dounce homogeniser. Nuclear pellet was removed by centrifugation at 600xg for 5 min. Mitochondrial fraction was obtained by centrifugation 1t 7000xg for 5 min. The resulting pellet was suspended in PBS and immediately analysed by flow cytometry (Mattiasson, 2004; Petit et al., 1990). Mitochondria were identified by measuring NAO signal (FL1-A) and forward scatter (FSC-A). For mitochondrial size analysis, FSC-A ranges of increasing sizes were selected and the percentage of mitochondria relative to control sample in each size range was calculated.

Microscopy

For JC-1 staining validations, live Kc167 and Jurkat cells were imaged using OMX structured illumination microscope (GE Healthcare) with standard filters (528/48 nm and 609/37 nm filters for JC-1 monomer and aggregate, respectively) and 100x objective. Microscopy slides were coated with 0.1% poly-L-lysine. Cells were imaged in the presence of JC-1 stain in complete media. For examination of mitochondrial morphology, Kc167 and Jurkat cells were treated with indicated chemicals and moved to coverslips coated with 0.1% poly-L-lysine. The cells were stained with MitoTracker Red CMXRos, washed twice with PBS and fixed with 4% paraformaldehyde. Cells were then permeabilized with 0.1% Tween in PBS, washed with TBS

and the nuclei were stained with 4',6-diamidino-2-phenylindole (DAPI) followed by two washes with TBS. Cells were mounted using VectaShield mounting media (VectorLabs).

For microscopic analysis of isolated mitochondria, cells were incubated with 200 nM MitoTracker green for 30 min. Mitochondria were isolated and imaged as shown below. For live cell imaging, Jurkat cells were stained with 50 nM MitoTracker Green and 1:1000 diluted CellMask Deep Red for 15 min in complete medium at 37°C. Cells were centrifuged for 3 min at 500xg and resuspended in fresh complete medium without the dyes but with DMSO or Pitavastatin and added on polylysine coated coverslips for imaging. U2OS cells expressing mCherry-GFP were imaged in the absence of any stains. Mitochondrial fragmentation was induced with 2 μM FCCP for 1 h. Cells were imaged with DeltaVision widefield deconvolution microscope using standard filters (DAPI, and TRITC) and 100X objective.

Image processing

All images were deconvoluted using DeltaVision SoftWoRx software. Image processing was done using ImageJ and Imaris (v. 7.2.1, Bitplane.com). Binary images of mitochondria stained with MitoTracker Red were created using the binary processing tool in ImageJ with default settings for all images. For surface rendering, individual cells were cut out from widefield images and contrast was adjusted to 0.5% saturated pixels per slice in ImageJ. 3D rendering of MitoTracker Green stained mitochondrial network was performed with Imaris software. Surface smoothing was used with 0.5 μm Surfaces Area Detail Level and thresholding adjusted to half-maximal intensity. Mitochondrial size quantifications were exported from Imaris and analysed using R. Maximum intensity projections were created in ImageJ.

SUPPLEMENTAL REFERENCES

Banfalvi, G. (2008). Cell cycle synchronization of animal cells and nuclei by centrifugal elutriation. Nat Protoc *3*, 663-673.

Bjorklund, M., Taipale, M., Varjosalo, M., Saharinen, J., Lahdenpera, J., and Taipale, J. (2006). Identification of pathways regulating cell size and cell-cycle progression by RNAi. Nature *439*, 1009-1013.

Cookson, N.A., Cookson, S.W., Tsimring, L.S., and Hasty, J. (2010). Cell cycle-dependent variations in protein concentration. Nucleic Acids Res *38*, 2676-2681.

Feijo Delgado, F., Cermak, N., Hecht, V.C., Son, S., Li, Y., Knudsen, S.M., Olcum, S., Higgins, J.M., Chen, J., Grover, W.H.*, et al.* (2013). Intracellular water exchange for measuring the dry mass, water mass and changes in chemical composition of living cells. PLoS One *8*, e67590.

Kafri, R., Levy, J., Ginzberg, M.B., Oh, S., Lahav, G., and Kirschner, M.W. (2013). Dynamics extracted from fixed cells reveal feedback linking cell growth to cell cycle. Nature *494*, 480-483.

Ly, T., Ahmad, Y., Shlien, A., Soroka, D., Mills, A., Emanuele, M.J., Stratton, M.R., and Lamond, A.I. (2014). A proteomic chronology of gene expression through the cell cycle in human myeloid leukemia cells. Elife *3*, e01630.

Mattiasson, G. (2004). Flow cytometric analysis of isolated liver mitochondria to detect changes relevant to cell death. Cytometry A *60*, 145-154.

Miettinen, T.P., and Bjorklund, M. (2015). Mevalonate Pathway Regulates Cell Size Homeostasis and Proteostasis through Autophagy. Cell Rep *13*, 2610-2620.

Miettinen, T.P., Pessa, H.K., Caldez, M.J., Fuhrer, T., Diril, M.K., Sauer, U., Kaldis, P., and Bjorklund, M. (2014). Identification of transcriptional and metabolic programs related to mammalian cell size. Curr Biol *24*, 598-608.

Novo, D., Perlmutter, N.G., Hunt, R.H., and Shapiro, H.M. (1999). Accurate flow cytometric membrane potential measurement in bacteria using diethyloxacarbocyanine and a ratiometric technique. Cytometry *35*, 55-63.

Perry, S.W., Norman, J.P., Barbieri, J., Brown, E.B., and Gelbard, H.A. (2011). Mitochondrial membrane potential probes and the proton gradient: a practical usage guide. Biotechniques *50*, 98-115.

Petit, P.X., O'Connor, J.E., Grunwald, D., and Brown, S.C. (1990). Analysis of the membrane potential of rat- and mouse-liver mitochondria by flow cytometry and possible applications. Eur J Biochem *194*, 389-397.
